# Supplementary material for: Ancient human genome-wide data from a 3000-year interval in the Caucasus corresponds with eco-geographic regions
Source: Nat Commun. 2019 Feb 4;10:590. doi: 10.1038/s41467-018-08220-8 (PMC6360191; doi:10.1038/s41467-018-08220-8)
Supplement: Supplementary file 1 — Supplementary Information [file 41467_2018_8220_MOESM1_ESM.pdf]

Supplementary Information

Wang et al.,
Ancient human genome-wide data from a 3000-year interval in the Caucasus corresponds with eco-geographic regions

Supplementary Figures and Figure legends

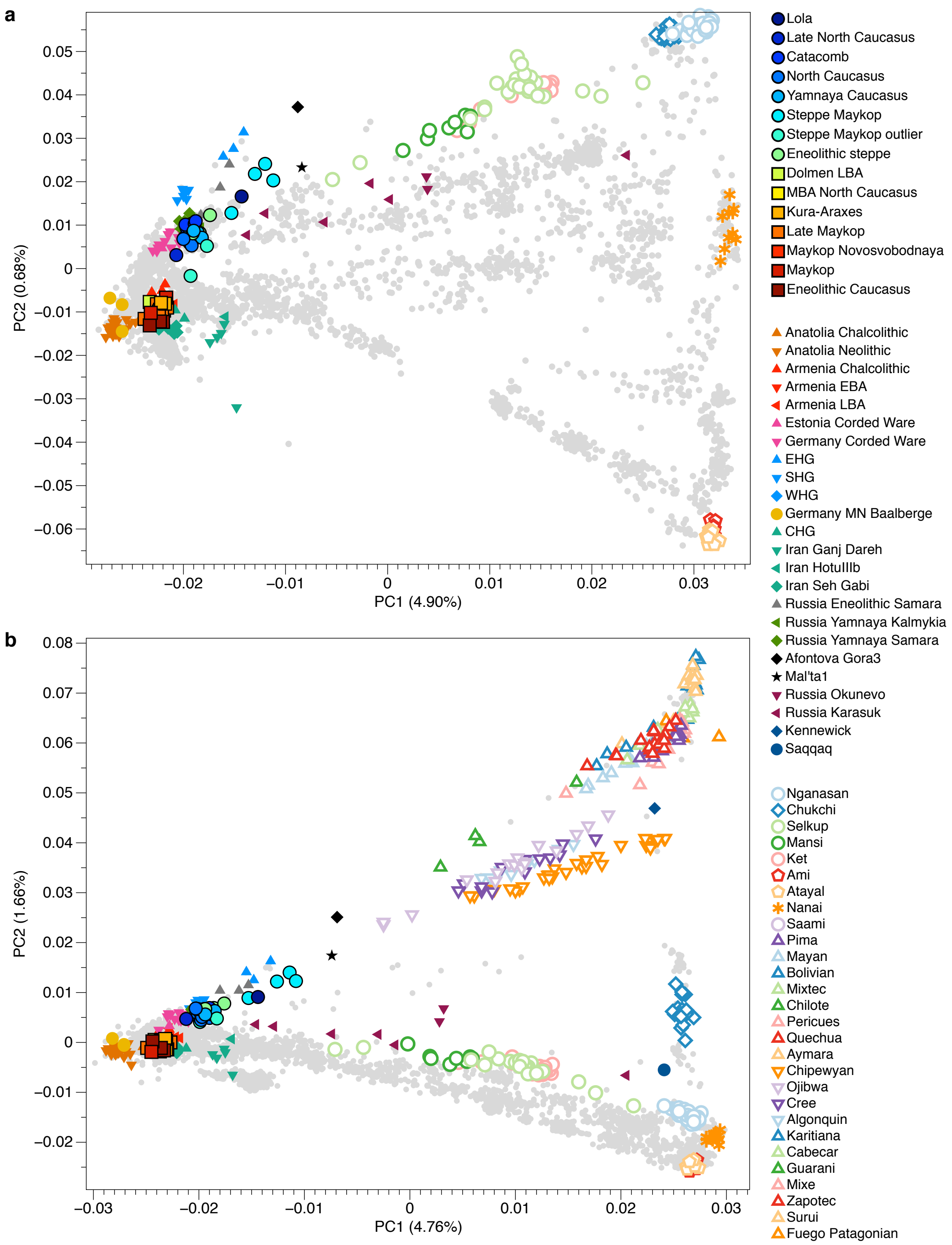

Supplementary Fig. 1. Additional PCA analyses including Eurasian and Native American populations. PCA plots with 2036 Eurasian individuals from 147 populations (Panel a) and with additional 223 Native American individuals from 29 populations (Panel b) highlighting the shared ANE ancestry between prehistoric individuals from the Eurasian steppe zone and Native Americans.

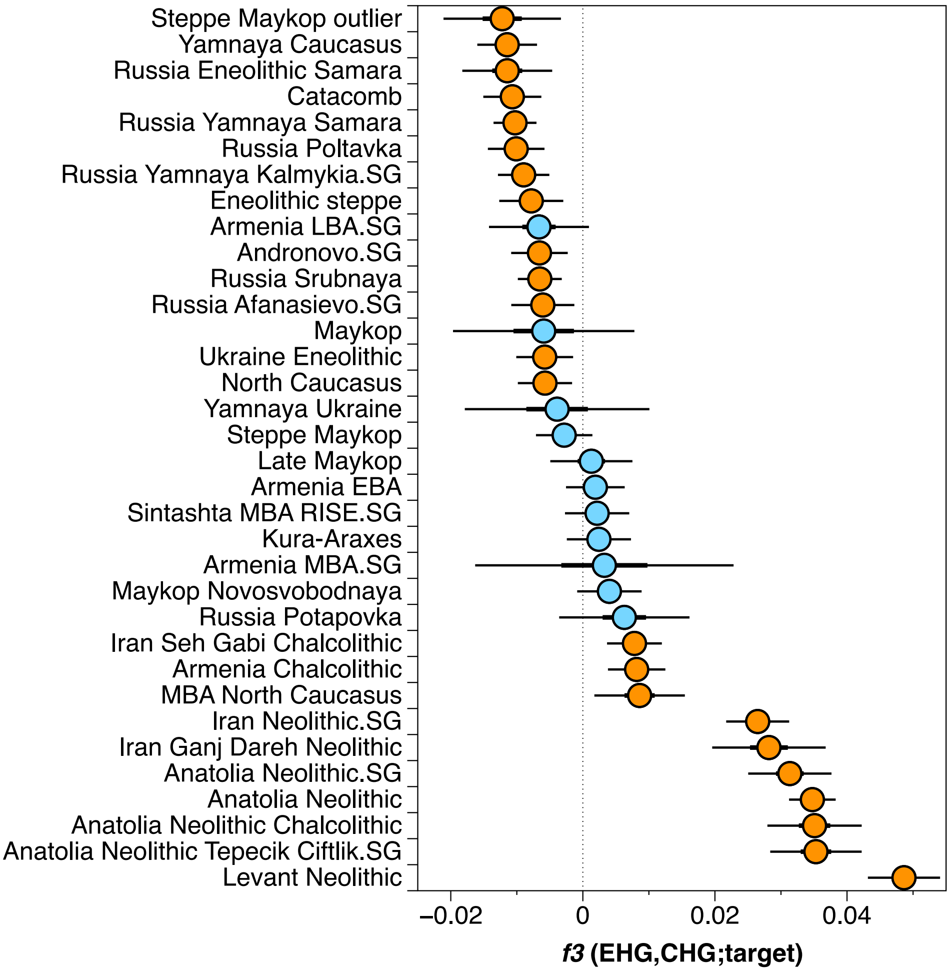

Supplementary Fig. 2. Results of admixture f_3-statistics of the form f_3(EHG, CHG; target). Significant Z-scores are highlighted in orange, f_3-values include one and three standard errors. Target populations are given on the Y-axis.

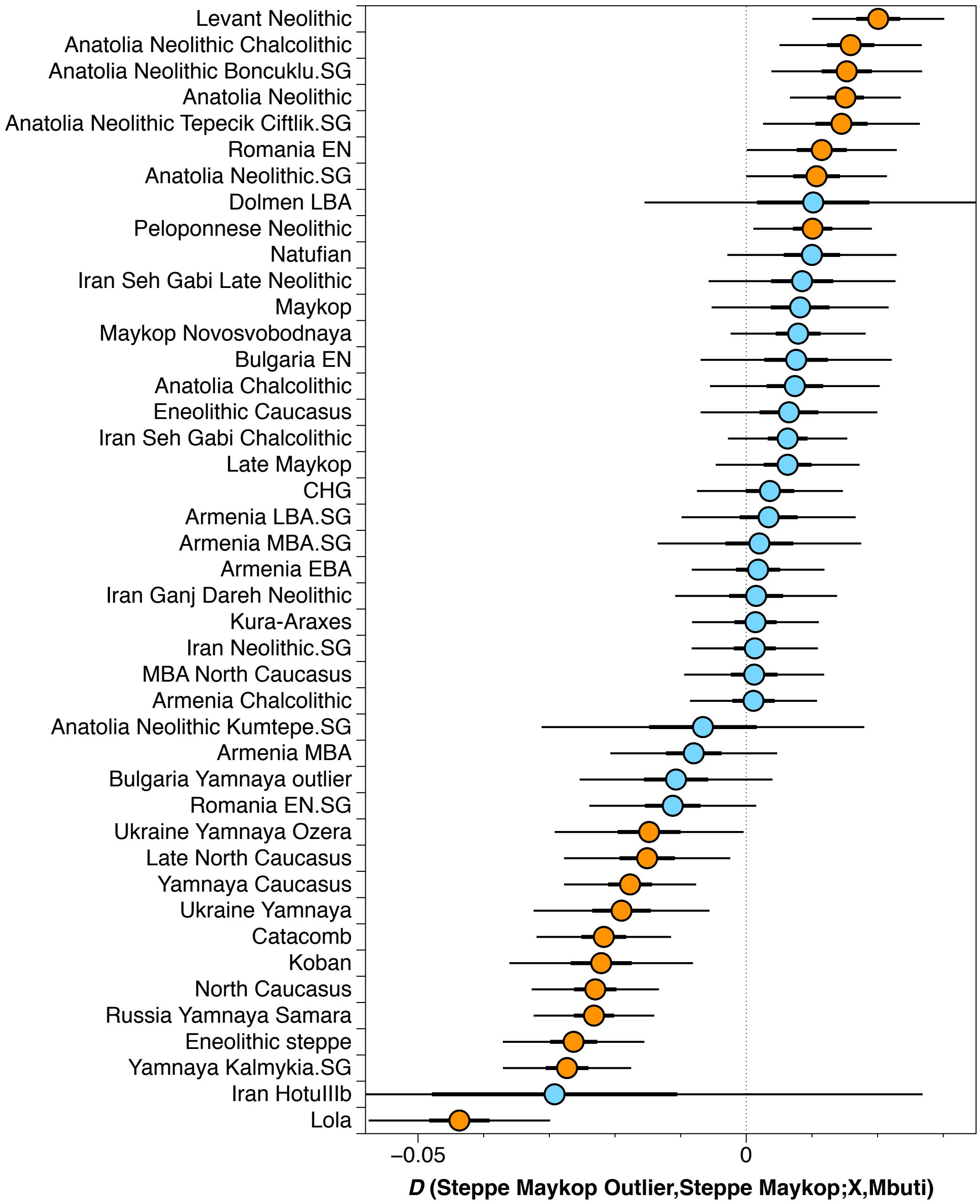

Supplementary Fig. 3. Results of *D*-statistics of the form *D* (Steppe Maykop outlier, Steppe Maykop; X, Mbuti). Significant Z-scores are highlighted in orange, *D*-values include one and three standard errors. Test populations X are given on the Y-axis.

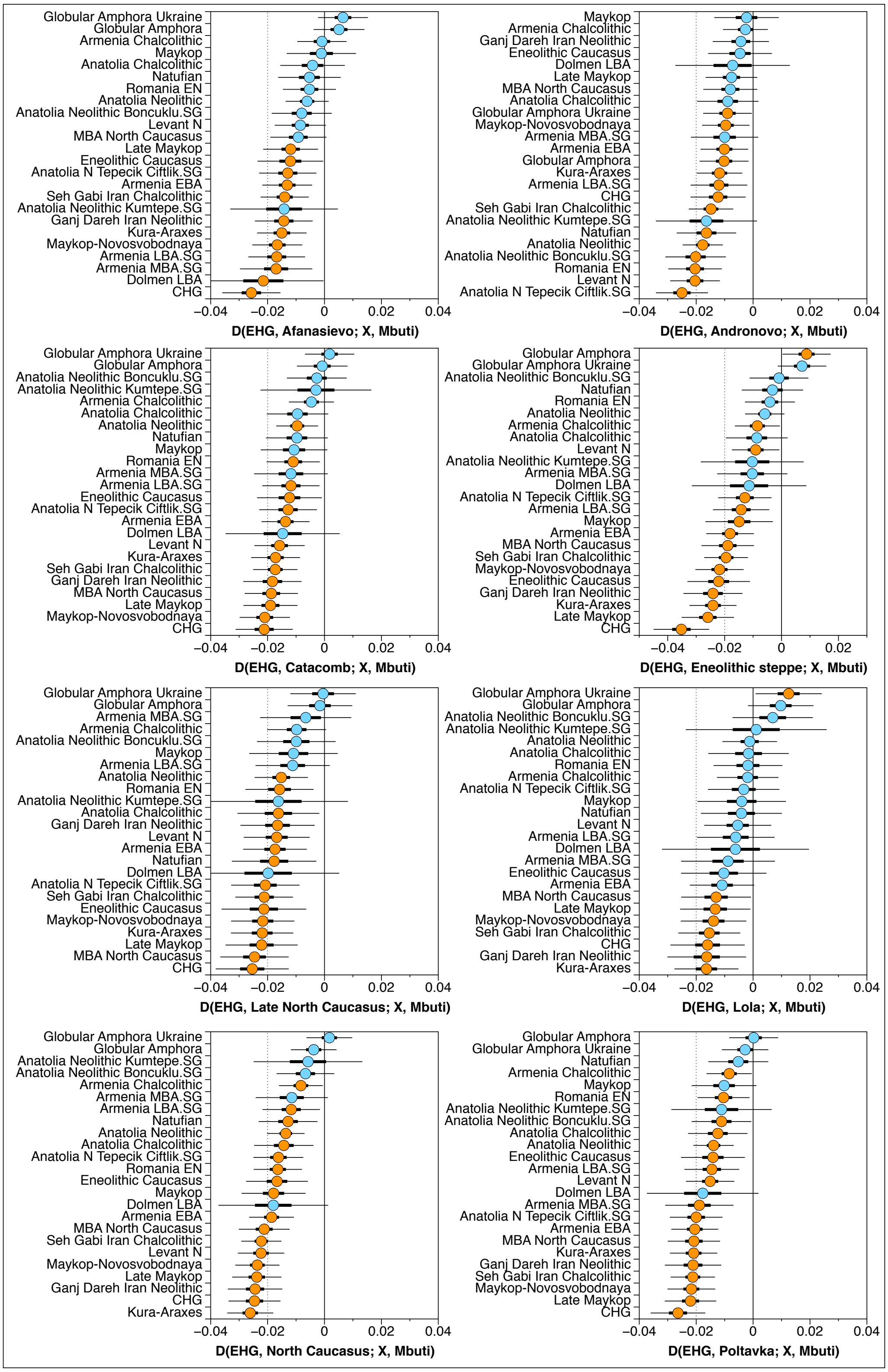

Supplementary Fig. 4. Results of *D*-statistics of the form *D*(EHG, steppe ancestry group; X, Mbuti). Significant Z-scores are highlighted in orange, *D*-values include one and three standard errors. Test populations X are given on the Y-axis.

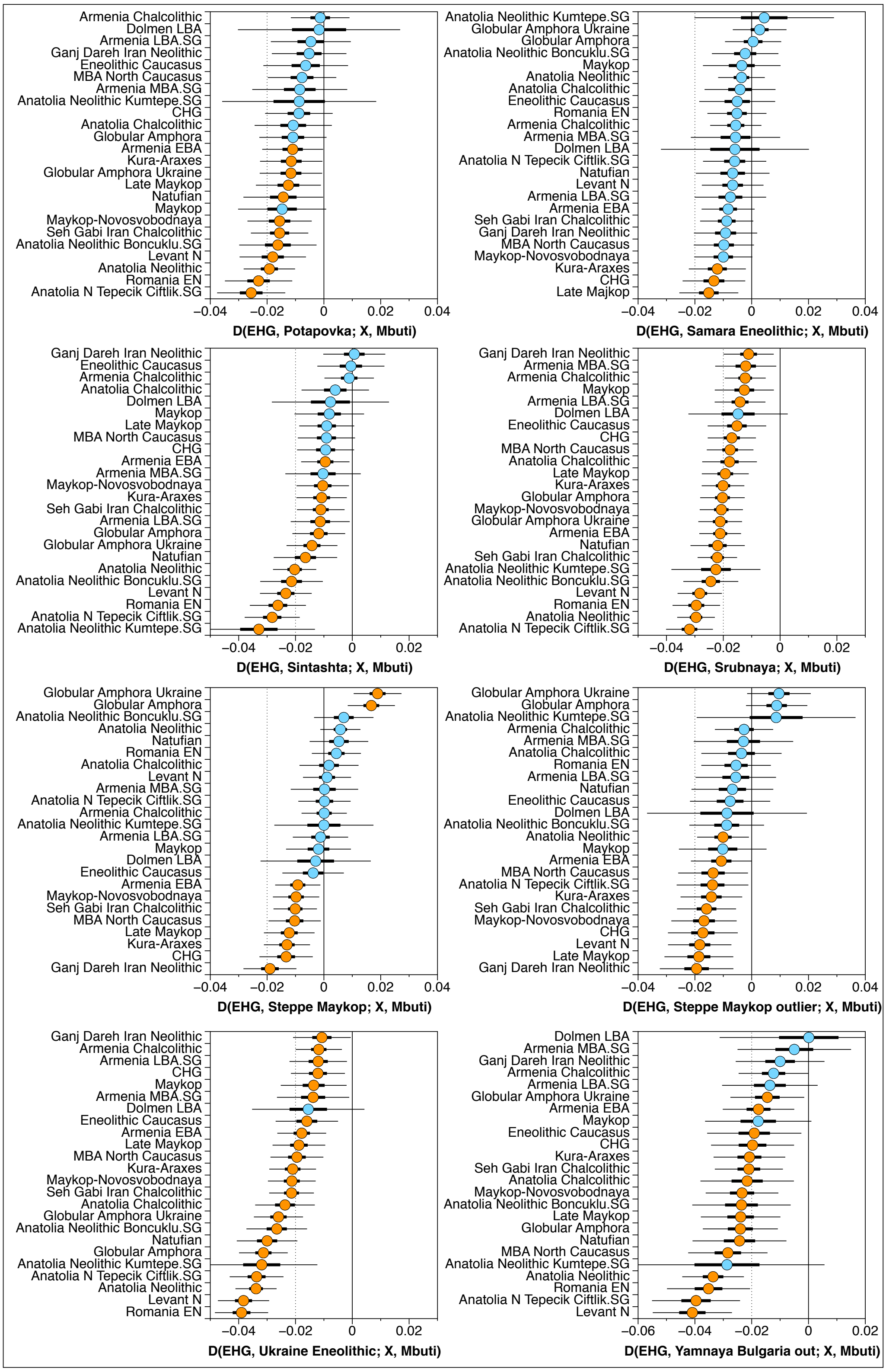

Supplementary Fig. 4; continued. Results of *D*-statistics of the form *D*(EHG, steppe ancestry group; X, Mbuti). Significant Z-scores are highlighted in orange, *D*-values include one and three standard errors. Test populations X are given on the Y-axis.

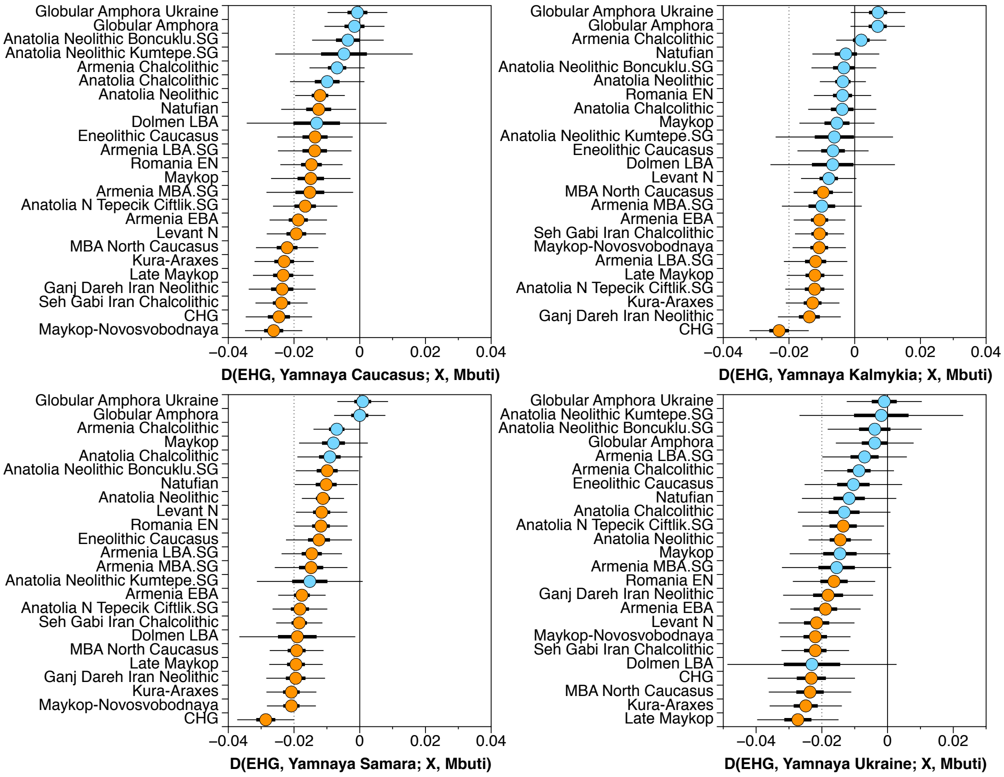

Supplementary Fig. 4; continued. Results of *D*-statistics of the form *D*(EHG, steppe ancestry group; X, Mbuti). Significant Z-scores are highlighted in orange, *D*-values include one and three standard errors. Test populations X are given on the Y-axis.

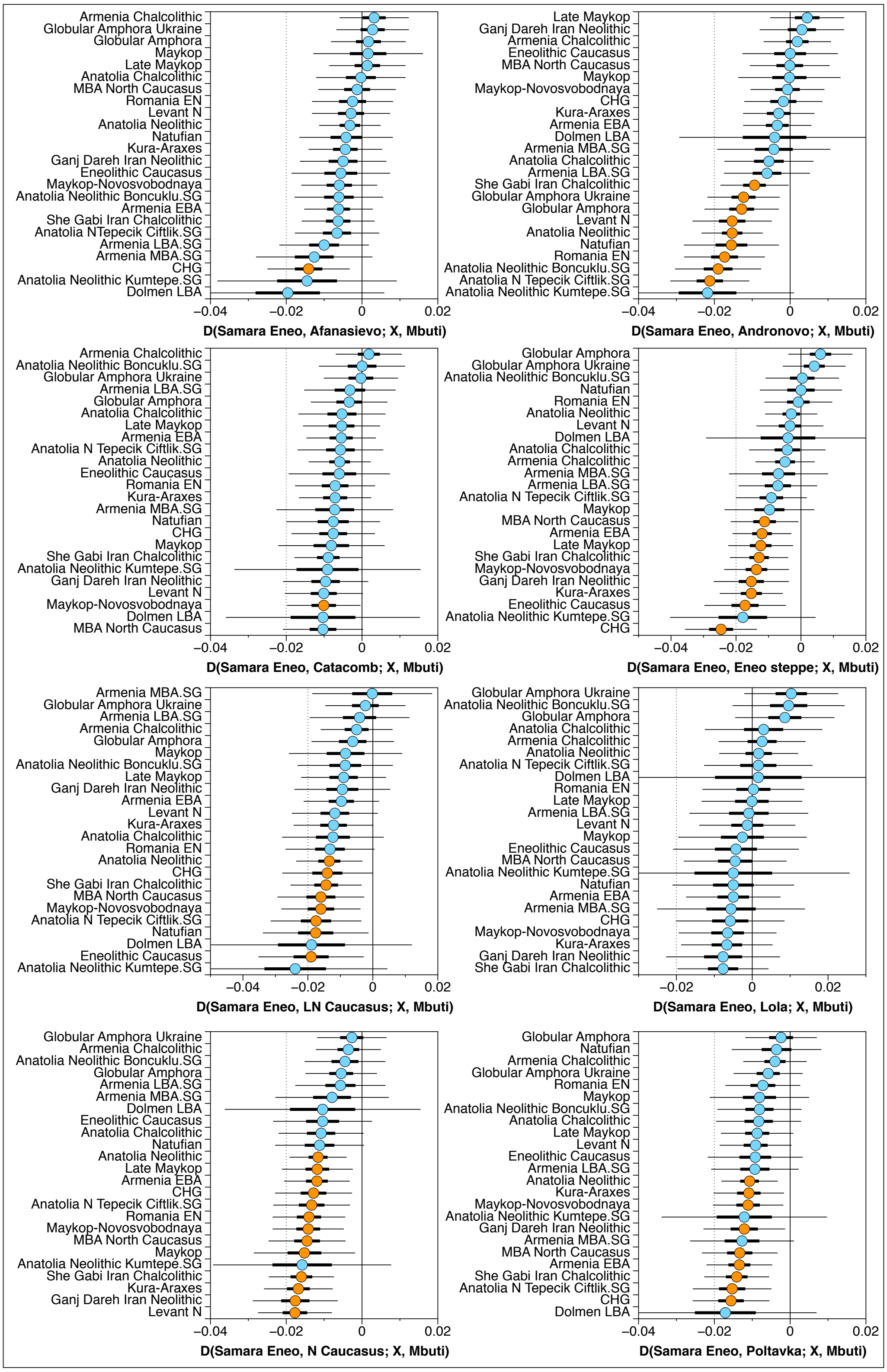

Supplementary Fig. 5. Results of *D*-statistics of the form *D* (Samara Eneolithic, steppe ancestry group; X, Mbuti). Significant Z-scores are highlighted in orange, *D*-values include one and three standard errors. Test populations X are given on the Y-axis.

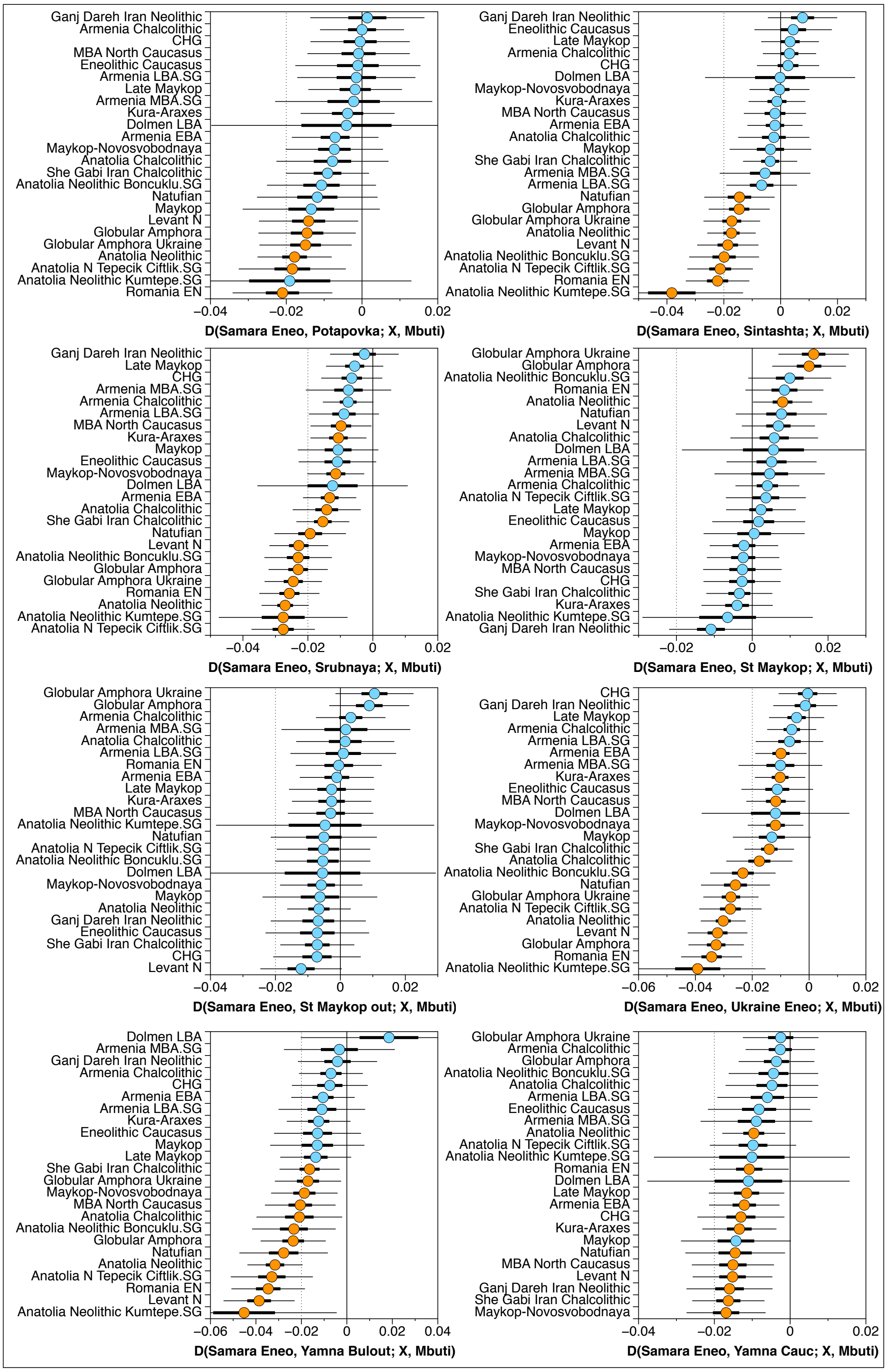

Supplementary Fig. 5; continued. Results of *D*-statistics of the form *D* (Samara Eneolithic, steppe ancestry group; X, Mbuti). Significant Z-scores are highlighted in orange, *D*-values include one and three standard errors. Test populations X are given on the Y-axis.

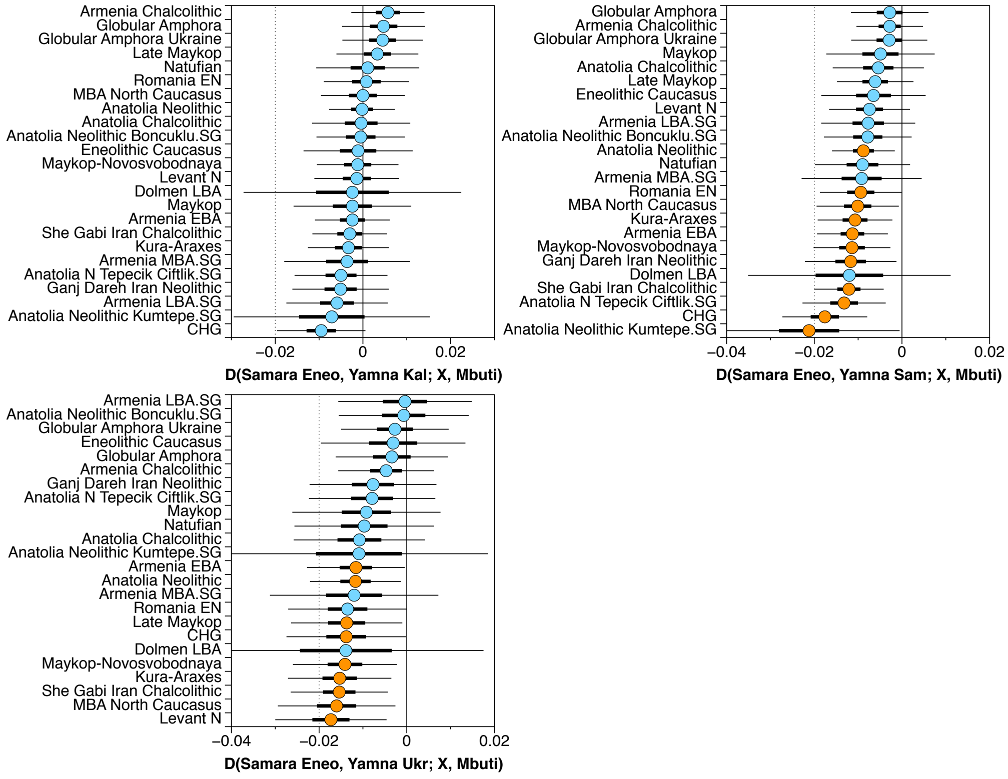

Supplementary Fig. 5; continued. Results of *D*-statistics of the form *D* (Samara Eneolithic, steppe ancestry group; X, Mbuti). Significant *Z*-scores are highlighted in orange, *D*-values include one and three standard errors. Test populations X are given on the Y-axis.

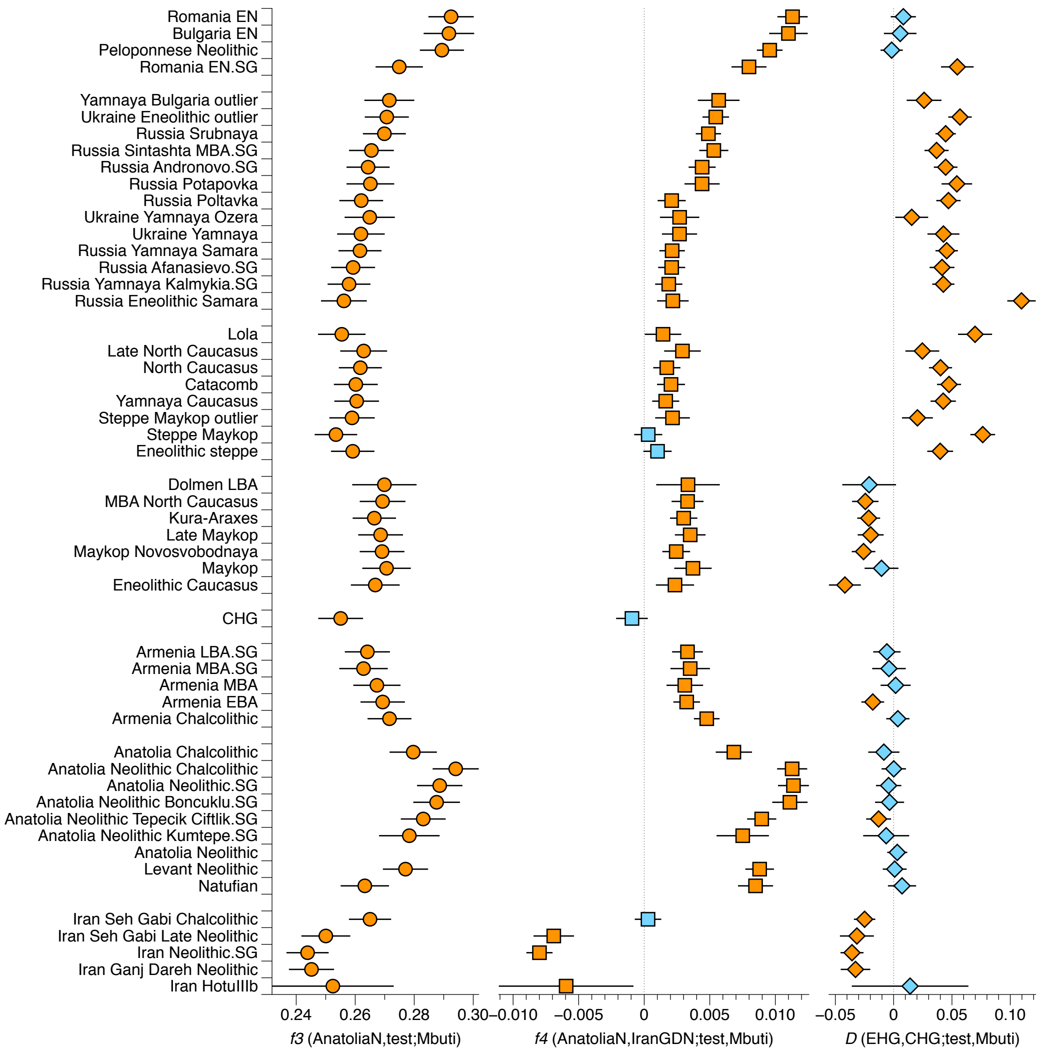

Supplementary Fig. 6. Results of key f_3-, f_4-, and *D*-statistics. Here we explore the affinity of test populations (y-axis) to Anatolia Neolithic, Anatolian Neolithic versus Ganj Dareh Iran Neolithic, and the potential attraction to Basal Eurasian. Significant Z-scores (|Z|>3) are highlighted in orange, and *f*-/*D*-values include three standard errors.

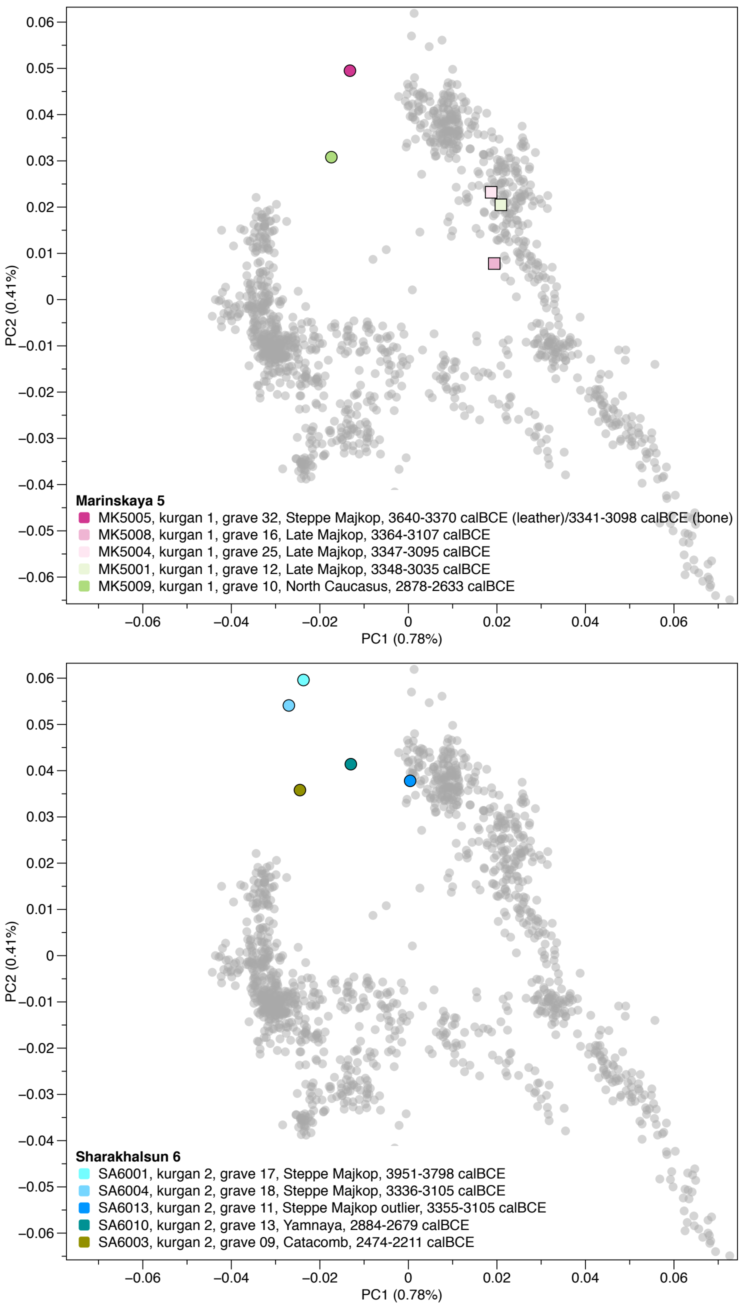

Supplementary Fig. 7. PCA results from two selected kurgan sites.
Here we highlight the genetic heterogeneity of multiple individuals from the same kurgan sites: Marinskaya 5 (upper panel) and Sharakhalsun 6 (lower panel).

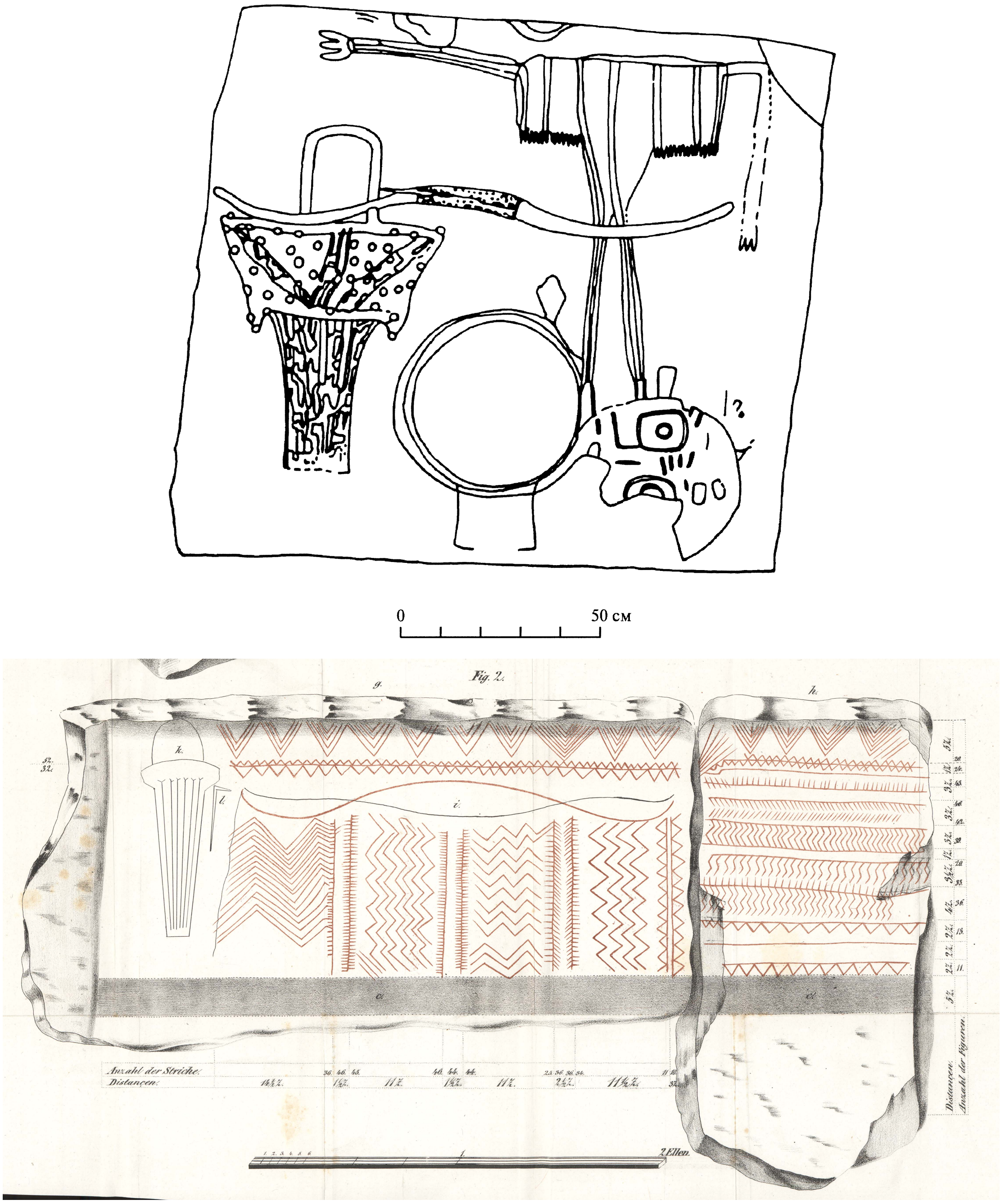

Supplementary Fig. 8. Similarities in weaponry and burial rites.
Depictions in a grave chamber of the Novosvobodnaya Klady group (Kurgan 28, grave 1) near Maykop (panel above; from reference[1]) and the Middle Neolithic Bernburg culture site Göhlitzsch in Germany (panel below; from reference[2], no rights reserved). Both date to the late 4th millennium BCE and display a recurve bow and quiver sets as examples of innovative weaponry.

Supplementary Tables

Supplementary Table 1. Testing genetic affinities in Steppe Maykop individuals. Top significantly positive, selection of non-significant, and top significantly negative Z-scores of admixture f_4-statistics of the form f_4(Mbuti, test; Steppe Maykop, Eneolithic_steppe).

Test	f4	Z	BABA	ABBA	SNPs
Selected significantly negative					
Fuego_Patagonian.SG	-0.003146	-9.993	47848	45041	892414
Karitiana.DG	-0.003037	-9.791	50518	47664	939832
AfontovaGora3	-0.004451	-9.569	13498	12367	253935
Mixe.DG	-0.002652	-8.804	49131	46687	921659
Eskimo_Naukan.DG	-0.002544	-8.649	49001	46656	921618
Surui.DG	-0.002942	-8.555	49436	46724	921566
Clovis.SG	-0.00296	-8.178	50418	47641	938101
Kennewick_WA.SG	-0.002781	-7.458	26419	25029	499834
MA1	-0.002762	-6.952	36625	34760	675385
Saqqaq.SG	-0.002143	-6.255	47600	45657	906702
Tibetan_Samzdong_1500BP.SG	-0.001779	-6.059	48730	47065	935508
Ulchi.DG	-0.001737	-5.957	48072	46472	921588
EHG	-0.001837	-5.801	49400	47729	909579
Han.DG	-0.001465	-5.467	48850	47473	939851
She.DG	-0.001551	-5.417	47855	46425	921518
Japanese.DG	-0.001452	-5.394	47799	46461	921664
Ami.DG	-0.001529	-5.191	47869	46459	921603
Tibetan.DG	-0.001497	-5.159	47719	46340	921615
Selected non-significant					
Kostenki14	-0.000239	-0.695	47023	46806	907597
Ust_Ishim.DG	-0.000289	-0.898	44341	44088	879189
WHG	0.00036	1.233	49171	49512	936220
Selected significantly positive					
CHG	0.00222	7.391	47982	50069	939486
Eneolithic_Caucasus	0.00184	5.486	35804	37086	697041
Anatolia_Neolithic	0.00123	5.445	48316	49468	939542
Balkans_Neolithic	0.00126	5.013	47225	48383	918923
LBK_EN	0.00107	4.813	48488	49491	939682
Iran_Seh_Gabi_Chalcolithic	0.00110	4.355	46147	47132	899143
Globular_Amphora_Ukraine	0.00112	4.318	47324	48354	915910
Czech_Neolithic	0.00126	4.298	44110	45185	855823
Anatolia_Neolithic_Chalcolithic	0.00121	4.178	47743	48862	927215
Levant_N	0.00098	3.642	39953	40721	785439

Supplementary Table 2. Modeling Steppe Maykop individuals as a two-way admixture.

Z-scores of f_4-statistics of the form f_4(Steppe_Maykop, Fitted Steppe_Maykop; Outgroup1, Outgroup2) in modeling Steppe_Maykop as a two-way admixture of (Eneolithic_Steppe+AG3), (Eneolithic_Steppe+Kennewick), and (AG3+Kennewick). The Steppe Maykop individuals could not be explained by any of the above three pairs without outliers.

**Outgroup1**	**Outgroup2**	**Eneolithic_Steppe +AG3**	**Eneolithic_Steppe +Kennewick**	**AG3+ Kennewick**
Mbuti.DG	Ust_Ishim.DG	-0.100	-0.417	1.083
Mbuti.DG	Kostenki14	-0.476	-1.638	0.673
Mbuti.DG	MA1	2.168	-7.388	13.987
Mbuti.DG	Han.DG	-4.679	-1.081	-2.681
Mbuti.DG	Papuan.DG	-0.593	-0.413	1.138
Mbuti.DG	Onge.DG	-1.849	-1.336	0.451
Mbuti.DG	Villabruna	-0.365	-1.148	-0.871
Mbuti.DG	Vestonice16	-0.987	-2.456	0.737
Mbuti.DG	ElMiron	0.964	-0.247	0.962
Mbuti.DG	Ethiopia_4500BP.SG	-0.319	-0.002	-0.355
Mbuti.DG	Karitiana.DG	-4.032	-2.605	4.163
Mbuti.DG	Natufian	-0.232	1.040	-2.735
Mbuti.DG	Iran_Ganj_Dareh_Neolithic	-0.165	-0.161	-1.984

Supplementary Table 3. Results of *qpAdm* modelling of Steppe Maykop and Steppe Maykop outlier individuals.

**Modelling Steppe Maykop**<br>left populations: Steppe Maykop, Eneolithic_steppe, AfontovaGora3, Kennewick;						
right populations: Mbuti.DG, Ust_Ishim.DG, Kostenki14, Han.DG, Papuan.DG, Onge.DG, Villabruna, Vestonice16, ElMiron, Ethiopia_4500BP.SG, Karitiana.DG, Natufian, Iran_Ganj_Dareh_Neolithic						
Rank 2	Eneolithic_steppe	std	AG3	std	Kennewick	std
**0.370944611**	0.635	0.029	0.296	0.034	0.069	0.010
**Modelling Steppe Maykop outliers**<br>left populations: Steppe Maykop outliers, Steppe Maykop, test						
right populations: Mbuti.DG, Ust_Ishim.DG, Kostenki14, MA1, Han.DG, Papuan.DG, Onge.DG, Villabruna, Vestonice16, ElMiron, Ethiopia_4500BP.SG, Karitiana.DG, Natufian, Iran_Ganj_Dareh_Neolithic						
Rank 1	Steppe Maykop	std	Maykop	std		
**0.056110048**	0.292	0.038	0.708	0.038		
Rank 1	Steppe Maykop	std	MBA North Caucasus	std		
**0.0884233388**	0.354	0.037	0.646	0.037		
Rank 1	Steppe Maykop	std	Dolmen LBA	std		
**0.428983881**	0.131	0.029	0.869	0.029		
Rank 1	Steppe Maykop	std	Kura-Araxes	std		
**0.469927787**	0.361	0.036	0.639	0.036		
Rank 1	Steppe Maykop	std	Maykop Novosvobodnaya	std		
0.0328524599	0.386	0.035	0.614	0.035		
Rank 1	Steppe Maykop	std	Eneolithic_Caucasus	std		
0.0178596352	0.363	0.042	0.637	0.042		
Rank 1	Steppe Maykop	std	Armenia_EBA	std		
0.0240315342	0.377	0.036	0.623	0.036		
Rank 1	Steppe Maykop	std	Armenia_Chalcolithic	std		
0.000179325248	0.345	0.040	0.655	0.040		
Rank 1	Steppe Maykop	std	Armenia_LBA	std		
0.0188853002	0.235	0.042	0.765	0.042		
Rank 1	Steppe Maykop	std	Anatolia_Chalcolithic	std		
7.2808967e-06	0.422	0.039	0.578	0.039		

Supplementary Table 4. Exploring genetic affinities of the *Caucasus* cluster.
Top 10 negative Z-scores of admixture f_3-statistics of the form f_3(source1, source2, target) exploring possible sources for the five Caucasus groups as targets.

Source 1	Source 2	Target	f_3	Z
Romania EN	CHG	Maykop Novosvobodnaya	-0.00942	-5.914
LBK Hungary MN	CHG	Maykop Novosvobodnaya	-0.00995	-5.424
ALPc MN	CHG	Maykop Novosvobodnaya	-0.00728	-5.145
Bulgaria Late Chalcolithic	CHG	Maykop Novosvobodnaya	-0.00662	-4.585
Levant N	CHG	Maykop Novosvobodnaya	-0.00724	-4.580
ALPc III MN	CHG	Maykop Novosvobodnaya	-0.00727	-4.304
Anatolia Neolithic	CHG	Maykop Novosvobodnaya	-0.00544	-4.299
LBK EN	CHG	Maykop Novosvobodnaya	-0.00532	-4.291
SopotLN	CHG	Maykop Novosvobodnaya	-0.00648	-4.218
ALPc Tiszadob MN	CHG	Maykop Novosvobodnaya	-0.00641	-4.201
EN TRB	CHG	Maykop	-0.01914	-4.486
Levant N	Eneolithic steppe	Maykop	-0.0122	-4.198
Levant N	CHG	Maykop	-0.013	-4.060
ALPc Tiszadob MN	CHG	Maykop	-0.01223	-4.041
Starcevo EN	Ganj Dareh Neolithic	Maykop	-0.01195	-3.928
Romania EN	CHG	Maykop	-0.01192	-3.750
Anatolia Neolithic	CHG	Maykop	-0.00979	-3.720
Sopot LN	CHG	Maykop	-0.01186	-3.711
Anatolia Neolithic	Ganj Dareh Neolithic	Maykop	-0.01065	-3.692
LBK_EN	CHG	Maykop	0.009672	-3.656
Romania EN	CHG	Late_Maykop	-0.01198	-5.893
ALPc MN	CHG	Late_Maykop	-0.01033	-5.574
Balkans Neolithic	Afontova Gora 3	Late_Maykop	-0.01358	-5.475
Afontova Gora 3	Croatia Cardial Neolithic	Late_Maykop	-0.01364	-5.249
Levant N	CHG	Late_Maykop	-0.01049	-5.195
LBK EN	Afontova Gora 3	Late_Maykop	-0.01179	-5.099
Protoboleraz LCA	CHG	Late_Maykop	-0.00925	-5.049
Anatolia Neolithic	Afontova Gora 3	Late_Maykop	-0.01147	-4.901
ALPc Szatmar MN	CHG	Late_Maykop	-0.01018	-4.832
LBK_Hungary MN	CHG	Late_Maykop	-0.01051	-4.715
Levant_N	CHG	Kura_Araxes	-0.01026	-6.618
ALPc_MN	CHG	Kura_Araxes	-0.00851	-5.672
Romania_EN	CHG	Kura_Araxes	-0.00914	-5.573
LBK_Hungary_MN	CHG	Kura_Araxes	-0.01097	-5.502
LBK_EN	CHG	Kura_Araxes	-0.00731	-5.242
Croatia Cardial Neolithic	CHG	Kura_Araxes	-0.00791	-5.068
Bulgaria Late Chalcolithic	CHG	Kura_Araxes	-0.00765	-5.045
Anatolia_Neolithic	CHG	Kura_Araxes	-0.00696	-5.012
Bulgaria_EN	CHG	Kura_Araxes	-0.01097	-5.000
LBKT_MN	CHG	Kura_Araxes	-0.00752	-4.995
LBK_Hungary_MN	CHG	MBA_North_Caucasus	-0.0059	-2.562
Koros_EN_all	CHG	MBA_North_Caucasus	-0.00543	-1.777
Romania_EN	CHG	MBA_North_Caucasus	-0.0038	-1.770
Bulgaria_Neolithic	CHG	MBA_North_Caucasus	-0.0033	-1.493
ALPc_III_MN	CHG	MBA_North_Caucasus	-0.00322	-1.479
ALPc_Szatmar_MN	CHG	MBA_North_Caucasus	-0.00318	-1.439
ALPc_MN	CHG	MBA_North_Caucasus	-0.00282	-1.409
Levant_N	CHG	MBA_North_Caucasus	-0.0029	-1.331
Bulgaria Late Chalcolithic	CHG	MBA_North_Caucasus	-0.00245	-1.194
Minoan_Odigitria	CHG	MBA_North_Caucasus	-0.00326	-1.151

Supplementary Table 5. *qpWave* results.

P-values for the number of streams of ancestry in the Caucasus cluster. We used the following populations as outgroups: Mbuti.DG, Ust_Ishim.DG, Kostenki14, MA1, Han.DG, Papuan.DG, Onge.DG, Villabruna, Vestonice16, ElMiron, Ethiopia_4500BP.SG, Karitiana.DG, Natufian, Iran_Ganj_Dareh_Neolithic. Since the *qpWave* results can be sensitive with the option 'allsnps: YES' to which population is first in the list of testing populations, we circulated the first population in the testing groups to check if the patterns we observe were robust or not.

**First population used in the test**	**P rank 0**	**P rank 1**
Eneolithic Caucasus	**0.144**	**0.435**
Kura-Araxes	0.050	**0.401**
Late Maykop	0.017	**0.135**
Maykop	0.009	**0.322**
MBA North Caucasus	0.042	**0.220**
Maykop Novosvobodnaya	0.005	**0.111**
Dolmen LBA	**0.173**	**0.499**

Supplementary Table 6. *qpWave* results of the *Caucasus* cluster.
P values of rank=1 in modelling the two-way admixture in the Caucasus cluster. Right populations: Mbuti.DG, Ust_Ishim.DG, Kostenki14, MA1, Han.DG, Papuan.DG, Onge.DG, Villabruna, Vestonice16, ElMiron, Ethiopia_4500BP.SG, Karitiana.DG, Natufian, Iran_Ganj_Dareh_Neolithic. Source 2 populations in bold print are used as examples in modelling the Caucasus cluster groups (See Supplementary Table 7).

Source 1	Source 2	N	SNPs	Eneolithic_ Caucasus	Maykop_ Novosvo bodnaya	Maykop	Late_Maykop	Kura_Araxes	MBA_North_ Caucasus	Dolmen_LBA
CHG	Bulgaria_Middle_Chalcolithic	1	715613	4.463E-01	3.989E-03	3.135E-01	1.046E-01	2.720E-02	2.129E-01	8.096E-01
CHG	Balkans_Chalcolithic	2	903919	7.558E-01	2.996E-03	4.493E-01	1.054E-01	3.995E-02	6.595E-01	9.307E-01
CHG	Koros_EN	4	1000773	2.684E-01	5.594E-03	5.423E-02	6.603E-02	2.144E-02	2.848E-01	5.619E-01
CHG	Anatolia_Neolithic	23	1121778	4.780E-01	1.148E-02	6.945E-02	3.779E-02	3.313E-02	3.448E-01	6.722E-01
CHG	Armenia_Chalcolithic	5	1020803	9.066E-02	2.117E-07	6.378E-02	9.463E-02	3.661E-03	5.030E-02	3.048E-01
CHG	Balkans_Neolithic	6	1042954	3.578E-01	3.324E-03	7.240E-02	8.566E-02	1.542E-02	3.890E-01	7.302E-01
CHG	Bulgaria_EN	2	406657	7.086E-01	1.268E-01	1.964E-01	3.698E-02	8.135E-02	4.010E-01	7.816E-01
CHG	Sopot_LN	7	842134	3.026E-01	3.518E-03	1.263E-01	1.116E-01	2.166E-03	3.130E-01	9.208E-01
CHG	Vinca_MN	6	990389	2.883E-01	3.329E-03	2.370E-01	6.403E-02	1.032E-02	2.542E-01	7.933E-01
CHG	Anatolia_Neolithic_Kumtepe.SG	2	135444	5.023E-01	1.463E-02	7.300E-02	7.741E-02	2.907E-01	6.254E-02	8.497E-01
CHG	Starcevo_EN_all	2	913925	3.248E-01	3.410E-02	2.673E-01	1.782E-01	7.372E-03	5.537E-01	6.404E-01
CHG	**Romania_EN**	2	843151	2.910E-01	1.507E-02	1.704E-01	8.744E-02	3.943E-02	4.232E-01	5.385E-01
CHG	Serbia_Neolithic	3	249048	1.872E-01	3.832E-02	5.007E-01	1.982E-01	3.814E-02	2.702E-01	3.584E-01
CHG	Peloponnese_Neolithic	6	1064931	5.327E-01	1.925E-02	6.822E-02	5.213E-02	1.842E-02	3.064E-01	6.920E-01
CHG	Balkans Krepost Neolithic	1	232378	4.995E-01	4.308E-01	4.759E-01	3.366E-01	1.502E-01	9.359E-01	2.469E-01
CHG	Bulgaria_Neolithic	2	816142	5.544E-01	1.409E-01	8.406E-02	1.692E-02	2.311E-02	1.408E-01	5.591E-01
CHG	**Anatolia_Chalcolithic**	1	954187	5.287E-01	6.204E-02	5.527E-01	6.198E-01	3.679E-01	5.297E-01	4.739E-01

Supplementary Table 7. *qpAdm* results of the *Caucasus* cluster.

P values of rank=1 and admixture coefficient of modelling the two-way admixture of the CHG to Anatolian Chalcolithic cline using 14 outgroups: Mbuti.DG, Ust_Ishim.DG, Kostenki14, MA1, Han.DG, Papuan.DG, Onge.DG, Villabruna, Vestonice16, ElMiron, Ethiopia_4500BP.SG, Karitiana.DG, Natufian, Iran_Ganj_Dareh_Neolithic.

Caucasus cluster	p	CHG	Anatolia_ChL	Std. Error
Eneolithic Caucasus	5.287E-01	0.478	0.522	0.080
Maykop Novosvobodnaya	6.204E-02	0.411	0.589	0.062
Maykop	5.527E-01	0.284	0.716	0.081
Late Maykop	6.198E-01	0.433	0.567	0.054
Kura-Araxes	3.679E-01	0.427	0.573	0.054
MBA North Caucasus	5.297E-01	0.458	0.542	0.056
Dolmen LBA	4.739E-01	0.600	0.400	0.145
	**p**	**CHG**	**Romania_EN**	**Std. Error**
Eneolithic Caucasus	2.910E-01	0.674	0.326	0.049
Maykop Novosvobodnaya	1.507E-02	0.621	0.379	0.035
Maykop	1.704E-01	0.545	0.455	0.045
Late Maykop	8.744E-02	0.632	0.368	0.036
Kura-Araxes	3.943E-02	0.645	0.355	0.033
MBA North Caucasus	4.232E-01	0.642	0.358	0.036
Dolmen LBA	5.385E-01	0.699	0.301	0.093
	**p**	**CHG**	**Bulgaria_Neolithic**	**Std. Error**
Eneolithic Caucasus	5.544E-01	0.653	0.347	0.050
Maykop Novosvobodnaya	1.409E-01	0.597	0.403	0.034
Maykop	8.406E-02	0.510	0.490	0.049
Late Maykop	1.692E-02	0.634	0.366	0.037
Kura-Araxes	2.311E-02	0.634	0.366	0.036
MBA North Caucasus	1.408E-01	0.634	0.366	0.038
Dolmen LBA	5.591E-01	0.682	0.318	0.098

Supplementary Table 8. ***qpWave*** **results of the** ***Caucasus*** **cluster.**

P values of rank=1 and admixture coefficient of modelling the Caucasus cluster as a two-way admixture of the Iran_Ganj_Dareh_Neolithic or EHG with another population using 13 outgroups: Mbuti.DG, Ust_Ishim.DG, Kostenki14, MA1, Han.DG, Papuan.DG, Onge.DG, Villabruna, Vestonice16, ElMiron, Ethiopia_4500BP.SG, Karitiana.DG, Natufian. We tested every available population in our dataset. For Iran_Ganj_Dareh_Neolithic, we only show the two fitted pairs of using Armenia Chalcolithic or Anatolia Chalcolithic as the other source. For EHG, we only give the results of using Iran_Seh_Gabi_Chalcolithic as the other source since the Iran_Seh_Gabi_Chalcolithic samples are plotted at the end of EHG and Caucasus cluster cline in the PCA.

**Source 1**	**Source 2**	**N**	**SNPs**	**Eneolithic Caucasus**	**Maykop_ Novosvo bodnaya**	**Maykop**	**Late Maykop**	**Kura_Araxes**	**MBA_North Caucasus**	**Dolmen LBA**
Iran_Ganj_Dareh Neolithic	Armenia Chalcolithic	5	1020803	4.359E-01	4.808E-02	2.043E-01	1.663E-01	5.215E-02	1.629E-01	1.293E-01
Iran_Ganj_Dareh Neolithic	Anatolia Chalcolithic	1	954187	2.834E-01	4.844E-01	3.458E-01	6.253E-02	2.697E-01	5.278E-02	7.337E-02
EHG	Iran_Seh_Gabi Chalcolithic	5	1013671	7.474E-03	8.763E-11	6.903E-06	1.413E-07	6.728E-09	1.396E-06	9.084E-02

Supplementary Table 9. ***qpWave*** **results of the** ***Caucasus*** **cluster with three source populations.**
P values of rank=2 and ancestry proportions in modelling a three-way admixture in the Caucasus cluster testing additional contribution from EHG and WHG, respectively. Right populations: Mbuti.DG, Ust_Ishim.DG, Kostenki14, MA1, Han.DG, Papuan.DG, Onge.DG, Villabruna, Vestonice16, ElMiron, Ethiopia_4500BP.SG, Karitiana.DG, Natufian, Iran_Ganj_Dareh_Neolithic

**Caucasus cluster**	**Eneolithic Caucasus**	**Maykop_ Novosvobodnaya**	**Maykop**	**Late Maykop**	**Kura-Araxes**	**MBA North Caucasus**	**Dolmen LBA**
Left populations: X, EHG, CHG, Anatolia_ChL							
p of rank2	4.623E-01	1.794E-01	5.273E-01	5.924E-01	3.333E-01	4.479E-01	4.097E-01
**EHG**	-0.013	-0.053	0.028	0.022	-0.018	-0.004	0.028
std	0.031	0.024	0.032	0.026	0.025	0.026	0.057
**CHG**	0.484	0.438	0.275	0.423	0.434	0.459	0.586
std	0.081	0.059	0.081	0.054	0.055	0.057	0.145
**Anatolia_ChL**	0.529	0.615	0.697	0.555	0.583	0.545	0.386
std	0.082	0.060	0.082	0.055	0.056	0.058	0.147
Left populations: X, EHG, CHG, Romania_EN							
p of rank2	2.307E-01	1.539E-02	2.217E-01	1.253E-01	4.267E-02	3.627E-01	5.135E-01
**EHG**	0.009	-0.026	0.046	0.041	0.003	0.013	0.046
std	0.031	0.022	0.030	0.025	0.022	0.025	0.055
**CHG**	0.666	0.638	0.512	0.598	0.635	0.633	0.659
std	0.057	0.038	0.048	0.040	0.036	0.039	0.103
**Romania_EN**	0.325	0.387	0.442	0.361	0.362	0.354	0.294
std	0.049	0.035	0.045	0.035	0.032	0.036	0.092
Left populations: X, EHG, CHG, Bulgaria_Neolithic							
p of rank2	5.246E-01	1.019E-01	1.629E-01	7.049E-02	2.005E-02	1.498E-01	5.900E-01
**EHG**	0.024	0.000	0.058	0.063	0.023	0.030	0.062
std	0.029	0.021	0.030	0.025	0.023	0.024	0.053
**CHG**	0.627	0.597	0.469	0.580	0.615	0.611	0.621
std	0.058	0.039	0.052	0.041	0.040	0.041	0.109
**Bulgaria_Neolithic**	0.348	0.403	0.473	0.357	0.362	0.359	0.317
std	0.050	0.034	0.049	0.035	0.036	0.037	0.095

Supplementary Table 9, continued.
P values of rank=2 and ancestry proportions in modelling a three-way admixture in the Caucasus cluster testing additional contribution from EHG and WHG, respectively. Right populations: Mbuti.DG, Ust_Ishim.DG, Kostenki14, MA1, Han.DG, Papuan.DG, Onge.DG, Villabruna, Vestonice16, ElMiron, Ethiopia_4500BP.SG, Karitiana.DG, Natufian, Iran_Ganj_Dareh_Neolithic

**Caucasus cluster**	**Eneolithic Caucasus**	**Maykop_ Novosvobodnaya**	**Maykop**	**Late Maykop**	**Kura-Araxes**	**MBA North Caucasus**	**Dolmen LBA**
Left populations: X, WHG, CHG, Anatolia_ChL							
p of rank2	4.450E-01	5.717E-02	5.121E-01	5.291E-01	4.077E-01	4.387E-01	6.323E-01
**WHG**	0.007	-0.016	0.016	-0.002	-0.020	-0.002	0.063
std	0.019	0.017	0.021	0.017	0.017	0.017	0.039
**CHG**	0.481	0.388	0.304	0.430	0.402	0.453	0.677
std	0.083	0.069	0.085	0.059	0.060	0.060	0.147
**Anatolia_ChL**	0.512	0.628	0.679	0.572	0.618	0.549	0.260
std	0.091	0.078	0.095	0.067	0.069	0.068	0.169
Left populations: X, WHG, CHG, Romania_EN							
p of rank2	2.223E-01	1.496E-02	1.311E-01	6.804E-02	8.928E-02	3.548E-01	6.740E-01
**WHG**	0.001	-0.018	0.010	-0.010	-0.023	-0.008	0.058
std	0.019	0.015	0.020	0.016	0.015	0.016	0.038
**CHG**	0.673	0.606	0.549	0.626	0.621	0.637	0.728
std	0.050	0.037	0.046	0.036	0.033	0.037	0.096
**Romania_EN**	0.326	0.412	0.441	0.384	0.402	0.371	0.214
std	0.057	0.045	0.055	0.043	0.040	0.044	0.113
Left populations: X, WHG, CHG, Bulgaria_Neolithic							
p of rank2	4.592E-01	1.146E-01	5.754E-02	1.018E-02	2.372E-02	9.731E-02	7.038E-01
**WHG**	0.003	-0.010	0.004	0.002	-0.018	0.002	0.707
std	0.019	-0.010	0.004	0.016	0.015	0.016	0.101
**CHG**	0.651	0.591	0.512	0.633	0.621	0.634	0.236
std	0.051	0.590	0.512	0.038	0.038	0.039	0.118
**Bulgaria_Neolithic**	0.346	0.420	0.483	0.365	0.397	0.365	0.057
std	0.057	0.420	0.483	0.044	0.045	0.045	0.037

Supplementary Table 10. *qpAdm* results of the Caucasus cluster with three proximal source populations.
P values of rank=2 and ancestry proportions in modelling a three-way admixture in the Caucasus cluster testing additional contribution from Iran_ChL. Here, we used an extended set of outgroup populations populations to constrain standard errors: Right populations: Mbuti.DG, Ust_Ishim.DG, Kostenki14, MA1, Han.DG, Papuan.DG, Onge.DG, Villabruna, Vestonice16, ElMiron, Ethiopia_4500BP.SG, Karitiana.DG, Natufian, Iran_Ganj_Dareh_Neolithic, EHG, WHG, Levant_N.

**Caucasus cluster**	**Eneolithic Caucasus**	**Maykop Novosvobodnaya**	**Maykop**	**Late Maykop**	**Kura-Araxes**	**MBA North Caucasus**	**Dolmen LBA**
Left populations: X, Iran_ChL, CHG, Anatolia_ChL							
P of rank 2	4.561E-01	2.162E-01	6.011E-01	4.606E-01	2.485E-01	5.038E-01	6.903E-01
**CHG**	0.469	0.113	0.313	0.307	0.221	0.310	0.929
std	0.131	0.085	0.137	0.091	0.083	0.086	0.353
**Anatolia_ChL**	0.563	0.512	0.775	0.576	0.530	0.552	0.673
std	0.077	0.054	0.087	0.056	0.054	0.057	0.188
**Iran_ChL**	-0.033	**0.374**	-0.088	0.117	**0.249**	0.137	-0.602
std	0.168	**0.112**	0.184	0.115	**0.109**	0.112	0.457
Left populations: X, Iran_ChL, CHG, Romania_EN							
P of rank 2	6.800E-02	6.426E-03	2.745E-02	5.533E-02	1.177E-02	2.923E-01	7.235E-01
**CHG**	0.760	0.364	0.783	0.589	0.461	0.592	1.165
std	0.159	0.116	0.198	0.111	0.099	0.089	0.391
**Romania_EN**	0.363	0.341	0.521	0.388	0.333	0.369	0.527
std	0.059	0.038	0.072	0.043	0.036	0.038	0.148
**Iran_ChL**	-0.122	0.294	-0.305	0.022	0.205	0.040	-0.692
std	0.202	0.145	0.257	0.143	0.125	0.114	0.494
Left populations: X, Iran_ChL, CHG, Bulgaria_Neolithic							
P of rank 2	4.757E-01	1.139E-01	1.204E-01	5.356E-02	1.909E-02	1.264E-01	7.282E-01
**CHG**	0.671	0.485	0.724	0.934	0.723	0.731	0.938
std	0.219	0.168	0.317	0.259	0.258	0.220	0.467
**Bulgaria_Neolithic**	0.352	0.383	0.538	0.421	0.381	0.379	0.392
std	0.064	0.044	0.080	0.061	0.054	0.049	0.147
**Iran_ChL**	-0.022	0.132	-0.262	-0.355	-0.105	-0.111	-0.331
std	0.252	0.195	0.370	0.298	0.292	0.245	0.536

Supplementary Table 11. Exploring genetic affinities of the *Steppe* cluster.
Top 5 negative Z-scores of admixture f_3-statistics of the form f_3(source1, source2; target) with each Yamnaya related group as a target.

Source 1	Source 2	Target	f3	std.err	Z	SNPs
EHG	CHG	Yamnaya_Samara	-0.010274	0.001084	-9.478	513981
LBK_EN	AfontovaGora3	Yamnaya_Samara	-0.011663	0.001259	-9.266	137163
Balkans_Neolithic	AfontovaGora3	Yamnaya_Samara	-0.012884	0.001497	-8.608	133505
CHG	Latvia_MN	Yamnaya_Samara	-0.008208	0.000968	-8.482	493342
Anatolia_Neolithic	AfontovaGora3	Yamnaya_Samara	-0.010862	0.001294	-8.394	136949
CHG	Ukraine_Mesolithic	Yamnaya_Caucasus	-0.012215	0.001472	-8.298	223469
Balkans_Neolithic	AfontovaGora3	Yamnaya_Caucasus	-0.01659	0.002045	-8.113	73963
CHG	Latvia_MN	Yamnaya_Caucasus	-0.011125	0.001402	-7.937	231067
LBK_EN	AfontovaGora3	Yamnaya_Caucasus	-0.014556	0.001891	-7.698	77689
EHG	CHG	Yamnaya_Caucasus	-0.011461	0.001508	-7.6	218839
England_EMBA	Yamnaya_Caucasus	Yamnaya_Ukraine	-0.015492	0.004144	-3.739	15112
Iran_Ganj_Dareh_Neolithic	Ukraine_Mesolithic	Yamnaya_Ukraine	-0.016117	0.004712	-3.42	14584
England_EMBA	Lola	Yamnaya_Ukraine	-0.017002	0.005048	-3.368	12925
Scotland_LBA	Yamnaya_Caucasus	Yamnaya_Ukraine	-0.013133	0.003915	-3.354	17096
Minoan_Lasithi.SG	Yamnaya_Caucasus	Yamnaya_Ukraine	-0.015539	0.004797	-3.24	12385
Iberia_EN.SG	AfontovaGora3	Yamnaya_Kalmykia.SG	-0.014014	0.001889	-7.417	97596
EHG	CHG	Yamnaya_Kalmykia.SG	-0.008965	0.001297	-6.912	372544
Balkans_Neolithic	AfontovaGora3	Yamnaya_Kalmykia.SG	-0.012315	0.001798	-6.849	100729
LBK_EN	AfontovaGora3	Yamnaya_Kalmykia.SG	-0.010061	0.001595	-6.31	105635
Iberia_EN	AfontovaGora3	Yamnaya_Kalmykia.SG	-0.010914	0.001817	-6.006	99366
Iberia_EN.SG	AfontovaGora3	Afanasievo.SG	-0.02245	0.00584	-3.844	8207
Latvia_MN_Comb_Ware.SG	Balkans_Dzhulyunitsa_Neolithic	Afanasievo.SG	-0.030922	0.008325	-3.714	4672
Wales_MN	Lola	Afanasievo.SG	-0.018829	0.005163	-3.647	13840
AfontovaGora3	Hunyadihalom_MCHA	Afanasievo.SG	-0.027359	0.007734	-3.538	5589
Anatolia_Neolithic_Boncuklu.SG	AfontovaGora3	Afanasievo.SG	-0.022865	0.006583	-3.474	7477
Bulgaria_Varna_Eneolithic2	Latvia_MN_Comb_Ware.SG	Potapovka	-0.007181	0.005638	-1.274	11545
ALPc_I_MN	Latvia_MN_Comb_Ware.SG	Potapovka	-0.008502	0.007176	-1.185	7861
Bulgaria_Varna_Eneolithic2	AfontovaGora3	Potapovka	-0.008672	0.007677	-1.13	6639
Latvia_MN_Comb_Ware.SG	Balkans_Dzhulyunitsa_Neolithic	Potapovka	-0.007744	0.007686	-1.008	6794
Esperstedt_MN	AfontovaGora3	Potapovka	-0.007053	0.007012	-1.006	8163
Balkans_Neolithic	AfontovaGora3	Poltavka	-0.01303	0.001819	-7.165	104933
LBK_EN	AfontovaGora3	Poltavka	-0.011469	0.001605	-7.145	109957
EHG	CHG	Poltavka	-0.010095	0.001429	-7.065	389478
EHG	Maykop_Novosvobodnaya	Poltavka	-0.008737	0.001253	-6.97	322216
Iberia_EN.SG	AfontovaGora3	Poltavka	-0.013091	0.001896	-6.904	101275

Supplementary Table 12. P-values for the number of streams of ancestry in the Yamnaya related steppe ancestry cluster.

We used the following populations as outgroups: Mbuti.DG, Ust_Ishim.DG, Kostenki14, MA1, Han.DG, Papuan.DG, Onge.DG, Villabruna, Vestonice16, ElMiron, Ethiopia_4500BP.SG, Karitiana.DG, Natufian, Iran_Ganj_Dareh_Neolithic. Since the *qpWave* results can be sensitive to which population is first in the list of testing populations with the parameter "allsnps: YES", we circulated the first population in the testing groups to check if the patterns we observe were robust or not. When the 11 Yamnaya related populations are analyzed together, a minimum of 4 streams of ancestry are needed to relate them to the outgroups.

First population used in the test	P rank 0	P rank 1	P rank 2	P rank 3
Yamnaya_Caucasus	9.579E-21	1.596E-07	7.703E-03	3.839E-01
Yamnaya_Ukraine	4.052E-22	7.137E-08	6.353E-04	3.191E-01
Yamnaya_Samara	2.546E-22	6.700E-08	2.466E-03	4.370E-01
Yamnaya_Kalmykia.SG	4.822E-20	2.786E-08	1.873E-03	1.944E-01
Potapovka	1.305E-19	1.695E-07	2.498E-02	7.713E-01
Poltavka	1.800E-21	5.393E-08	2.636E-03	3.960E-01
Afanasievo.SG	1.075E-23	2.768E-08	2.096E-03	2.368E-01
Yamnaya_Ukraine_Ozera	8.050E-15	4.522E-05	6.241E-02	8.339E-01

Supplementary Table 13. *qpAdm* results of the *Steppe* cluster with three source populations.
P values of rank=2 and admixture proportions in modelling Steppe ancestry populations as a three-way admixture of Eneolithic steppe Anatolian_Neolithic and WHG using 14 outgroups.
Left populations: Test, Eneolithic_steppe, Anatolian_Neolithic, WHG
Right populations: Mbuti.DG, Ust_Ishim.DG, Kostenki14, MA1, Han.DG, Papuan.DG, Onge.DG, Villabruna, Vestonice16, ElMiron, Ethiopia_4500BP.SG, Karitiana.DG, Natufian, Iran_Ganj_Dareh_Neolithic.

Test	p	Eneolithic_Steppe		Anatolian_Neolithic		WHG	
		proportion	std.err	proportion	std.err	proportion	std.err
Yamnaya_Caucasus	6.718E-01	0.839	0.028	0.132	0.027	0.030	0.014
Yamnaya_Ukraine	4.202E-01	0.855	0.038	0.091	0.037	0.054	0.021
Yamnaya_Samara	3.806E-01	0.889	0.025	0.052	0.026	0.059	0.013
Yamnaya_Kalmykia.SG	3.154E-04	0.949	0.029	0.034	0.028	0.017	0.015
Potapovka	1.401E-01	0.677	0.037	0.191	0.037	0.132	0.019
Poltavka	7.792E-01	0.923	0.027	0.016	0.026	0.061	0.015
Afanasievo.SG	2.953E-04	0.944	0.030	0.017	0.029	0.039	0.016
Yamnaya_Ukraine_Ozera	5.886E-02	0.675	0.041	0.345	0.042	-0.020	0.021
North_Caucasus	5.175E-02	0.835	0.026	0.127	0.027	0.038	0.013
Late_North_Caucasus	3.759E-01	0.750	0.039	0.246	0.040	0.004	0.020
Catacomb	3.750E-01	0.836	0.026	0.106	0.026	0.057	0.013
Steppe_Maykop	6.607E-08	1.320	0.035	-0.285	0.034	-0.035	0.018
Steppe_Maykop_outlier	4.978E-02	0.840	0.040	0.222	0.042	-0.062	0.019
Lola	5.457E-05	1.128	0.043	-0.125	0.042	-0.003	0.022
Srubnaya	1.773E-03	0.624	0.022	0.234	0.021	0.142	0.011
Sintashta_MBA_RISE.SG	4.651E-07	0.578	0.029	0.286	0.028	0.136	0.015
Andronovo.SG	1.357E-08	0.705	0.028	0.193	0.027	0.102	0.014

Supplementary Table 14. ***qpAdm*** **results of the** ***Steppe*** **cluster with four source populations.**
P values of rank=3 and admixture proportions in modelling Steppe ancestry populations as a four-way admixture of distal sources EHG, CHG, Anatolian_Neolithic and WHG using 14 outgroups.
Left populations: *Steppe* cluster, EHG, CHG, WHG, Anatolian_Neolithic
Right populations: Mbuti.DG, Ust_Ishim.DG, Kostenki14, MA1, Han.DG, Papuan.DG, Onge.DG, Villabruna, Vestonice16, ElMiron, Ethiopia_4500BP.SG, Karitiana.DG, Natufian, Iran_Ganj_Dareh_Neolithic.

**Population**	**p**	**EHG**		**CHG**		**WHG**		**Anatolian_Neolithic**	
		proportion	std.err	proportion	std.err	proportion	std.err	proportion	std.err
Yamnaya_Caucasus	8.424E-02	0.426	0.030	0.443	0.042	0.020	0.018	0.110	0.037
Yamnaya_Ukraine	1.856E-01	0.462	0.039	0.401	0.054	0.028	0.026	0.109	0.048
Yamnaya_Samara	7.185E-02	0.492	0.026	0.394	0.034	0.017	0.015	0.098	0.028
Yamnaya_Kalmykia.SG	3.344E-02	0.504	0.027	0.446	0.035	-0.017	0.018	0.068	0.031
Potapovka	2.182E-01	0.334	0.037	0.348	0.055	0.128	0.025	0.190	0.051
Poltavka	2.789E-02	0.472	0.029	0.475	0.039	0.036	0.018	0.017	0.034
Afanasievo.SG	1.497E-02	0.534	0.030	0.403	0.038	-0.009	0.020	0.072	0.033
Yamnaya_Ukraine_Ozera	1.627E-02	0.280	0.042	0.435	0.061	-0.003	0.027	0.287	0.054
North_Caucasus	2.575E-04	0.407	0.029	0.454	0.039	0.032	0.017	0.107	0.034
Late_North_Caucasus	1.723E-01	0.395	0.039	0.362	0.054	-0.015	0.026	0.258	0.051
Catacomb	2.399E-01	0.444	0.027	0.409	0.038	0.033	0.017	0.115	0.033
Steppe_Maykop	2.902E-01	0.874	0.033	0.341	0.042	-0.159	0.020	-0.056	0.036
Steppe_Maykop_outlier	1.164E-01	0.324	0.040	0.552	0.054	-0.042	0.024	0.166	0.048
Lola	1.628E-02	0.745	0.043	0.322	0.060	-0.105	0.027	0.038	0.053
Srubnaya	5.351E-02	0.349	0.020	0.270	0.028	0.114	0.014	0.267	0.026
Sintashta_MBA_RISE.SG	3.787E-04	0.302	0.028	0.285	0.042	0.117	0.019	0.296	0.038
Andronovo.SG	7.799E-04	0.408	0.026	0.292	0.039	0.064	0.017	0.236	0.036

Supplementary Table 15. *qpAdm* results of the *Steppe* cluster with two proximal source populations.
P values of rank=1 and admixture proportions in modelling Steppe ancestry populations as a two-way admixture of Eneolithic steppe and Anatolian_Neolithic using 14 outgroups.
Left populations: Test, Eneolithic_steppe, Anatolian_Neolithic
Right populations: Mbuti.DG, Ust_Ishim.DG, Kostenki14, MA1, Han.DG, Papuan.DG, Onge.DG, Villabruna, Vestonice16, ElMiron, Ethiopia_4500BP.SG, Karitiana.DG, Natufian, Iran_Ganj_Dareh_Neolithic.

**Test**	**p**	**Eneolithic_Steppe**		**Anatolian_Neolithic**	
		proportion	std.err	proportion	std.err
Yamnaya_Caucasus	3.961E-01	0.859	0.026	0.141	0.026
Yamnaya_Ukraine	1.427E-01	0.890	0.037	0.110	0.037
Yamnaya_Samara	2.362E-03	0.923	0.026	0.077	0.026
Yamnaya_Kalmykia.SG	3.636E-04	0.960	0.028	0.040	0.028
Potapovka	3.064E-09	0.758	0.040	0.242	0.040
Poltavka	2.737E-02	0.964	0.027	0.036	0.027
Afanasievo.SG	5.979E-05	0.968	0.029	0.032	0.029
Yamnaya_Ukraine_Ozera	6.384E-02	0.667	0.040	0.333	0.040
North_Caucasus	7.132E-03	0.851	0.026	0.149	0.026
Late_North_Caucasus	4.618E-01	0.751	0.039	0.249	0.039
Catacomb	3.634E-03	0.869	0.026	0.131	0.026
Steppe_Maykop	2.186E-08	1.303	0.033	-0.303	0.033
Steppe_Maykop_outlier	1.839E-03	0.824	0.040	0.176	0.040
Lola	1.004E-04	1.127	0.041	-0.127	0.041
Srubnaya	1.356E-30	0.746	0.026	0.254	0.026
Sintashta_MBA_RISE.SG	2.327E-21	0.684	0.031	0.316	0.031
Andronovo.SG	5.137E-18	0.784	0.029	0.216	0.029

Supplementary Table 16. Explorative *qpAdm* results of the *Steppe* cluster with two source populations.
P values of rank=1 in modelling Steppe ancestry populations as a two-way admixture of Eneolithic steppe and a population X using 14 outgroups.
Left populations: *Steppe* cluster, Eneolithic_steppe, population X
Right populations: Mbuti.DG, Ust_Ishim.DG, Kostenki14, MA1, Han.DG, Papuan.DG, Onge.DG, Villabruna, Vestonice16, ElMiron, Ethiopia_4500BP.SG, Karitiana.DG, Natufian, Iran_Ganj_Dareh_Neolithic.

Population X	N	SNPs	Yamnaya_ Caucasus	Yamnaya_ Ukraine	Yamnaya_ Samara	Yamnaya_ Kalmykia.SG	Potapovka	Poltavka	Afanasievo.SG	Yamnaya_Ukraine _Ozera
**Bulgaria_Varna_Eneolithic3**	1	405653	8.850E-01	6.316E-01	2.567E-01	2.833E-03	2.033E-01	2.285E-01	6.696E-04	8.474E-05
England_Neolithic_all	3	233464	7.305E-01	5.373E-01	1.738E-01	7.507E-04	9.360E-02	2.540E-01	1.688E-04	2.042E-04
Iberia_Chalcolithic	32	1088316	6.155E-01	4.769E-01	1.668E-01	5.275E-04	5.257E-02	2.546E-01	1.890E-04	2.782E-06
Baalberge_MN	3	524772	8.357E-01	6.012E-01	1.308E-01	6.875E-04	5.288E-02	1.719E-01	1.935E-04	3.300E-04
BenzigerodeHeimburg_LN	2	749021	2.633E-01	3.763E-01	9.482E-02	3.519E-04	1.151E-01	2.519E-01	1.328E-04	3.453E-08
Karsdorf_LN	1	159454	9.889E-02	4.839E-01	4.725E-01	2.269E-01	8.014E-01	2.288E-01	1.476E-01	7.947E-02
Salzmuende_MN	3	138191	9.187E-01	4.918E-01	1.464E-01	1.160E-03	5.661E-02	1.587E-01	7.384E-04	1.199E-03
Iberia_MN	4	957055	6.715E-01	4.567E-01	1.698E-01	5.571E-04	5.389E-02	2.447E-01	1.753E-04	1.626E-06
Alberstedt_LN	1	1035387	4.885E-01	5.432E-01	3.262E-01	3.352E-04	3.134E-01	2.464E-01	1.476E-04	7.809E-06
**Ukraine_Eneolithic**	4	887406	9.668E-02	5.040E-01	5.962E-01	3.951E-04	7.746E-02	7.525E-01	5.670E-04	3.458E-10
France_Neolithic	1	59360	6.661E-01	3.803E-01	4.468E-02	2.756E-04	1.474E-01	1.519E-01	4.820E-05	5.838E-02
Wales_Late_Neolithic	1	238946	7.539E-01	5.822E-01	7.565E-02	7.979E-04	2.568E-01	1.437E-01	2.201E-04	4.019E-04
Baltic_LN	5	1126199	9.735E-02	6.852E-01	6.181E-01	1.795E-03	8.039E-01	7.129E-01	7.322E-04	2.118E-10
Sweden_LN_Olsund	1	682911	1.226E-01	2.343E-01	9.548E-02	2.040E-04	1.366E-01	3.041E-01	6.989E-05	6.998E-06
Globular_Amphora	6	748890	6.126E-01	5.533E-01	1.449E-01	6.051E-04	8.325E-02	2.548E-01	2.093E-04	2.402E-05
Globular_Amphora_Ukraine	3	1024461	4.593E-01	4.585E-01	2.082E-01	8.119E-04	5.177E-02	2.410E-01	2.905E-04	4.342E-06

Supplementary Table 16, continued. Explorative *qpAdm* results of the *Steppe* cluster with two source populations.
P values of rank=1 in modelling Steppe ancestry populations as a two-way admixture of Eneolithic steppe and a population X using 14 outgroups.
Left populations: *Steppe* cluster, *continued*, Eneolithic_steppe, population X
Right populations: Mbuti.DG, Ust_Ishim.DG, Kostenki14, MA1, Han.DG, Papuan.DG, Onge.DG, Villabruna, Vestonice16, ElMiron, Ethiopia_4500BP.SG, Karitiana.DG, Natufian, Iran_Ganj_Dareh_Neolithic.

Population X	N	SNPs	North_Caucasus	Late_North_Caucasus	Catacomb	Steppe_Maykop	Steppe_Maykop_outlier	Lola
**Bulgaria_Varna_Eneolithic3**	1	405653	1.789E-01	7.101E-02	6.917E-01	3.373E-07	5.395E-06	7.077E-05
England_Neolithic_all	3	233464	1.742E-01	3.015E-02	5.661E-01	1.535E-09	1.039E-05	4.355E-05
Iberia_Chalcolithic	32	1088316	8.310E-02	2.356E-02	4.200E-01	2.906E-09	5.094E-06	3.476E-05
Baalberge_MN	3	524772	1.326E-01	9.844E-02	3.104E-01	6.621E-08	1.826E-05	3.881E-05
BenzigerodeHeimburg_LN	2	749021	1.067E-02	1.281E-03	3.303E-01	5.911E-07	1.880E-06	1.194E-04
Karsdorf_LN	1	159454	3.562E-02	4.250E-02	4.871E-01	1.574E-02	1.830E-02	3.192E-04
Salzmuende_MN	3	138191	6.178E-02	1.202E-01	4.028E-01	6.605E-09	3.994E-05	5.874E-05
Iberia_MN	4	957055	6.810E-02	3.103E-02	2.856E-01	5.796E-09	6.408E-06	3.219E-05
Alberstedt_LN	1	1035387	1.364E-01	3.016E-02	3.205E-01	1.489E-05	2.178E-06	1.447E-04
**Ukraine_Eneolithic**	4	887406	3.609E-03	1.420E-04	1.588E-01	4.347E-13	2.254E-06	1.756E-05
France_Neolithic	1	59360	4.242E-01	7.112E-02	7.424E-02	7.209E-05	1.367E-05	2.227E-05
Wales_Late_Neolithic	1	238946	1.020E-01	2.192E-01	3.093E-01	2.158E-06	2.888E-05	9.039E-05
Baltic_LN	5	1126199	9.350E-03	3.342E-04	5.496E-01	1.112E-07	3.036E-06	5.003E-05
Sweden_LN_Olsund	1	682911	1.298E-02	5.954E-03	3.425E-02	7.375E-04	1.663E-06	6.261E-05
Globular_Amphora	6	748890	1.128E-01	3.448E-02	4.166E-01	8.481E-08	7.567E-06	1.064E-04
Globular_Amphora_Ukraine	3	1024461	3.647E-02	3.773E-02	4.324E-01	6.363E-09	6.739E-06	6.232E-05

Supplementary Table 17. ***qpAdm*** **results of the** ***Steppe*** **cluster with two proximal source populations.**

P values of rank=1 and admixture coefficients of modelling the Steppe ancestry populations as a two-way admixture of the Eneolithic_steppe and Globular_Amphora using 14 outgroups.

Left populations: *Steppe* cluster, Eneolithic_steppe, Globular Amphora

Right populations: Mbuti.DG, Ust_Ishim.DG, Kostenki14, MA1, Han.DG, Papuan.DG, Onge.DG, Villabruna, Vestonice16, ElMiron, Ethiopia_4500BP.SG, Karitiana.DG, Natufian, Iran_Ganj_Dareh_Neolithic.

Steppe **cluster group**	**p**	**Eneolithic_ steppe**	**Globular_ Amphora**	**Std. Error**
Yamnaya_Caucasus	6.126E-01	0.834	0.166	0.029
Yamnaya_Ukraine	5.533E-01	0.834	0.166	0.041
Yamnaya_Samara	1.449E-01	0.868	0.132	0.027
Yamnaya_Kalmykia.SG	6.051E-04	0.940	0.060	0.032
Potapovka	8.325E-02	0.620	0.380	0.039
Poltavka	2.548E-01	0.903	0.097	0.029
Afanasievo.SG	2.093E-04	0.930	0.070	0.033
Yamnaya_Ukraine_Ozera	2.402E-05	0.711	0.289	0.046
North_Caucasus	1.128E-01	0.823	0.177	0.026
Late_North_Caucasus	3.448E-02	0.766	0.234	0.042
Catacomb	4.166E-01	0.815	0.185	0.027
Steppe_Maykop	8.481E-08	1.343	-0.343	0.040
Steppe_Maykop_outlier	7.567E-06	0.918	0.082	0.042
Lola	1.064E-04	1.145	-0.145	0.048

Supplementary Table 18. *qpAdm* results of the *Steppe* cluster with two proximal source populations.
P values of rank=1 and admixture coefficients of modelling the two-way admixture of the Eneolithic_steppe to Iberia_Chalcolithic cline using 14 outgroups.
Left populations: *Steppe* cluster, Eneolithic_steppe, Iberia_Chalcolithic
Right populations: Mbuti.DG, Ust_Ishim.DG, Kostenki14, MA1, Han.DG, Papuan.DG, Onge.DG, Villabruna, Vestonice16, El Miron, Ethiopia_4500BP.SG, Karitiana.DG, Natufian, Iran_Ganj_Dareh_Neolithic.

***Steppe* cluster group**	**p**	**Eneolithic_ steppe**	**Iberia_ Chalcolithic**	**Std. Error**
Yamnaya_Caucasus	6.155E-01	0.849	0.151	0.027
Yamnaya_Ukraine	4.769E-01	0.854	0.146	0.038
Yamnaya_Samara	1.668E-01	0.878	0.122	0.024
Yamnaya_Kalmykia.SG	5.275E-04	0.950	0.050	0.028
Potapovka	5.257E-02	0.658	0.342	0.036
Poltavka	2.546E-01	0.912	0.088	0.027
Afanasievo.SG	1.890E-04	0.939	0.061	0.030
Yamnaya_Ukraine_Ozera	2.782E-06	0.763	0.237	0.040
Steppe_Maykop	2.906E-09	1.300	-0.300	0.034
Steppe_Maykop_outlier	5.094E-06	0.937	0.063	0.037
North_Caucasus	8.310E-02	0.840	0.160	0.024
Late_North_Caucasus	2.356E-02	0.800	0.200	0.037
Catacomb	4.200E-01	0.833	0.167	0.025
Lola	3.476E-05	1.108	-0.108	0.040

Supplementary Table 19. ***qpAdm*** **results of the** ***Steppe*** **cluster with three proximal source populations.**

P values of rank=2 in modelling Steppe ancestry populations as a three-way admixture of Eneolithic steppe and a Neolithic population Globular_Amphora and an Iranian/Caucasus cluster population Eneolithic_Caucasus using 14 outgroups.

Left populations: Test, Eneolithic_steppe, Globular_Amphora, Eneolithic_Caucasus

Right populations: Mbuti.DG, Ust_Ishim.DG, Kostenki14, MA1, Han.DG, Papuan.DG, Onge.DG, Villabruna, Vestonice16, ElMiron, Ethiopia_4500BP.SG, Karitiana.DG, Natufian, Iran_Ganj_Dareh_Neolithic

Steppe cluster group	p	Eneolithic_steppe		Globular_Amphora		Eneolithic_Caucasus	
		proportion	Std. Error	proportion	Std. Error	proportion	Std. Error
Yamnaya_Caucasus	6.874E-01	0.791	0.043	0.141	0.034	0.069	0.051
Yamnaya_Ukraine	5.781E-01	0.883	0.066	0.198	0.053	-0.081	0.084
Yamnaya_Samara	4.354E-01	0.940	0.045	0.174	0.034	-0.115	0.054
Yamnaya_Kalmykia.SG	3.917E-04	0.960	0.050	0.071	0.040	-0.030	0.061
Potapovka	3.147E-01	0.721	0.062	0.457	0.054	-0.178	0.081
Poltavka	5.484E-01	0.967	0.045	0.146	0.039	-0.113	0.057
Afanasievo.SG	6.861E-04	1.007	0.055	0.113	0.042	-0.120	0.067
Yamnaya_Ukraine_Ozera	8.016E-02	0.436	0.069	0.155	0.051	0.408	0.085
North_Caucasus	1.170E-01	0.781	0.045	0.156	0.032	0.063	0.055
Late_North_Caucasus	1.225E-01	0.653	0.063	0.175	0.048	0.172	0.075
Catacomb	3.605E-01	0.830	0.044	0.195	0.035	-0.025	0.057
Steppe_Maykop	1.415E-02	1.576	0.072	-0.191	0.052	-0.385	0.084
Steppe_Maykop_outlier	2.234E-02	0.632	0.079	-0.033	0.051	0.401	0.101
Lola	2.948E-02	1.377	0.087	-0.028	0.060	-0.349	0.100

Supplementary Note 1

Ecological and archaeological background of the Caucasus region

Ecological background

The mountain ranges of the Greater Caucasus and the southern Lesser Caucasus form a bridge linking the mountainous zones of the Near East to Eurasia. The South Caucasus river systems are connected to the upper Euphrates and Tigris drainage, and the mountain passes of the Great Caucasus offer a number of passages to the steppe landscapes to the north. However, the most important ecological demarcation is the interface of mountain and steppe ecozones in the North Caucasian foothills, a mid-altitude zone of hills and narrow plateaus and the slightly hilly piedmont steppe[3]. Intermediate biospheres, known as ecotones, which are micro-environmental patches between larger vegetation zones, are characterized by higher biodiversity and provide fodder-rich landscapes ideal for pastoralism but also for basic agriculture[4]. This demarcation and the correlated ecotones fluctuated considerably during the Bronze Age. For instance, the 6 to 5 ky rapid climate change event placed Maykop sites in a much more steppe-like environment than it is today, while the 4.2 ky event put a halt to the exploitation of the steppe zone for several hundred years due to severe aridisation[5]. The ecological division separates mountain- and steppe-based economies despite the fact that both used biologically highly diverse environments with rich pastures ideal for mobile and semi-sedentary pastoralists, but which are traditionally shared by groups practising such economies. At a local level, the Caucasus is therefore characterized by a large variety of specific economic and social adaptations. These are reflected in a large degree of cultural and linguistic diversity, and complex networks of interaction within and between the Caucasus and the adjacent regions.

Archaeological background

The geographical dichotomy of the mountain and steppe-based economies in the Caucasus is also reflected in the high diversity of archaeological cultural phenomena[6, 7, 8]. Our study includes representatives of all major Bronze Age groups in the North Caucasus. They reflect affiliations with either mountain-associated cultural groups such as the Darkveti-Meshoko Eneolithic (4700-3500 calBCE)[9], the Maykop phenomenon (3900-2900 calBCE)[7], communities of a Northern variant of the Kura-Araxes culture (3600-2500 calBCE)[10], the North Caucasian cultural formation or the mountain groups of the post-Catacomb grave horizon[11], or to large-scale cultural phenomena of the steppe such as the Don-Kaspi Steppe Eneolithic (4300-4100 calBCE)[12], the Yamnaya (3300-2600/2500 calBCE), the Catacomb cultural communities (2800-2200 calBCE)[5] and the post-Catacomb Lola formation (2200-1700 calBCE)[13]. To represent the South Caucasus Kura-Araxes culture[6] (3600-2500 calBCE), we also included individuals from the Kaps site in present-day Armenia. Each of these formations is outlined by particular sets of artefacts, settlement and burial practices, or adaptations to specific environments. However, the chronological overlap of sites within the same territory, regional variations in artefact spectra and a high frequency of elements crossing over blur the delineation of clear cut 'archaeological cultures'. Consequently, the applied terms must in their specific time frames be understood as operational tools rather than social entities that represent uniform and coherent peoples.

The significance of the Caucasus as a key study area is clearly visible in the current debate about large-scale cultural interaction in the 4th and 3rd millennium BCE in Western Eurasia. The impact of the archaeological cultures, which shaped this contact zone between the Near East and Eurasia during the Bronze Age, on the widening of social networks that operated between Mesopotamia, the Eurasian steppe and Europe is, however, still poorly understood[14, 15].

Early Neolithic communities established a sedentary lifestyle with a Near Eastern set of domesticates in the South Caucasus by 6000 calBCE[16]. They developed a sophisticated set of

settlements but kept to the floodplains of the major river drainages. Other Neolithic communities in the West Caucasian lowlands and at the Black Sea coast or in the central mountain valleys remained vague and might reflect local populations that selectively adopted elements of the Neolithic package. Neolithic elements in the Lower Don area north of the Caucasus and dating to the late 7th millennium BCE are disputed[17] but might shed light on a southern component in the development of cultural formations before the actual advent of the Neolithic in the North Caucasus during the mid-5th millennium BCE.

The earliest attested evidence of the Neolithic lifestyle in the North Caucasus, including domesticates and settlement architecture, dates to the mid-5th millennium BCE and is associated with a cultural formation termed Darkveti-Meshoko Eneolithic[9] or 'pearl-ornamented ceramic'. Sites associated with this phenomenon are situated on both flanks of the West Caucasian Mountains and in all probability reflect groups advancing through the mountain passes from the southern to the northern side of the mountains. This cultural phenomenon represents a rather dispersed and flimsy settlement of the mountain zones. Some habitation sites were found under rock shelters or in caves such as the site of Unakozovskaya, but large fortified settlements are present as well. The settlers lived on agriculture, cattle herding and pig-rearing. Complementary to the southern Eneolithic component, a northern component started to expand between 4300 and 4100 calBCE manifested in low burial mounds with inhumations densely packed in bright red ochre. Burial sites of this type, like the investigated sites of Progress and Vonyuchka, are found in the Don-Caspian steppe[12], but they are related to a much larger supra-regional network linking elites of the steppe zone between the Balkans and the Caspian Sea[18]. These groups introduced the so-called kurgan, a specific type of burial monument, which soon spread across the entire steppe zone.

The Maykop 'culture' or cultural formation is the first Early Bronze Age culture in the Caucasus. This enigmatic phenomenon exhibits the first gigantic burial mounds with splendid 'royal' tombs[6, 7] containing exquisite burial gifts in gold, silver and bronze that reveal an entire spectrum of technological innovations in metallurgy (among others). Maykop plays a key role in the transfer of technological innovations and social alterations to the West. An increase in sites, the expansion of territories, the enlargement of burial mounds as well as large groupings of mounds in cemeteries indicate a prospering culture and population growth. The economy seems to have been based on an agro-pastoral to pastoral subsistence with a focus on cattle herding, but detailed economic data are largely missing. Judging by the artefact spectrum, the early phase of this phenomenon appears closely linked to northern Mesopotamia[19]. A specific component of the Maykop phenomenon is found in sites located in the modern steppe zone. We refer to them here as Steppe Maykop (cf. [5]). These burials share elements of mortuary practices and artefacts with piedmont Maykop groups but at the same time reveal Eneolithic and autonomous components from the steppe. Both Maykop-related phenomena develop in parallel. The Maykop individuals in the present study come from a variety of sites related to both Steppe and piedmont and date to an earlier (3900-3500 calBCE) and later chronological phase (3500-2900 calBCE), respectively. A more detailed chronology can be found in the cited literature. Some sites in the steppe-zone might be affected by a radiocarbon reservoir effect[20], but even a correction of 100-150 would still render them contemporaneous to the groups in the piedmont zone. The earlier individuals are labelled 'Maykop', 'Steppe Maykop' and come from sites like Nogir, Klady and Marinskaya 5 in the piedmont and foothill zone, as well as individuals from Aygursky 2 and Sharakhalsun 6 in the steppe zone. The later Maykop phase, labelled 'Late Maykop' or 'Maykop-Novosvobodnaya', is represented by western sites such as Klady or Sinyukha, Baksanyonok and Marinskaya 5 in the central piedmonts, and Ipatovo in the steppe zone.

Technological innovations culminate during the advanced stage of Maykop and include evidence of wheeled transport and related technologies[8], high-quality textiles including wool[21], and a technically advanced metallurgy[22]. Imports such as lapis lazuli and turquois from

Afghanistan showcase the extensive range of the exchange-network the Maykop communities participated in. With the exception of writing, the use of iconographic seals, or monumental domestic architecture, the technical innovations of the 4th millennium BCE seen in the Near East can also be found in the Caucasus.
Contemporaneous with the later Maykop phase, the first Yamnaya complexes are present in the steppe zone shortly after 3300 calBCE. The two Yamnaya individuals from the Rasshevatskiy 1 site establish a framework of dates for the Yamnaya in our study area, while somewhat younger dates have been reported from the Caspian steppe[5]. Yamnaya interments of the South Russian and North Caucasus plain share specific burial practices with other regional variants of this far-reaching supra-regional cultural network. They differ however in the quantity of burial goods they use. Complete or dismantled wooden wagons are frequently found in Yamnaya and related graves. Like those from the Volga region, the North Caucasian steppe complexes belong to the earliest representatives of the Yamnaya phenomenon in the Eurasian steppe. Stable isotope and palynological evidence argue for a mobile lifestyle based on pastoralism for these groups, but with rather limited mobility radii.
The Velikent site at the coast of the Caspian Sea is a representative for a northern variant of the Kura-Araxes culture, although it is considerably different from the primary variant, which warrants its own Early Bronze Age designation (3300-2400/2200 calBCE). The site is over 30 ha in size and is comprised of several settlement and cemetery mounds[10, 23]. Velikent and related sites are important for this study as they represent comprehensive agro-pastoral economies.
The Caucasian Middle Bronze Age is represented by the Catacomb grave formation (2800-2200 calBCE) mostly found in the steppe and by the North Caucasian cultural groups (2800-2400 calBCE) and Catacomb culture in the piedmont zone. Both represent economies that were based on mobile pastoralism. The first is one of several variants of the supra-regional Catacomb grave phenomenon, which split into even smaller regional components in the North Caucasus and the Caspian steppe. With the Catacomb horizon, the number of mounds and burials increases exponentially in the steppe. The North Caucasian formation in the piedmont and mountain zone likewise witness a fragmentation into smaller regional units with a rather heterogeneous artefact spectrum. However, the occupation of all ecological niches including the various steppe zones as well as the high mountains indicates, like in the Catacomb area, a considerable intensification of human activity. In particular, the mountain groups must have intensified mining as well, since burials dating to this period frequently contain metal artefacts, e.g. the complex of Lysogorskaya 6 in this study. After 2400 calBCE, Catacomb complexes first overlap with those of the North Caucasian groups and then seem to replace them.
The climatic deterioration of the 4.2 ky event led to a dissolution of both the Catacomb and the North Caucasian cultural formations, replacing them in the steppe and piedmont steppe by cultures of the post-Catacomb horizon or Late Bronze Age 1 (2200-1700 calBCE)[13]. The number of sites decreases rapidly. By 1700 calBCE the steppe including the zones directly at the foothills of the mountains are abandoned. Interments like those of Rasshevatskiy 4 and Nevinnomyskiy represent some of the late Bronze Age complexes. However, the mountain communities reorganized themselves in a final stage of North Caucasian traditions, as seen in complexes from Kabardinka or Kudachurt. These paved the way for a renewed intensification of a semi-mobile pastoral economy at high altitudes, which peaked as a highly efficient combined mountain agricultural system during the Late Bronze Age 2 (1700-1100/1000 calBCE)[24].

Supplementary Note 2

Archaeological context of sites and newly reported individuals

Relative and absolute dates

We provide dates either as relative dates or in the form of absolute dates, i.e. radiocarbon-dated samples. Relative dates are reported based on archaeological context in the form “5500-5000 BCE”. Radiocarbon dates are given in the format “95.4 % CI calibrated radiocarbon age (non-calibrated radiocarbon years, laboratory number). All dates were calibrated with OxCal 4.2.3 (ref) using IntCal13 as calibration curve. For this study we obtained and reported 49 new radiocarbon dates.

Sites and individuals (in alphabetical order)

After the general site descriptions, we provide short profiles of each ancient individual for whom we produced ancient genome-wide data. Where possible, the attributed archaeological culture is given in italics.

Aygurskiy 2, Russia

N 45.690°, E 43.262°

Excavation ‘Nasledie’ 2000, Stavropol, licence №2000-776 (V. A. Babenko)

The Aygurskiy burial mound cemetery 2 was situated on a promontory overlooking the small river of Aygurki, about 22 km east of the city of Ipatovo. The area is a slightly hilly landscape, today in a desert steppe environment[3]. However, as shown in the vicinity of the Ipatovo mound, vegetation zones might have shifted here with changing climate conditions. The valley belongs to the river Kalaus drainage system. All 37 burial mounds of the cemetery, of which mound 22 was the largest and placed slightly aside the others, were excavated by the local heritage organization ‘Nasledie’ in 1998-2001. The mound itself was slightly oval, had a diameter of 38 m and a maximal height of 1.8 m. It comprised three major mound construction phases of which the first was a complex stone construction dating to the Maykop epoch[25]. The sampled individuals stem from this first mound phase. The first interments in the mound, grave 8 and 8a, were found in a massive stone box covered by a cairn-like stone package. It was surrounded by a large circle of vertical stone slabs, some of which had collapsed to the interior. Graves 9, 15 and 16 were added inside the circle, and some were covered by collapsed stones of the stone circle. The entire constructing was then filled with a layer of topsoil. It was covered by a stone shell and an earthen mound-shell (shell 1). The remaining 12 graves date chiefly to the Middle and Late Bronze Age and are associated with the North Caucasian cultural formation (graves 4, 10), the Catacomb cultural formation (graves 7, 12-12) and a late or post-Catacomb horizon (graves 1, 3, 11, 12). Graves 2, 5 and 6 are uncertain in date. The Maykop individuals were part of a paleoanthropological study[26] and investigated with regards to their radiocarbon and stable isotope composition[27]. Paired radiocarbon dates on human and animal bones, chiefly herbivores, revealed radiocarbon off-sets for some bones (see comments below), but confirm a mid to late Maykop date for all graves. The particularities of inventories and grave construction together with the location of the site in the grass-steppe zone associate these complexes with our *Steppe-Maykop* cultural formation. Two sampled individuals from the Maykop complexes produced genome-wide data.

- AY2003.A0101.TF1 (BZNK-289/1), kurgan 22, grave 9, was the burial of a 1.5-year-old child in an oval burial pit covered by stones and associated with two ceramic vessels, a flint flake and an astragalus[25]. Dating: human bone 3634-3377 calBCE (4726±31 BP, OxA-16147), animal bone 3638-3531 calBCE (4798±33 BP, OxA-16201), showing no radiocarbon offset in diet[27].

- AY2001.A0101.TF1 (BZNK-290/1), kurgan 22, grave 15 of an 55-65-year-old female in a long-oval pit with entrance shaft, partly covered by stones and associated with a ceramic vessel. Dating: human bone 3359-3013 calBCE (4480±50 BP, GIN-11811), 3630-3372 calBCE (4698±32 BP, OxA-16182), animal bone 3619-3378 cal BCE (4608±31 BP, OxA-16183), indicating a reservoir offset of -157 to -97 years. We would suggest that the age of the human is more likely to date to the period indicated by the animal bone dated[27].

Baksanyonok, Russia
N 43.695°, E 43.644°
Excavation 'Institute of Archaeology of the Caucasus', Nalchik
The mound belongs to a number of mid-size and larger mounds in the vicinity of the village of Baksanyonok. These were excavated over the course of several years in various rescue archaeology projects.
One individual produced genome-wide data:

- I1720, kurgan 2, grave 5, a *Maykop* burial from a mid-size burial mound. No further information is available.

Beliy Ugol 2, Russia
N 44.023°, E 42.818°
Excavation 'Nasledie' 2003, Stavropol, licence №2003-95 (V.A. Babenko).
The site of Beliy Ugol 2 is a burial mound cemetery situated in the North Caucasian foothills near the city of Essentuki, directly at the opening of a narrow gorge into the mountain valleys. The site is situated in a forest steppe to forest environment, which belongs to the Caucasian mountain vegetation zones[3]. The largest mound, mound 1, was excavated during rescue excavations, and parts of the burials had been seriously damaged. Before destruction, the mound had a diameter of approximately 40 m and a height of 3.7 m. In the barrow, 37 burials were excavated. 31 of the burials date to the North Caucasian cultural formation, but the complex stratigraphy suggests at least three major building phases that can be subdivided further. Grave 11 is the central grave of the fourth mound-shell, yet probably not the earliest interment in this phase. Genome-wide data was recovered from the individual buried in grave 11. Mound-shell 4 represents the final stage of the first construction phase. The second and third construction phases (mound-shell 5-7) likewise date to the North Caucasian epoch, yet three interments were catacomb graves. Mound-shells 2, 5 and 6 were surrounded by stone circles built with mid- and large-sized river pebbles. Three later graves were Sarmatian (Late Iron Age) and Medieval and several others could not be securely dated.
One individual from the earlier North Caucasian construction produced genome-wide data:

- BU2001.A0101.TF1 (BZNK-305/1), kurgan 1, grave 11 was a poorly preserved inhumation in a wooden chamber inside of a classical *North Caucasus* grave pit. The grave inventory included a bronze weapon, bronze pins and ornaments as well as a collection of stone beads. The head of a large animal was excavated from the stone package on top of the grave. Dating: 2867-2581 calBCE (4125±19 BP, HD-29619)

Goryachevodskiy 2, Russia
N 44.031°, E 43.129°

Excavation 'Institute of Archaeology of the Caucasus', Nalchik 1999, licence № 1999 (B.M. Batchaev)
The site of Goryachkevodsky 2 near the city of Pyatigorsk is situated in the North Caucasian foothills neighboring a hilly landscape formed by volcanic activity. The Konstantinov plateau where the site is situated is well known for dense clusters of burial mounds, which are chiefly found at the watersheds between smaller creeks. The vegetation zone is forest steppe environment[3]. The location of the site is at the right side of the Podkumok river, approximately 25 km downstream from Beliy Ugol 2. The site consisted of at least five mounds, severely affected by agricultural activities. Mound 3 was excavated in 1999 prior to road construction by the Institute of Archaeology of the Caucasus[28]. It was severely damaged but formerly had a diameter of at least 45 m and was still 1.7 m high. This mound was one of the smaller features of this cemetery. The first construction in the mound consisted of three late Maykop burials (graves 4, 5, 6), which were placed in the centre in parallel rectangular pits and covered by a long, rectangular stone package. Graves 4 and 5 were dug into the ground after grave 6, which was situated on the former ground level. Grave 5 is one of the few dendrochronologically dated complexes in the North Caucasus. It is 2 years younger than grave 12 from Marinskaya 5[29]. The late Maykop grave 3 was placed directly on top of the central grave 6. Graves 8 and possibly 7 also belong to the late Maykop phase. The Maykop mound was bordered by a stone circle. A series of interments (graves 2, 9, 10) attributed to the North Caucasus formation were inserted into this circle, partly destroying the Maykop construction. The last construction on this mound was a series of four or five graves of the post-Catacomb horizon. Several graves from this site were subjected to analyses of isotope composition and radiocarbon reservoir effects[27]. The paired samples from grave 5 (grave 9 in[27]) produced reproducible dates suggesting no reservoir offset[27].
Two individuals produced genome-wide data:

- I1723, kurgan 3, grave 9 [or 5], which is a standard *North Caucasus* grave in a wooden construction covered with stones, cutting the Maykop stone circle. No objects were found in the grave but remains of a cattle skull were on top of the grave cover. Dating: human bone 2877-2627 calBCE (4150±31BP, OxA-16190), animal bone 4158±30 BP, OxA-16191). There is no reservoir offset between the two dated bones.
- GW1001.A0101.TF1 (BZNK-287/1), kurgan 3, grave 10, a likewise standard *North Caucasus* grave pit covered by stones, cutting the Maykop stone circle. The grave contained no objects. Dating: 2881-2671 calBCE (4174±24BP, MAMS-29807)

Ipatovo 3, Russia
N 45.685°, E 42.921°
Excavation 'Nasledie', Stavropol 1998-1999, licence №1998-177 (A. B. Belinskiy)
The big mound of Ipatovo (Ipatovo 3, mound 2) was part of a series of linear alignments of mounds that run west-east crossing the system of the Kalaus river. Today the slightly hilly zone around the site is part of the North Caucasian herb and grass-steppe zone, while the Kalaus valley itself is part of the desert-steppe[3]. However, the vegetation likely shifted between desert and grass steppe in prehistory. During the construction of the mound, the climate was generally warmer and more humid than it is today and the landscape was more forested.
Mound 2 was the largest in a group of at least eleven mounds visible from aerial images. It was excavated due to construction work and yielded a total number of 195 burials in 11 construction phases[30]. 151 of the interments were part of a Nogay cemetery dating to the 18th century. One grave held the burial of a splendidly furnished Sarmatian woman[31]. The mound construction and 34 graves were assigned to a Bronze Age date and published in a monograph. Several of the Catacomb epoch interments were accompanied by wooden wagons[32]. The first three mound-

shells are associated with graves that belong to an Eneolithic or Steppe-Maykop tradition. Grave 187, which is part of this study and has produced genome-wide data, is the founding grave of the entire mound. It was attributed to a local Eneolithic tradition in the monograph[30]. After re-evaluation of the data, we would now rather group it with the Steppe Maykop complexes.
This individual produced genome-wide data:

- IV3002.A0101.TF1 (BZNK-293/1), kurgan 2, grave 187, 35-45-year-old male in an oval pit covered by stone slabs. No inventory was discovered. Dating: 3628-3127 calBCE (4630±50BP, GIN-10297)

Kabardinka, Russia
N 43.826°, E 42.716°
Excavation 'Nasledie', Stavropol and Eurasia-Department DAI, Berlin 2011, licence №2011-50 (A.B. Belinskiy)
The cemetery of Kabardinka is the only site in this study which is situated in the mountain part of the North Caucasus. The site is located in a mid-height mountain environment at the edge of a plateau overlooking the mineral spa of Kislovodsk. The excavation of one of the burial mounds and a stone circle in 2011 was part of a long-term archaeological investigation in the nearby settlement Kabardinka 2 and related sites[24]. The mound was constructed by communities of the North Caucasian tradition, but a later phase can be associated with a local group of the post-Catacomb/Late Bronze Age 1 horizon. The burial in the centre of the neighbouring large stone circle and the associated burial of a child in a small stone circle also date to the later phase of the cemetery. Both revealed no indications for a mound embankment. The graves predate the neighbouring Late Bronze Age 2 settlement and the shift from sedentary pastoralism to combined mountain agriculture associated with this epoch. Two Late Bronze Age 1 individuals of the site produced genome-wide data:

- KBD001.A0101+B0101(BZNK-123/2+4), kurgan 2, grave 1b. Deep burial covered and pit lined by stones and surrounded by a massive stone circle. The grave had no inventory. Dating: 2196-1977 calBCE (3690±30BP, UGAMS-13455)
- KBD002.A0101 (BZNK-124/4), kurgan 2, grave 2, child in an oval pit. The skeleton was equipped with a few ornaments from shell and a ceramic vessel. Dating: 2188-2026 calBCE (3695±20BP, MAMS-19727)

Kaps, Armenia
N 40.866667°, E 43.716667°
L. Petrosyan (Institute for Archaeology and Ethnology in Yerevan), L. Yeganyan & H. Khachatryan (Museum Sirak)
Expedition of the National Academy of Sciences of Armenia (rescue excavation, L. Petrosyan). The tombs of Kaps are located on the southern and eastern slopes of a ridge on the left bank of the Akhuryan River. Out of four stone-chamber tombs found only one (2 x 2 x 1m) with a double-burial had remained untouched. The entrance of the chamber was located in the eastern wall. There are three synchronous burials in this chamber. Two human skeletons lying on their right side were found directly beside the western wall, whereas the third burial was documented behind the immobile entrance stone. The head of the latter skeleton was oriented towards to the east. Early Bronze Age Red-Black-ceramic vessels and bronze objects were found in these chambers. A synchronous settlement was documented near the burial ground, including portable fire-installations and ceramics. However, the settlement had been damaged during construction works. The archaeological site of Kaps is dated to the second stage of the Early

Bronze Age (EBA; 4th-3rd millennium). The core area of the EBA Kura-Araxes culture is in Mesopotamia between the Kura and Araxes rivers. EBA cultural remains dating from the end of the 4th millennium to 3rd millennium BCE have been documented in the south-western regions of the highlands, in the north-western part of the Iranian plateau, and in the North Caucasus (intermediate zone). The peripheral zone of the Kura-Araxes culture extends to northern Syria and the Levant. Chronologically, Kura-Araxes extends to the last quarter of the 3rd millennium BCE. The Kura-Araxes culture represents a period in which complex societies were formed in the South Caucasus, which was later succeeded by a period of early statehood, which represents a new archaeological culture. Two individuals produced genome-wide data:

- ARM001.A0101.TF1: 3501-3128 calBCE (4595±30BP), 3631-3369 calBCE (4695±40BP, KIA44691)
- ARM002.A0101.TF1: 3338-3030 calBCE (4475±30BP, KIA44692), 3341-3030 calBCE (4480±30BP) 3366-3106 calBCE (4545±25BP, KIA44693)

Klady and Dlinnaya Polyana, Russia
N 44.384896°, E 40.390285°
Excavation Institute for the Material Culture History, Russian Academy of Sciences, Saint-Petersburg, (A.D. Rezepkin)
The Klady cemetery is located in the foothills area of the Northwest Caucasus near the village Novosvobodnaya and includes about 40 kurgans of different sizes. The largest of them, the so-called 'Silver Mound' (mound 11) was about 12 m high and 120 m in diameter. The cemetery was excavated in 1898-99 (N.I. Veselovsky), 1979-1991 (A.D. Rezepkin) and in 2013-2017 (V.A. Trifonov)[1]. In archaeology the Klady cemetery is regarded as the most vivid example of the relationship between the European, Caucasian and Ancient East cultures in the Early Bronze Age. Currently, the site is attributed to the Novosvobodnaya variant of the Maykop phenomenon and dated to the second half of the 4th millennium BCE. All graves with human remains that have been sampled for DNA-analysis are published.
Four individuals produced genome-wide data:

- I6266, KLADY2, KLD89-11/22, kurgan 11, grave 22. The adult human skeleton was found positioned on its left side in a crouched position and the head pointing SE, accompanied by two ceramic pots typical for the *Maykop-Novosvobodnaya* variant, and a flint flake. Dating: 3500-3000 BCE
- I6267, KLADY4, KLD89-11/43, kurgan 11, grave 43. The burial was placed in a simple rectangular pit with rounded corners (1,95 x 1,5 m). The adult human skeleton was found positioned on its left side in crouched position with hands raised in front of the face and head pointing south. The grave goods included three roe deer skulls (*Capreolus*?), two ceramic pots typical for the *Maykop-Novosvobodnaya* variant, and two bone pins. Dating: 3614-3362 calBCE (4675±70BP, OxA-5058)
- I6268, KLADY5, KLD89-11/50, kurgan 11, grave 50. The burial was placed in a simple rectangular pit with rounded corners (2,3 x 1,4m). The adult human skeleton was found positioned on its right side in a crouched position with hands raised in front of the face and the head pointing SSW. The grave goods consisted of three ceramic pots typical for the *Maykop-Novosvobodnaya* variant, and a broken hammer (?) made from antler. Dating: 3692-3532 calBCE (4835±60 BP, OxA-5059)
- I6272, KLADY_DP, KLD89-DLP, Klady, Dlinnaya Polyna. The grave was found under a small mound. The burial was placed in a shallow square pit (2,15 x 2 x 0,15m) with floor and walls lined with pebbles. An adult human skeleton was found positioned on its right side in a crouched position with hands raised in front of the abdominal cavity

and its head pointing south. The grave goods consist of two ceramic pots typical for the *Novosvobodnaya* variant and a polished and shafted stone hammer. Dating: 3500-3000 BCE.

Kudachurt, Russia
N 43.354032°, E 43.721893°
Excavation 'Institute of Archaeology of the Caucasus', Nalchik 2004-2006
The flat cemetery of Kudachurt K14 is one of the rare cemeteries with heterogeneous burial rites of the Bronze Age that has been excavated almost to full extent. The location of the site is in the foothills of the northern flank of the Greater Caucasus, not far below where the river Balkar Cherek flows out of a 600 m deep gorge. The environment is mid-altitude alpine forest vegetation[3]. The final Middle and Late Bronze Age cemetery (ca. 2200-1650 calBCE) on the right river terrace was part of a long-term rescue excavation due to the construction of a hydrological station during which 219 burials were excavated, of which 130 date to the Bronze Age. The other burials are associated with the Early Medieval Alanian population and belong to a large agglomeration of fortified settlements and necropolises dating to the 1st century AD. Interdisciplinary analyses of the Kudachurt cemetery are currently in preparation for publication as part of the doctoral thesis of Katharina Fuchs, Kiel University[33]. The thesis evaluates indicators of social inequality, oral health and diet with regard to socio-economic issues during the key period of the Middle to Late Bronze Age transition in the piedmonts. Two individuals of the final Middle Bronze Age horizon produced genome-wide data:

- KDC001.A0101.TF1 (BZNK-301/1), kurgan 14, grave 218.1/2, individual 218.1_3. The complex was a catacomb grave with two layers of inhumations. The skeletons were placed with their grave goods in crouched positions. A minimal number of eleven individuals were found, four in the upper (218.1) and seven in the lower level (218.2). Like the majority of burials at Kudachurt the grave contained ceramic vessels, animal remains, a few bronze weapons and jewellery. Dating of the animal sample from layer 1: 1953-1776 calBCE (3548±23BP, MAMS-110560); dating of the animal sample from layer 2: 1971-1777 calBCE (3554±23BP, MAMS-110561)
- KDC002.A0101.TF1 (BZNK-300/1), kurgan 14, grave 50, shallow and tight grave with two ceramic vessels and animal remains. Dating of the animal sample: 1879-1692 calBCE (3456±25 BP, KUD14gshdl_050).

Lysogorskyja 6, Russia
N 44.05336°, E 43.21034°
Excavation 'Nasledie', Stavropol 2015, licence №2015-70 (T. A. Gabuev)
The site of Lysogorskyja 6 is a loose agglomeration of burial mounds at the right bank of the river Podkumok. The location and environmental conditions are comparable to the site of Goryachevodsky, which is approximately 20 km away. The excavation of mound 3 took place due to construction works in 2015. Prior to excavation the huge mound was 50 x 65 m in diameter and 7.2 m high. It held a complex mound construction of mud blocks on top of the central burial 4, which dates to the North Caucasus cultural formation. Unlike most Caucasian burial mounds it revealed only one single construction layer associated with grave 4. The other three interments were dated to the Sarmatian period. The individual that produced genome-wide data was the founding grave of the mound:

- LYG001.A0101.TF1 (BZNK-297/1), kurgan 3, grave 4, *North Caucasus* burial in a huge stepped pit inside a wooden frame. The grave was covered by a massive stone

package and held an interment with a rich bronze inventory, including arms, a miniature vessel, jewellery, and remains of textiles. Dating: 2863-2581 calBCE (4122±23BP, MAMS-29825)

Marchenkova Gora 13, Russia
N 44.583183°, E 38.176667°
Excavation Institute for the Material Culture History, Russian Academy of Sciences, Saint-Petersburg, 2003 (V. A. Trifonov)
The group of 14 dolmens at Marchenkova Gora is located in the mountainous area of the NW Caucasus near Gelengik at the Black Sea coast. The site was explored in 2003 and 2007. Dolmen 13 represents a collective megalithic tomb typical in the Western Caucasus, with a characteristic entrance hole of 25 x 45 cm in diameter. This entrance enabled access to the burial chamber for periodic interments and was closed by a stone plug. The burial chamber of the dolmen contained human remains from about eight individuals of different ages and sexes. The human remains were accompanied by pendants made from wild boar tusks and flint arrowheads. One individual produced genome-wide data:

- I2051, MG13, MG-03 D-13, Marchenkova Gora, Dolmen 13. Dating: 1410-1210 calBCE (3045±80BP, Le-7053)

Marinskaya 3, Russia
N 43.925°, E 43.530°
Excavation Lomonosov Moscow State University, Institute of Archaeology RAS, 'Nasledie' 2007, licence № 2007-687 (A.R. Kantorovich), 840 (V. Ye. Maslov)
In 2007 a huge burial mound near the village of Marinskaya was excavated by the Lomonosov Moscow State University, the Institute of Archaeology RAS and the local heritage organisation 'Nasledie'[34]. The excavated mound 1 was part of a cemetery of then three visible and several ploughed-over burial mounds on the high terrace of the river Kura. The site is situated in an herb-grass steppe environment[3], which was formerly intersected by forests. The barrow was more than 4 m high and about 40 m in diameter and surrounded by a circular ditch about 2.5 m deep, which was visible on aerial and satellite images and later partly excavated. The excavation of the mound revealed two main phases of construction: the first mound-shell dates to the Maykop period. Beneath this shell is a deep rectangular burial pit with an undisturbed late Maykop burial (grave 18) placed on a floor paved with river pebbles[34]. The pit was dug into the ground and had a wooden ceiling, which was covered and surrounded by a rectangular stone construction. The walls of the pit were plastered and painted in red and bright yellow. Grave 18 is dendrochronologically dated. The first mound-shell was built on-top of this grave and the empty stone construction of grave 2 possibly also belongs to this phase. The second mound-shell dates to the Middle Bronze Age and was constructed in several layers, during which 14 graves were interred. Six graves are associated with the North Caucasus cultural formation (grave no. 3, 6, 12, 13, 16, and 17), while eight graves (no. 5, 7-9, 11, 14, 15, and 17) belong to the Catacomb cultural tradition (Suvorovo variant). The stratigraphy suggests that the shallower graves had initially been placed around the Maykop mound and were only later covered by the layers of Middle Bronze Age construction phases. Finally, an Early Iron Age grave and a Sarmatian grave 4/10 were interred in the Bronze Age mound, both of them severely damaged by later grave robbers.
One individual produced genome-wide data:

- MK3003.A0101.TF1 (BZNK-051/1), kurgan 1, grave 7, poorly preserved inhumation of the *Catacomb* epoch in a T-shaped catacomb with bronze ornaments and some animal bones. Dating: 2577-2476 calBCE (4020±22BP, MAMS-29809)

Marinskaya 5, Russia
N43.905354°, E 43.521883°
Excavation Lomonosov Moscow State University, Institute of Archaeology RAS, 'Nasledie' 2009, licence № 2009745 (A.R. Kantorovich), 2009-762 (V.Ye. Maslov)
The huge, single-standing burial mound Marinskaya 5, situated 2.3 km southwest of the mound group Marinskaya 3 was excavated in 2009 by a team of the Lomonosov Moscow State University, the Institute of Archaeology RAS and the local heritage organization 'Nasledie'. The kurgan is situated on the high terrace of the river Kura. The mound was slightly oval with a diameter of 34 to 40 m and 4.3 m high. At a distance of 10-16 m a ditch of 1.5 m depth surrounded the mound, which was detected on aerial images and excavated later. In the central part of the mound, the excavations uncovered three mound-shells or construction phases dating to the Early Bronze Age Maykop epoch and one that was constructed during the Middle Bronze Age. The first constructive feature in the mound was the above-ground burial vault of grave 33, surrounded by an oval fencing built from river pebbles. The first shell, an earth and stone construction, was built directly on top of this construction. Two following early Maykop burials 32 and 34 were entrenched in the centre. The second mound-shell was associated with late Maykop burials 12 and 16 and was added later. The third mound-shell was constructed after the interment of late Maykop grave 25. These mound-shells were complex earthen constructions and each was covered by a stone shell. All burials except grave 25 were situated on top of each other in the centre of the mound. Grave 12 is dendrochronologically dated[29], all others inhumations are radiocarbon dated. The Maykop stratigraphy has recently been published in Russian[35]. In several graves, among them late Maykop grave 25 and North Caucasus graves 19, 23, 30/30a, paired cattle skulls were found. They place this site among one of the earliest where the use of cattle as draught animals is documented in a chronological sequence[8]. After a hiatus of 600 years, 18 graves (no. 3, 10, 13, 15, 17-24, 26-29, 30A, and 31) dating to the North Caucasus cultural formation were added, including another mound shell. Two graves (no. 4 and 14) dated to the Late Bronze Age/post-Catacomb epoch based on burial practice and radiocarbon dates, and two nearly destroyed burials (no. 8 and 11) perhaps also date to the Bronze Age. One catacomb grave, also dating to the local Middle Bronze Age, intersected the ring of the North Caucasus graves and destroyed an earlier interment (no. 30/30A). During the Late Iron Age five additional graves were added in the centre of the mound. which can be attributed to the Sarmatian epoch. Grave 1 can be associated with the Early Iron Age of the late 8th to early 7th century BCE and the others (no. 2, 5, 6, 9) date to the early Sarmatian epoch, i.e. the 3rd to 1st century BCE.

- MK5005.C0101.TF1 (BZNK-079/1), kurgan 1, grave 32, an early *Maykop* secondary interment into the second mound-shell on top of the first burial. The grave was without inventory but is according to the stratigraphy associated with the early Maykop complexes. Dating: leather 3640-3370 calBCE (4720±25BP, UGAMS-13047); human bone 3341-3098 calBCE (4496±26BP, MAMS-11212)
- MK5001.A0101.TF1+ B0101.TF1 (BZNK-065/5+3), kurgan 1, grave 12, a partly disturbed *Late Maykop* inhumation slightly south of the centre with bronze weapons and a ceramic vessel. The grave is dendrochronologically dated with an end date of 3279 BCE + 25 years for missing sapwood[29]. Dating of the skeleton: 3348-3035 calBCE (4491±32BP, MAMS-110555)

- MK5008.B0101.TF1 (BZNK-066/3), kurgan 1, grave 16, a *Late Maykop* inhumation in the center of the mound with a wooden vessel(?). Dating: 3364-3107 calBCE (4544±25BP, MAMS-29810)
- MK5004.A0101.TF1+D0101 (BZNK-073/4+2), kurgan 1, grave 25, which was a disturbed *Late Maykop* inhumation burial in a square burial chamber. Bronze weapons, a golden earring and a ceramic vessel remained from the inventory. Additionally, two cattle skulls with bronze nose rings and the remains of a possible yoke were found *in situ* outside the chamber. Dating of wood remains from the grave construction: 3347-3095 calBCE (4498±30BP, MAMS-110554)
- MK5009.A0101.TF1 (BZNK-064/2), kurgan 1, grave 10, a *North Caucasus* grave inside a well preserved wooden massive construction in a deep grave shaft. The inventory included a stone object and animal bones. This complex is likewise dendrochronologically dated with an end date of 2644 BCE + 13 years uncertainty for missing sapwood[29]. Dating of the skeleton: 2884-2636 calBCE (4175±31BP, MAMS-110548)

Nevinnomiskiy 3, Russia
N 44.73486°, E 41.938749°
Excavation 'Nauchno-issledovatelskiy institute arkheologii I drevney istorii Severnogo Kavkaza', Stavropol 2012, licence №2012-1145 (S. V. Mjachin)
The site is situated in the steppe zone of the Central Caucasus at the junction of the rivers Bolshoy Zelenchuk and Kuban, on top of a river terrace of the Kuban and was partly excavated in 2012. The cemetery itself comprised 25 mounds. Mound 6 east was initially built on top of a Yamnaya burial. During Bronze Age and Late Iron Age Sarmatian times 13 additional burials were dug into the initial mound. The largest group comprises interments of the post-Catacomb horizon. One individual of *post-Catacomb* date produced genome-wide data:

- NV3001.A0101.TF1+B0101.TF1 (BZNK-312/1+3), kurgan 6, grave 5, an inhumation in an oval burial pit associated with metal casting equipment, of which only very few are known for this period, and animal bones. Dating: 2116-1925 calBCE (3631±22BP, MAMS-29812)

Nogir 3, Russia
N 43.084417°, E 44.632667°
Rescue excavation 2016, licence №2016 (V.S. Sanakoev)
The site of Nogir 3 is situated in the modern town of Vladikavkaz. Mound 3 was heavily damaged during earlier road construction and was excavated as part of rescue excavations in 2016, when only a few artefacts could be saved. One individual produced genome-wide data:

- OSS001.A0101.TF1 (BZNK-313/1), kurgan 3, grave 4, individual 1 was art of a *Maykop* burial, yet only a few remains of bones and ceramic vessels have been preserved. Dating: 3695-3545 calBCE (4857±23BP, MAMS-29813)

Progress 2, Russia
N 43.822691°, E 43.350278°
Excavation 'Nasledie', Stavropol 2009-10, licence №209-545 (S. Ja. Berezin)
Progress 2 is located in the piedmont steppe zone of the North Caucasus, in the same environmental zone as the sites of Marynskaya 3, which are 20 km away. Five of the ten mounds

of the group were excavated prior to gravel extraction from the former riverbed of the river Malka. The river terraces here are dotted with burial mounds, of which several were excavated in the 1970s. The mounds were located on the first terrace on the left side of the river. Several mounds were clustered together, while mounds 5 and 10 were slightly separated. Mounds 1 and 4, from which the sampled individuals come, were part of one cluster. Most of the burials in all mounds date to the North Caucasus cultural formation, but mound 1 and 4 were originally built on top of Eneolithic inhumations. Two of them were sampled and produced genome-wide data. Mound 1 was the largest mound with a diameter of 40 m and a height of 2.2 m. Its construction comprised three phases with two stone shells and a stone circle. In the 18-19th century the location was used as a cemetery by the local Islamic population. The founding grave 37, which was sampled for aDNA analysis, dates to the Eneolithic epoch and is related to the aforementioned group of Don-Caspian-Caucasus Eneolithic complexes. Of the remaining burials in this mound, two are associated with the Yamnaya epoch, and eleven graves date to the Middle Bronze Age with inventories attributed to the North Caucasus formation.
Mound 4 was smaller with a diameter of 20 m and a remaining height of only 0.3 m. The founding graves were two Eneolithic burials, graves 9 and 12, which were found side by side and revealed practically identical radiocarbon dating. Both skeletons were thickly packed in red ochre and grave 12 revealed a complex trepanation[36]. One grave in this mound is associated with Yamnaya, four with the Middle Bronze Age (North Caucasus) and one burial dates to the Sarmatian period. Three individuals from Progress 2 produced genome-wide data:

- PG2001.B0101.TF + B0201.TF (BZNK-113/4), kurgan 1, grave 37, was the *Eneolithic* founding grave in mound 1 was found in an oval pit, thickly packed in red ochre. The grave goods consisted of a long flint blade, a flint adze, a flint projectile head and another flint object. A radiocarbon doublet of charcoal and human bone revealed a strong reservoir-effect in the human bone date. Dating: human bone 4991-4834 calBCE (6012±28BP, MAMS-110564), charcoal 4336-4173 calBCE (5397±28BP, MAMS-110563)
- PG2002.A0101.TF (BZNK-303/1), kurgan 1, grave 25b. The burial was placed in a rectangular pit attributed to the *North Caucasus* culture and was equipped with bronze ornaments and a collier of bronze and gagat beads. On top of this grave a second North Caucasus grave 25a without grave goods was discovered. Dating: 2483-2342 calBCE (3929±22BP, MAMS-29815)
- PG2004.A0101.TF + C0101.TF (BZNK-062/1+3), kurgan 4, grave 9, was one of the two *Eneolithic* founding graves in the mound. The individual was found in a shallow grave pit packed in a thick layer of red ochre. The grave inventory contained the fragment of a ceramic vessel, a long flint blade and a bone object. Dating: 4233-4047 calBCE (5304±25BP, MAMS-11210)

Rasshevatskiy 1, Russia
N 45.537249° E 41.116544°
Excavation 'Nasledie', Stavropol 1998-2000, licence №2000-252 (V. L. Rostunov)
The burial mound cemetery Rasshevatskiy 1 is one of several groups of mounds that run in long lines from West to East. The vegetation today is an herb-steppe with larger patches of forest steppe vegetation[3]. The actual cemetery included 27 mounds and extended across 2 km, with distances between mounds varying from 30 to 130 m. Mound 21 with an oval to ogival shape was 110 m long, 85 m wide and 6 to 6,4 m high[37, 38, 39]. It was excavated during rescue excavation in 2000 and is one of the two largest mounds in the group. The mound was built in five construction layers or mound-shells of different forms and earth compositions. Mound-shell 1 was constructed from dark-brown and brown loamy substrate in an egg-shaped form

with an axis running North-South. It is associated with the Steppe Maykop formation, the founding burial is grave 14. Mound-shell 2, still egg-shaped, was built over the empty burial pit 3 in the Yamnaya period; graves 9, 11 and 13 were inserted into this shell and are also associated with the Yamnaya epoch. Two of the individuals from this epoch were sampled and produced genome-wide data, grave 11 revealed the oldest evidence of an early form of *Yersinia pestis*[40]. The second mound was entirely covered by a thin layer of light grey river-clay. The third mound-shell is associated with Novotitorovskaya groups. Its founding burial was grave 7, which was accompanied by a dismantled wagon. Graves 15, 17, 18 and probably 19 are associated with mound-shell 3. By this time, the mound was transformed into an oval construction with a flat top. Mound-shell 4, a construction of several layers among them dark- and light brown loamy substrates, is associated with North Caucasus grave 16. Grave 21 of the same cultural formation possibly also belongs to shell 4. This was the third individual that produced genome-wide data. After that, the mound was considerably enlarged and had a flat top-platform, but its axis was still running North-South. The construction of mound-shell 5 included a considerable enlargement and a turn of the main axis from North-South to East-West. The founding burial, grave 8, is dated to the early Catacomb period with three wagons standing beside the grave pit (labelled initially 'grave 6'). Graves 2, 4, 12, the sacrificial pit 'grave' 1, and possibly grave 20 are also associated with the late Catacomb phase. Grave 5 on top of the mound dates to the medieval period, and the empty grave(?) pit 22 cannot be securely dated. The complexes of mound 21 from Rasshevatsky are part of a larger bioarchaeological study and are scheduled for full publication in 2019/20. Three individuals produced genome-wide data:

- RK1001.C0101 (BZNK-034/4), kurgan 21, grave 11, a typical *Yamnaya* burial with an individual in crouched position on the back, legs bent upwards. No inventory was associated with this grave. Dating: 2879-2673 calBCE (4171±22BP, MAMS-29816), when modeled according to stratigraphy the date changes to 2880-2798 calBCE.
- RK1007.A0101 (BZNK-035/2), kurgan 21, grave 13, which is a typical *Yamnaya* burial in a crouched position on the back. The deceased was accompanied by a ceramic vessel. Dating: 3308-3026 calBCE (4447±22BP, MAMS-29818; when modeled according to stratigraphy the date changes to 3262-3025 calBCE).
- RK1003.C0101 (BZNK-042/3), kurgan 21, grave 21, a *North Caucasus* interment in a deep, rectangular burial pit, covered by wooden planks. Grave goods comprised a bone pin and a silver earring. Dating: 2802-2761 calBCE (4218±24BP, MAMS-29817), when modeled according to stratigraphy.

Rasshevatskiy 4, Russia
N 45.535978° E 41.059517°
Excavation 'Nasledie', Stavropol 1999, licence №1999-62 (J. B. Berezin)
Only 4 km to the southwest of Rasshevatsky 1 another parallel line of mounds runs west-east - Rasshevatsky 4. Here six mounds were excavated during rescue excavations in 1999. The most eastern was mound 1 with an oval form, a diameter of 18-35 m, and a preserved height of 0.36 m. In the mound three Catacomb burials and two burials in pits were discovered, dating most likely to the post-Catacomb horizon. Two individuals produced genome-wide data:

- RK4001.A0101 (BZNK-284/1), kurgan 1, grave 4, a poorly preserved inhumation in crouched position without inventory. Dating: 2338-2210 calBCE (3837±22BP (MAMS-29819)

- RK4002.B0101 (BZNK-304/2), kurgan 1, grave 5, a *Catacomb* grave in a T-shaped catacomb with bent up knees. No inventory. Dating: 2618-2497 calBCE (4050±22BP (MAMS-29820)

Sharakhalsun 6, Russia
N 45.725651°, E 43.[23]986933°
Excavation 'Nasledie', Stavropol 2001, licence №2001-791 (A. V. Yakovloev)
The sites of Sharakhalsun are located about 70 km south of Elista in one of the densest clusters of burial mound cemeteries in the Caspian steppe. The cemetery is one of several hundred similar formations in this area and like most of them organized in a line running West-East. It is situated on the right bank the river Kalaus not far from its entry into the major river Manych, which is now the Manych water reserve. More than 330 graves were excavated by different 'Nasledie' field campaigns in this area and through rescue excavations during the construction of the Manych water reserve, which resulted in a large number of excavated complexes. In addition, detailed seminal studies on the dating but also subsistence and mobility patterns were conducted by Natalya I. Shishlina not far from the Sharakhalsun sites[5]. The studied mound 2 in the cemetery of Sharakhalsun 6 was uncovered during rescue excavations in 2001. The mound was 50 m in diameter and 3 m high. The stratigraphy is not entirely clear, but the first mound was constructed by communities of the early Steppe Maykop formation in the late 4th millennium BC, of which founding grave 17 produced genome-wide data. Grave 16 was added shortly after. The second mound-shell was also built by early Steppe Maykop groups and graves 12 and 15 date to this period[41]. The third Maykop cluster dates to the second half of the 4th millennium BC. It includes grave 6, 11 and the atypical grave 18, which are among those that produced genome-wide data. This grave belongs to a specific group, with influences from Maykop and Yamnaya traditions[5]. During the 3rd millennium, Yamnaya groups used the Maykop mound and added several graves in central positions and on the periphery as well as at least one new mound-shell. The last interments (graves 1, 2, 7, 8, and wagon grave 9) belong to the late Bronze Age Catacomb period. Empty grave 10 can only roughly be dated to the Middle Bronze Age. Mound 2 in Sharakhalsun revealed four complexes with remains of wooden wagons belonging to different cultural formations. It is one of few places with a concentration of wagon burials among the hundreds of excavated mounds in the vicinity and yielded the oldest dated wooden wagon so far in grave 18[8]. This individual probably was one of the first that adopted this new technology in the North Caucasian and Caspian steppe[42]. The complexes of Sharakhalsun are part of a larger bioarchaeological study and are scheduled for full publication in 2020/21. Five individuals produced genome-wide data:

- SA6003.B0101 (BZNK-010/2), kurgan 2, grave 9, a late *Catacomb* grave of an individual squeezed into a small and narrow catacomb and covered with the half of a four-wheeled wagon. Grave good include a wooden vessel, a ceramic incense burner and a bronze pricker. Dating: 2474-2211 calBCE (3890±40BP, GIN-12400)
- SA6013.A0101 (BZNK-009/2), kurgan 2, grave 11, affiliated with the *Steppe Maykop* had a ceramic vessel as grave good. Dating: 3355-3105 calBCE (4524±22BP, MAMS-29246)
- SA6010.A0101 (BZNK-008/3), kurgan 2, grave 13, typical *Yamnaya* burial position with a flint artefact. Dating: 2884-2679 calBCE (4185±23BP, MAMS29821)
- SA6001.A0101 (BZNK-004/3), kurgan 2, grave 17, *Maykop* founding grave in the mound, child burial in an oval catacomb with entrance pit, including a ceramic vessel. Dating: 3619-3369 calBCE (4673±28BP, MAMS-31143)
- SA6004.A0101 (BZNK-003/1+4), kurgan 2, grave 18, *Steppe Maykop*. This exceptional grave is an inhumation of a male individual sitting on a four-wheeled wooden wagon in

a narrow catacomb. No additional grave offerings were found. The physical anthropological examination of this individual revealed a large number of pre- and perimortem injuries[42]. Dating: wood, 3336-3105 calBCE (4500±40BP, GIN-12401)

Sinyukha, Russia
N 44.692, E 40.042
Excavation Oriental Museum, Moscow 2013, licence №2012-853 (V. R. Erlikh)
The site of Sinykha is one of the most western ones in this study. It is located in the valley of the river Belaya not far from the modern city of Maykop. This region is part of the piedmonts and situated in the forest zone today. However, the Northwest Caucasus was much less densely forested during the Bronze Age. Excavations in mound 1 were conducted in 2013 and revealed a total of 12 interments, most of which were extremely poorly preserved[43]. The founding grave 12[44], a Late Maykop context, contained three female individuals which produced genome-wide data. The individuals are placed together side by side, individual 1 in the east, and individuals 2 and 3 closely together. Three ceramic vessels, a bronze pin and stone beads were found as grave goods. This grave was surrounded by a stone circle of river pebbles found and covered by a stone shell. Two more graves also contained late Maykop objects, another two date to the Middle Bronze Age, and the last interment can be attributed to the Early Iron Age. Three related individuals produced genome-wide data (Supplementary Information 3):

- SIJ001.A0101.TF1 (BZNK-277/1), kurgan 1, grave 12, individual 1, 3329-3022 calBCE (4454±23BP, MAMS-31139)
- SIJ002.A0101.TF1 (BZNK-278/1), kurgan 1, grave 12, individual 2, 3349-3033 calBCE (4490±35BP, GrA 57656) and 3346-3101 calBCE (4505±23BP, MAMS31141)
- SIJ003.A0101.TF1 (BZNK-279/1), kurgan 1, grave 12, individual 3, 3349-3033 calBCE (4490±35BP, GrA 57656) and 3347-3101 calBCE (4507±23BP, MAMS31142)

Unakozovskaya, Russia
N 44.255876°, E 40.201139°
Excavation Adygey Pedagoical University, 1985-90, (N. G. Lovpache)
The Unakozaovskaya caves belong to a series of Darkveti-Meshoko type of Eneolithic sites which have been found in caves or below rock shelters in the valleys of the Northwest Caucasian rivers[45, 46]. The environment today is a densely forested, but until the end of the Middle Bronze Age (ca. 3500 calBP) the Northwest Caucasus was characterized by a very different vegetation cover with open landscapes and much less forest. The anthropological analysis of the skeletons by I. D. Potekhina does not mention an exact location[47]. The considerable Eneolithic layers in the caves yielded material, chiefly ceramic, that can be associated with the Drakveti-Meshoko formation, respectively the tradition of ‘pearl-ornamented ceramic’. The burial included one adult individual and three children, which were placed in crouched position, the heads covered by red ochre. Grave goods were stone bracelets, flint blades and bone objects. According to Potekhina the adult individual 1 was a male, about 60 years old. Individual 2a and 2b were children, placed in a crouched position on their backs directly on top of each other. Individual 2a was about 5-6 years old, while individual 2b was a sibling of 12-14 months. Individual 3 was probably 1-2 years old. All three *Darkveti-Meshoko Eneolithic* children produced genome-wide data:

- I2055, skeleton 2a: 4680-4486 calBCE (5718±29BP, OxA-43740)
- I2056, skeleton 2b: 4599-4456 calBCE (5687±30BP, OxA-43741)
- I1722, skeleton 3: 4536-4371 calBCE (5635±27BP, OxA-43742)

Velikent, Russia
N 42.179802°, E 48.066089°
Excavation Russian-American excavation 'Velikent', 1994 and 1997, licence №1994-443, № 1997-3 (R. G. Magomedov)
The skeletal material stems from the partly destroyed collective catacomb grave 1 excavated in 1994 and 1995 at Kharman-Tepe (Velikent cemetery mound II) and dated to 4080±75BP, 2805-2505 calBCE/2879-2474 calBCE)[10, 23]. Besides bronze artefacts a minimum of seven skulls were documented. The samples analysed were labelled 'individual 7', albeit belonging to at least to two different individuals.
Two individuals produced genome-wide data:

- VEK006.A0101.TF (BZNK-486/5): relative date 3000-2800 BCE
- VEK007.A0101.TF + VEK009.A0101.TF (BZNK-486/6;8): relative date 3000-2800 BCE; 2nd degree relative of VEK006

Vonyuchka 1, Russia
N 44.019962°, E 43.155538°
Excavation 'Nasledie', Stavropol 2010, licence №2010-130 (A. A. Kamykov)
The site of Vonyuchka 1 also belongs to the groups of burial mounds located on the Konstantinov plateau near the town or Pyatigorsk. The mounds are situated near a small creek, at the right side of river Podkumok. The site is close to Goryachevodsky 2 (1.5 km) and Lysogorskaya 6 (22 km), respectively. Thus, like the others, Vonyuchka 1 is situated in the North Caucasian piedmont steppe in a forest steppe-herb steppe environment[3]. Mound 1, which was excavated in a rescue excavation, had a diameter of 40 m and was up to 2 m high and contained beside the studied founding grave 8 other Bronze Age burials. One individual produced genome-wide data:

- VJ1001.B0101+D0101 (BZNK-3112+114/2), kurgan 1, grave 8, the founding grave in the mound discovered in a catacomb like construction. The bones were very badly preserved, but samples were taken directly after excavation. The entire skeleton was packed in a thick layer of dark-red ochre. Grave goods consisted of a ceramic vessel, a long flint blade, a flint scraper and a stone object. The skull of the individual revealed a partly healed trepanation[36]. Dating: 4332-4238 calBCE (5409±24BP, MAMS-29823), 4229–4065 calBCE (5314±21 BP, MAMS- 21327)

Zolotarevka 2, Russia
N 45.658644, E 42.605994
Excavation 'Nasledie', Stavropol 2000, licence №2000-400 (A. A. Kamykov)
Zolotarevka 2 is one of several burial mound cemeteries situated at the watershed of the rivers Kalaus and Bolshaya Kugulta, in a grass steppe environment[3]. The second group consisted of four mounds that were completely levelled by ploughing prior to excavation. All mounds were excavated in 2000 during rescue excavations. The fourth mound held three Yamnaya and one catacomb graves, which contained burial goods in the form of bronze ornaments, stone beads and ochre lumps. One individual produced genome-wide data:

- ZO2002.C0101 (BZNK-281/2), kurgan 4, grave 4/Ind. 2, The central grave 4 of this mound contained a large pit with four individuals placed in two couples in a typical *Yamnaya* pose. According to physical anthropology by M. M. Gerasimova and D. V.

Pezhemskiy three males and one female were buried together. Dating: not enough collagen (MAMS-29824); relative date 3000-2800 BCE

Supplementary Note 3

Ancient DNA authenticity and Kinship analysis

We performed X-chromosomal contamination tests for the male samples following an approach introduced by Rasmussen et al.[48] and implemented in the ANGSD software suite[49]. We applied the "MoM" (Methods of Moments) and "ML" (Maximum Likelihood) estimate from the "Method 1" likelihood computations (Supplementary Table 20). We note that the estimates of the highlighted individuals in are considered non-informative because of fewer than 200 X-chromosomal SNPs covered by at least two sequence reads. The estimated contamination rates for the other individuals are very low.

Supplementary Table 20. X-chromosomal contamination estimates for male samples based on ANGSD. "MoM" and "ML" are the estimated contamination rates using "Method 1" statistical approaches. "SE(MoM)" and "SE(ML)" are the standard errors. The estimates of the highlighted samples are not informative because of fewer than 200 SNPs covered by at least two sequences in these two samples. We only show the results generated using the new_llh version of ANGSD.

Sample	nSNP	MoM	SE(MoM)	ML	SE(ML)
KBD001	7627	0.00317	7.073E-04	0.00295	3.586E-14
PG2001	6274	0.00330	8.683E-04	0.00255	2.408E-14
PG2004	6146	0.00465	9.565E-04	0.00480	3.665E-14
MK5004	5117	0.00641	1.271E-03	0.00563	4.374E-14
NV3001	4922	0.00677	1.282E-03	0.00700	2.933E-14
SA6004	4395	0.00371	1.107E-03	0.00346	1.435E-14
KDC001.A0101	3758	0.00420	1.217E-03	0.00414	2.010E-14
SIJ002.A0101	3597	0.00434	1.330E-03	0.00387	1.254E-14
MK5001	3564	0.00872	1.854E-03	0.00729	2.242E-14
I2056	3434	0.00821	1.810E-03	0.00892	1.901E-14
ARM002.A0101	2887	0.00417	1.546E-03	0.00342	4.264E-15
ARM003.A0101	2840	0.00256	1.239E-03	0.00296	1.849E-15
RK1001.C0101	2278	0.00774	1.909E-03	0.00851	2.136E-14
RK4002.B0101	1954	0.00961	2.466E-03	0.00917	9.353E-15
GW1001.A0101	1613	0.00708	2.469E-03	0.00676	9.228E-15
RK4001.A0101	1594	0.00263	1.697E-03	0.00255	4.466E-15
LYG001.A0101	1460	0.00355	1.953E-03	0.00518	5.433E-15
I6266	1159	0.00409	2.830E-03	0.00398	5.313E-16
I6272	725	0.00967	4.592E-03	0.01067	4.761E-15
MK5009.A0101	578	0.00470	3.980E-03	0.00535	7.709E-16
SA6003.B0101	508	0.00750	5.342E-03	0.00670	1.797E-15
I6268	490	0.00801	5.587E-03	0.00776	3.069E-16
SA6013.B0101	437	0.00099	3.264E-03	-0.00083	4.075E-17
SA6010.A0101	435	0.00456	4.576E-03	0.00480	4.156E-16
VEK007.A0101	377	0.01142	6.562E-03	0.01070	1.514E-15
BU2001.A0101	280	0.01058	7.194E-03	0.01039	6.085E-16
VEK009.A0101	187	0.01818	1.205E-02	0.01690	6.151E-16
IV3002.A0101	139	0.01532	1.195E-02	0.01692	3.261E-16
I2055	125	0.02244	1.697E-02	0.01893	0.000E+00
I2051	46	-0.00353	3.634E-03	-0.00145	2.909E-18
I1723	25	-0.02082	1.258E-02	-0.00793	1.700E-17

I1720	17	0.21307	2.119E-01	0.11967	1.665E-16
OSS002.B0101	14	0.21758	1.519E-01	0.21259	0.000E+00
I2057	6	-	-	-	-
VEK008.A0101	1	-	-	-	-
MK5012.A0101	0	-	-	-	-
MK5007.B0101	0	-	-	-	-
MK5008.B0101	0	-	-	-	-
I6270	0	-	-	-	-

We used two methods *lcMLkin*[50] and *READ*[51] to determine genetic kinship. We identified two pairs (ARM003 and ARM002, VEK007 and VEK009) as the same individual or identical twins. We also found several pairs of first or second-degree relatives and removed them from most of the population genetic analyses (except for PCA and ADMIXTURE) to avoid bias resulting from a defined population consisting of too many closely related individuals.

An interesting finding is that three individuals SIJ001, SIJ002 and SIJ003 are suggested to be first-degree relatives while the pairs SIJ002-SIJ003 and SIJ001-SIJ003 are estimated to have a parent/offspring relationship (Supplementary Table 21 and 22).

Supplementary Table 21. ***lcMLkin*** **estimates for the kinship among samples.** We note that the results inferred from the samples of less than 10,000 SNPs might not be reliable.

Individual1	Individual2	k0_hat	k1_hat	k2_hat	pi_HAT	nbSNP	kinship
ARM003.A0101	ARM002.A0101	0.004	0.056	0.94	0.968	465648	Same individual/identical twins
VEK007.A0101	VEK009.A0101	0.016	0.125	0.859	0.921	124492	Same individual/identical twins
SIJ002.A0101	SIJ003.A0101	0.042	0.955	0.003	0.481	512539	Parent/offspring
SIJ001	SIJ003.A0101	0.043	0.953	0.004	0.480	528380	Parent/offspring
VEK007.A0101	VEK006.A0101	0.306	0.570	0.124	0.409	51676	1st degree
I2056	I2055	0.309	0.497	0.194	0.443	154264	1st degree
I2055	I1722	0.336	0.365	0.299	0.482	56352	1st degree
SIJ001	SIJ002.A0101	0.369	0.491	0.14	0.385	507701	1st degree
I2056	I1722	0.391	0.425	0.184	0.397	157598	1st degree
VEK008.A0101	VEK007.A0101	0.402	0.519	0.079	0.338	4017	1st degree
VEK009.A0101	VEK006.A0101	0.489	0.16	0.351	0.431	41734	1st degree
KBD001	KBD002	0.505	0.487	0.009	0.252	610846	2nd degree
VEK008.A0101	VEK006.A0101	0.532	0.455	0.013	0.240	1397	2nd degree?
MK5004	MK5001	0.581	0.402	0.017	0.218	543740	2nd degree
VEK008.A0101	VEK009.A0101	0.609	0.212	0.179	0.285	3163	2nd degree
I6270	I6266	0.779	0.085	0.136	0.178	3249	
SA6004	SA6001.A0101	0.870	0.121	0.010	0.070	542096	
I6270	I6268	0.873	0.120	0.007	0.067	2350	
SA6004	AY2003.A0101	0.875	0.121	0.004	0.064	541536	
AY2003.A0101	SA6001.A0101	0.900	0.079	0.021	0.061	536088	

Supplementary Table 22. *READ* estimates for the kinship among individuals.

If the Normalized2AlleleDifference (P0) < 0.625, the two individuals are identical twins or the same individual; If 0.8125 >= P0 >= 0.625, the two individuals are first degree relatives; If 0.90625 >= P0 >= 0.8125, the two individuals are second degree relatives; If P0 >= 0.90625, the two individuals are unrelated.

Individual1	Individual2	Normalized 2AlleleDifference	StandardError	NonNormalizedP0	NonNormalized StandardError	Kinship
ARM002.A0101	ARM003.A0101	0.476484744	0.002473	0.120666	0.000626	IdenticalTwins/SameIndividual
VEK007.A0101	VEK009.A0101	0.489773056	0.003321	0.124031	0.000841	IdenticalTwins/SameIndividual
I1722	I2055	0.722377924	0.005662	0.182937	0.001434	First Degree
SIJ002.A0101	SIJ003.A0101	0.72424976	0.002898	0.183411	0.000734	First Degree
VEK006.A0101	VEK009.A0101	0.724614444	0.005957	0.183503	0.001508	First Degree
SIJ001	SIJ003.A0101	0.729263306	0.002737	0.18468	0.000693	First Degree
I2055	I2056	0.73446415	0.004357	0.185998	0.001103	First Degree
VEK006.A0101	VEK007.A0101	0.746060227	0.005413	0.188934	0.001371	First Degree
I1722	I2056	0.765060959	0.004038	0.193746	0.001022	First Degree
VEK008.A0101	VEK006.A0101	0.772559084	0.02692	0.195645	0.006817	First Degree
SIJ001	SIJ002.A0101	0.776473599	0.003392	0.196636	0.000859	First Degree
VEK007.A0101	VEK008.A0101	0.809032346	0.017274	0.204881	0.004375	First Degree
VEK008.A0101	VEK009.A0101	0.850146878	0.01984	0.215293	0.005024	Second Degree
KBD001	KBD002.A0101	0.858628423	0.00317	0.217441	0.000803	Second Degree
MK5001	MK5004	0.869296338	0.003216	0.220143	0.000814	Second Degree

Supplementary Note 4
Sex determination and Y-chromosome analysis

Sex determination

We determined the sex of the newly reported samples in this study by counting the number of reads overlapping with the targets of 1240k capture reagent as discussed in Fu et al.[52]. We extracted the reads of high base and mapping quality (samtools depth -q30 -Q37) using samtools v1.3.1[53]. We calculated the ratios of the numbers of reads mapped on X chromosome or Y chromosome compared with that mapped on autosomes (X-rate = xCov/autCov and Y-rate = yCov/autCov). We observed two clusters in the X-rate to Y-rate scatterplot (Supplementary Figure 10), which we interpreted as a clear separation between males and females. We have identified 45 individuals (including 7 independent replicates in the library preparation) with an X-rate < 0.42 and a Y-rate > 0.26 as males and 20 individuals (including 1 independent replicate in the library preparation) with an X-rate > 0.68 and a Y-rate < 0.02 as females. We have one individual I2057 having an intermediate sex ratio, for which we could not reliably determine the genetic sex (Supplementary Table 23).

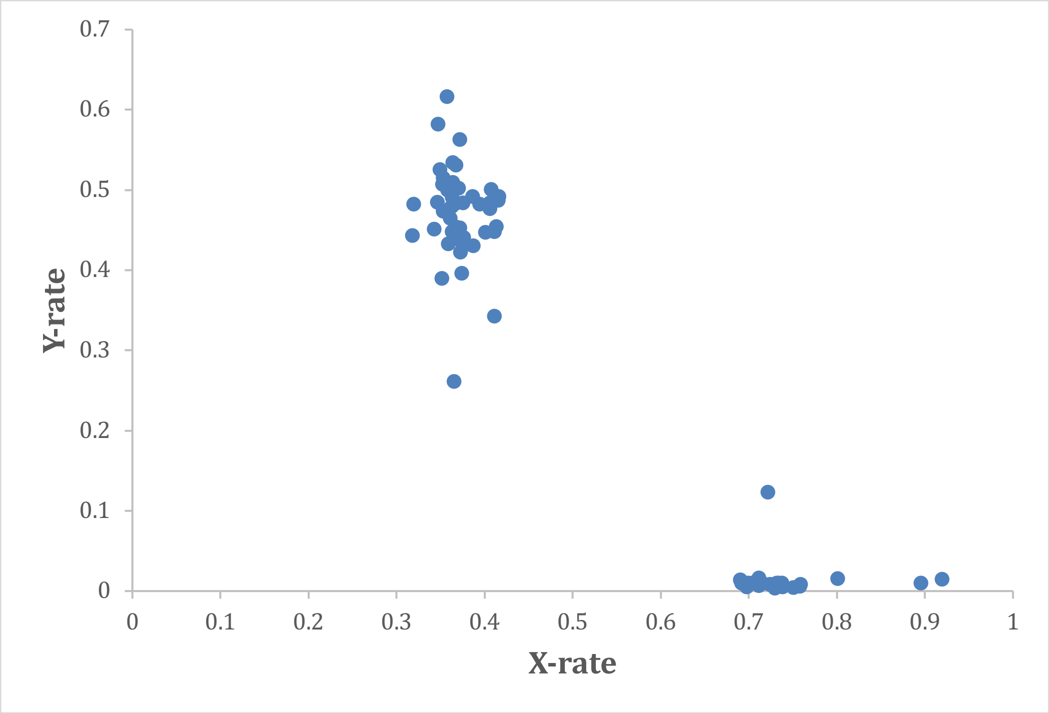

Supplementary Figure 10: X-rate to Y-rate scatterplot indicating genetic sex.

Supplementary Table 23: Numbers of reads mapped on X chromosome or Y chromosome versus the autosomes and Y-haplogroup typings.
Nseqs: the total number of reads mapped on the human reference genome.

Sample	Nseqs	xCov/ autCov	yCov/ autCov	Sex	Y haplogroup	
ARM001.A0101	2809801	0.7581	0.0090	Female	-	
ARM002.A0101	4192423	0.3632	0.5074	Male	G2b	
ARM003.A0101	4004409	0.3638	0.5096	Male	G2b	
AY2001.A0101	9141500	0.7028	0.0103	Female	-	
AY2003.A0101	6390930	0.6990	0.0101	Female	-	
BU2001.A0101	839079	0.3700	0.5023	Male	R1b1a2a2	
GW1001.A0101	3048224	0.3192	0.4830	Male	R1b1a2a2	
IV3002.A0101	787369	0.3717	0.4531	Male	?	
KBD001.A0101	6270235	0.3636	0.5348	Male	R1b1a2	
KBD001.B0101	7255204	0.3580	0.5005	Male	R1b1a2	independent replicate of KBD001.A0101
KBD002.A0101	6414457	0.7109	0.0163	Female	-	
KDC001.A0101	5303973	0.3572	0.6167	Male	J2b	
KDC002.A0101	3696144	0.6972	0.0058	Female	-	
LYG001.A0101	2696989	0.3427	0.4518	Male	R1b1a2	
MK3003.A0101	2393147	0.7391	0.0061	Female	-	
MK5001.A0101	2810302	0.3517	0.5076	Male	L	
MK5001.B0101	1678751	0.3628	0.4801	Male	L	independent replicate of MK5001.A0101
MK5004.A0101	5088083	0.3487	0.5254	Male	L	
MK5004.D0101	2229129	0.3657	0.4493	Male	L	independent replicate of MK5004.A0101
MK5005.C0101	360284	0.7318	0.0100	Female	-	
MK5007.B0101	3810	0.3649	0.2617	Male	?	
MK5008.B0101	160876	0.3858	0.4920	Male	?	
MK5009.A0101	1595419	0.3760	0.4413	Male	R1b1a2	
MK5012.A0101	42251	0.3734	0.3966	Male	?	
NV3001.A0101	4792208	0.3529	0.5151	Male	Q1a2	
NV3001.B0101	1901486	0.3671	0.5310	Male	Q1a2	independent replicate of NV3001.A0101
OSS001.A0101	1678590	0.7339	0.0102	Female	-	
OSS002.B0101	211342	0.4107	0.4484	Male	J	
PG2001.B0101	4746096	0.3751	0.4840	Male	R1b1	
PG2001.B0201	3940122	0.3601	0.4985	Male	R1b1	independent replicate of PG2001.B0101
PG2002.A0101	970812	0.7579	0.0062	Female	-	
PG2004.A0101	4990081	0.3631	0.4915	Male	R1b1	
PG2004.C0101	2876965	0.3619	0.4793	Male	R1b1	independent replicate of PG2004.A0101
RK1001.C0101	4945393	0.3177	0.4435	Male	R1b1a2	
RK1003.C0101	463135	0.7233	0.0087	Female	-	
RK1007.A0101	509895	0.7375	0.0099	Female	-	
RK4001.A0101	2513859	0.3525	0.4741	Male	R1b1a2	
RK4002.B0101	3161001	0.3662	0.4834	Male	R1b1a2	
SA6001.A0101	6979281	0.6900	0.0144	Female	-	
SA6002.A0101	5684148	0.6912	0.0101	Female	-	
SA6003.B0101	1387568	0.3703	0.4389	Male	R1b1a2	
SA6004.A0101	5436082	0.3511	0.3897	Male	Q1a2	
SA6004.B0101	1257786	0.3462	0.4854	Male	Q1a2	independent replicate of SA6004.A0101
SA6010.A0101	1139408	0.3631	0.4484	Male	?	
SA6013.B0101	1753402	0.3723	0.4230	Male	R1	
SIJ002.A0101	5726979	0.3464	0.5820	Male	L	
SIJ003.A0101	4934680	0.7279	0.0084	Female	-	
VEK006.A0101	598647	0.7295	0.0040	Female	-	
VEK007.A0101	1552864	0.4009	0.4473	Male	J	
VEK008.A0101	34601	0.4110	0.3429	Male	?	
VEK009.A0101	1031812	0.3757	0.4305	Male	J1	
VJ1001.B0101	2719429	0.7505	0.0049	Female	-	
VJ1001.D0101	3109023	0.7380	0.0052	Female	-	independent replicate of VJ1001.B0101
ZO2002.C0101	2191544	0.7108	0.0071	Female	-	
I1722	658099	0.7132	0.0120	Female	-	
I2055	543337	0.3606	0.4647	Male	J	
I2056	4020874	0.4057	0.4768	Male	J2a	
I1720	134524	0.3870	0.4308	Male	?	
I1723	200416	0.3939	0.4823	Male	?	
I2051	337887	0.3586	0.4328	Male	J	
I6266	1974285	0.4160	0.4926	Male	J2a1	
I6267	537729	0.8006	0.0161	Female	-	
I6268	1091304	0.4058	0.4845	Male	J2a1	
I6270	15010	0.4126	0.4548	Male	?	
I6272	1513390	0.4148	0.4876	Male	G2a2a	
I2057	37124	0.7210	0.1235	undetermined		intermediate sex ratio

Y chromosome haplogroup determination

For the male individuals, we determined Y-chromosome haplogroups by identifying the most derived allele upstream and the most ancestral allele downstream in the phylogenetic tree in the ISOGG version 11.89 (accessed March 31, 2016) (http://www.isogg.org/tree). If the most derived Y chromosome SNP upstream was a C->T or G->A substitution (susceptible to ancient DNA damage), we required as least two derived SNPs to assign it to the haplogroup (otherwise, we assigned it to the upstream haplogroup).

ARM002.A0101 could be assigned as G2b based on Z8017: A->G, M3115: G->T, PF5721: G->A, and Z8020: C->T. This individual also has multiple upstream mutations for haplogroup G2: F744: G->A, PF2787: C->T, Z3100: C->G, M3469: G->T, L89: C->T, M3480: T->A, F1189: A->G, F1239: C->T, F1294: T->A, M3491: A->G, PF2912: T->G, Z6023: C->T, CTS1868: G->A, CTS4413: T->C, CTS4703: C->T, CTS10089: G->A, M3579: A->G, F3198: G->T, M3585: G->A, PF3119: G->T, F3536: C->T, Z6474: T->C.

ARM003.A0101 could be assigned as G2b based on Z8017: A->G and M3145: A->G. This individual also has multiple upstream mutations for haplogroup G2: F744: G->A, PF2787: C->T, Z3060: G->C, PF2792: A->G, PF2797: C->T, M3480: T->A, F1189: A->G, F1239: C->T, F1294: T->A, F1393: G->A, M3491: A->G, Z6023: C->T, CTS1900: T->A, CTS4136: G->A, CTS4413: T->C, F2319: A->T, CTS6742: G->C, CTS7662: C->T, CTS10089: G->A, M3579: A->G, F3198: G->T, M3585: G->A, P287: G->T, F3536: C->T, Z6474: T->C.

BU2001.A0101 could be assigned as R1b1a2a2 based on Z2105: C->A. This individual also has three upstream mutations for haplogroup R1b1a2: PF6430: T->A, PF6432: C->A, PF6438: C->T and three upstream mutations for haplogroup R1: P242: G->A, P238: G->A, P236: C->G.

GW1001.A0101 could be assigned as R1b1a2a2 based on the mutation Z2105: C->A. This individual also has three upstream mutations for haplogroup R1b1a2: PF6399: C->T, PF6430: T->A, PF6434: A->G, PF6438: C->T, L150.1: C->T, PF6475: C->A, PF6482: A->G, PF6509: A->G, CTS11468: T->G.

KBD001 could be assigned as R1b1a2 based on the mutations: PF6399:C->T, M520:T->A, L773:A->G, PF6430:T->A, L265:A->G, L483:C->T, PF6434:A->G, PF6438:C->T, L150.1:C->T, PF6475:C->A, CTS8728:C->T, L500:C->A, PF6482:A->G, PF6495:G->A, PF6497:C->G, PF6505:G->A, M269:T->C, CTS12478:G->A. We note that this individual also has a downstream mutation L23: G->A determining haplogroup R1b1a2a, but this mutation might be caused by ancient DNA damage.

KDC001.A0101 could be assigned as J2b based on the mutations: L282: C->T, M12: G->T, M221: G->A, and M314: A->C. This individual also has multiple upstream mutations for haplogroup J: CTS687:A->T, CTS852:G->A, PF4505:T->C, CTS1250:G->T, PF4513:C->T, F1167:G->A, F1168:G->A, F1181:G->C, PF4519:C->G, PF4521:T->C, PF4524:G->A, FGC1604:G->A, FGC3271:G->A, FGC1609:C->G, PF4530:C->T, F1744:G->A, F1826:G->A, CTS3872:G->A, CTS4204:G->A, CTS4349:G->A, F2114:G->A, CTS5628:C->G, CTS5934:C->T, CTS7229:C->A, F2502:G->A, S19861:T->A, CTS7738:T->C, CTS7832:A->G, F2769:G->C, F2839:C->T, CTS9877:A->G, P209:T->C, F2973:C->T, CTS10446:G->C, F3119:C->T, F4299:T->A, S22619:C->A, F4300:T->A, F3138:G->A, PF4591:C->A, F3176:T->C, PF4594:C->A, PF4595:C->A, FGC1599:A->T, Z7829:G->C, F3347:C->T, M304:A->C, CTS11291:G->T, L778:T->C, CTS11571:C->A, CTS11750:C->T, CTS12047:A->G.

LYG001.A0101 could be assigned as haplogroup R1b1a2 based on the mutations PF6399:C->T, PF6430:T->A, L265:A->G, PF6434:A->G, PF6438:C->T, L150.1:C->T, PF6475:C->A, CTS8728:C->T, PF6482:A->G, PF6494:G->A, PF6497:C->G, PF6505:G->A, PF6509:A->G.

MK5001 could be assigned as haplogroup L based on the mutations L878: A->G, M61: C->T, and L855: C->T. This individual also has multiple upstream mutations for haplogroup LT: CTS753: G->A, L298: T->C, PF5531: C->T, PF5536: C->A, CTS2888: A->G, CTS3648: C->T, CTS5175: A->G, and PF5566: G->T.

MK5004 could be assigned as haplogroup L based on the mutations L878: A->G, M185: C->T, M61: C->T, and L855: C->T. This individual also has multiple upstream mutations for haplogroup LT: CTS753: G->A, L298: T->C, PF5531: C->T, PF5536: C->A, CTS2888: A->G, CTS3648: C->T, CTS4783: T->C, and CTS5175: A->G.

MK5009.A0101 could be assigned as R1b1a2 based on the mutations L773: A->G, L482: G->A, PF6432: C->A, PF6434: A->G, L150.1: C->T, PF6475: C->A, PF6494: G->A, PF6495: G->A, and PF6509: A->G.

NV3001 could be assigned as Q1a2 based on the mutations M346: C->G, L56: G->A, and CTS2656: C->T. This individual also has multiple upstream mutations for haplogroup Q1a: F1426: C->T, FGC8413: A->T, FGC8415: G->T, CTS2006: C->T, CTS4793: C->G, and CTS5301: G->A and haplogroup Q1: P36.2: G->T, L232: G->A, and L274: A->G.

OSS002.B0101 could be assigned as haplogroup J based on the mutations F1181: G->C, CTS5934: C->T, and F3119: C->T.

PG2001 could be assigned as haplogroup R1b1 based on the mutation M415: C->A. This individual also has one upstream mutation for haplogroup R1b: M343: C->A and multiple upstream mutations for haplogroup R1: P294: G->C, P231: A->G, P225: G->T, P286: C->T, and M306: C->A.

PG2004 could be assigned as haplogroup R1b1 based on the mutation L278: C->T (we note that this mutation might be caused by ancient DNA damage). This individual also has one upstream mutation for haplogroup R1b: M343: C->A and multiple upstream mutations for haplogroup R1: P294: G->C, P242: G->A, P231: A->G, P286: C->T.

RK1001.C0101 could be assigned as haplogroup R1b1a2 based on the mutations L773: A->G, PF6430: T->A, L265: A->G, PF6434: A->G, PF6438: C->T, L150.1: C->T, CTS3575: C->G, PF6482: A->G, and PF6495: G->A.

RK4001.A0101 could be assigned as haplogroup R1b1a2 based on the mutations PF6434: A->G, PF6438: C->T, L150.1: C->T, PF6475: C->A, PF6482: A->G, PF6497: C->G, PF6505: G->A, and CTS12478: G->A.

RK4002.B0101 could be assigned as haplogroup R1b1a2 based on the mutations M520: T->A, PF6430: T->A, L265: A->G, PF6434: A->G, PF6438: C->T, PF6475: C->A, CTS8728: C->T, PF6482: A->G, PF6494: G->A, PF6495: G->A, PF6497: C->G

SA6003.B0101 could be assigned as haplogroup R1b1a2 based on the mutations L265: A->G, PF6434: A->G, L150.1: C->T, PF6482: A->G, and PF6495: G->A.

SA6004 could be assigned as haplogroup Q1a2 based on the mutations M346: C->G, L56: G->A, and CTS2656: C->T. This individual also has one upstream mutation for haplogroup Q1a: CTS97: T->A, F903: G->C, F1304: G->T, FGC8415: G->T, CTS1845: G->C, CTS2006: C->T, CTS4793: C->G, CTS5804: G->A, CTS7611: A->T, M1115: G->A, M1158: C->T, M1168: G->A, M1169: G->A and haplogroup Q1: P36.2: G->T, L232: G->A.

SA6013.B0101 could be assigned as haplogroup R1 based on the mutations P294: G->C, P225: G->T, P236: C->G, and M306: C->A.

SIJ002.A0101 could be assigned as haplogroup L based on the mutations L863: T->C, L878: A->G, M11: A->G, M61: C->T, L855: C->T. This individual also has multiple upstream mutations for haplogroup LT: CTS753: G->A, L298: T->C, PF5531: C->T, PF5536: C->A, CTS2888: A->G, and CTS5175: A->G.

VEK007.A0101 could be assigned as haplogroup J based on the mutations CTS687: A->T, PF4505: T->C, F1167: G->A, FGC3271: G->A, L60: C->T, CTS4204: G->A, F2116: C->G, CTS5628: C->G, CTS6958: G->A, CTS7738: T->C, PF4575: A->G, F2839: C->T, P209: T->C, F2973: C->T, F3138: G->A, PF4595: C->A, YSC0000228: G->T, CTS10858: G->A, CTS11291: G->T, and CTS11571: C->A.

VEK009.A0101 could be assigned as haplogroup J1 based on the mutation L255: A->C. This individual also has multiple upstream mutations for haplogroup J: CTS852: G->A, CTS1250: G->T, PF4513: C->T, CTS2769: T->A, CTS3732: A->G, CTS4349: G->A, CTS7229: C->A, F2502: G->A, CTS7738: T->C, F4299: T->A, S22619: C->A, F4300: T->A, F3176: T->C, and CTS11571: C->A.
I2055 could be assigned as haplogroup J based on the mutations PF4513: C->T, L60: C->T, CTS2769: T->A, F2839: C->T, PF4595: C->A, L778: T->C, CTS11787: G->A, PF4513: C->T, L60: C->T, CTS2769: T->A, F2839: C->T, PF4595: C->A, L778: T->C, and CTS11787: G->A.

I2056 could be assigned as haplogroup J2a based on the mutations L559: A->G and L152: C->T. This individual also has multiple upstream mutations for haplogroup J: CTS687: A->T, CTS852: G->A, PF4505: T->C, CTS1250: G->T, PF4513: C->T, PF4515: G->A, F1167: G->A, F1168: G->A, PF4519: C->G, F1381: G->T, PF4521: T->C, PF4524: G->A, FGC3271: G->A, PF4530: C->T, CTS2042: T->A, L60: C->T, F1826: G->A, CTS3732: A->G, CTS4204: G->A, F2116: C->G, CTS5628: C->G, CTS5678: A->T, CTS7229: C->A, F2502: G->A, CTS7832: A->G, PF4572: A->G, PF4575: A->G, F2769: G->C, CTS8974: A->G, F2817: C->T, F2839: C->T, CTS9533: C->A, P209: T->C, CTS10446: G->C, F3119: C->T, F4299: T->A, S22619: C->A, F4300: T->A, PF4591: C->A, PF4595: C->A, FGC1599: A->T, PF4598: A->G, F3347: C->T, M304: A->C, CTS11571: C->A, CTS11750: C->T, CTS11787: G->A, and CTS12047: A->G.

I2051 could be assigned as haplogroup J based on the mutations F1167:G->A, FGC1604:G->A, CTS4349:G->A, PF4567:A->C, F2746:G->A, F2769:G->C.

I6266 could be assigned as haplogroup J2a1 based on the mutations L26: T->C and F4326: A->G. This individual also has one upstream mutation M410:A->G defining haplogroup J2a and mutiple upstream mutations for haplogroup J: PF4513:C->T, F1167:G->A, F1181:G->C, PF4519:C->G, PF4521:T->C, PF4524:G->A, FGC1604:G->A, FGC3271:G->A, PF4530:C->T, F1744:G->A, CTS2769:T->A, CTS4204:G->A, F2114:G->A, F2116:C->G, CTS5628:C-

>G, CTS5934:C->T, CTS6958:G->A, CTS7028:T->C, CTS7229:C->A, CTS7483:T->A, PF4567:A->C, CTS7738:T->C, PF4575:A->G, CTS8974:A->G, CTS9533:C->A, P209:T->C, CTS10446:G->C, F3119:C->T, PF4591:C->A, F3176:T->C, PF4594:C->A, CTS10858:G->A, CTS11291:G->T, CTS12047:A->G.

I6268 could be assigned as haplogroup J2a1 based on the mutation L26:T->C. This individual also has mutiple upstream mutations for haplogroup J: CTS687:A->T, F1181:G->C, PF4519:C->G, PF4524:G->A, FGC3271:G->A, F1744:G->A, CTS2769:T->A, CTS3936:G->A, CTS4204:G->A, CTS5678:A->T, CTS7028:T->C, F2502:G->A, CTS7832:A->G, F2839:C->T, CTS9533:C->A, F3119:C->T, CTS11291:G->T, CTS11765:A->T.

I6272 could be assigned as haplogroup G2a2a based on mutations PF3165: C->A and PF3184:C->T. This individual also has mutiple upstream mutations for haplogroup G2a: F4086:C->T, Z3240:A->G, F2301:G->A, F2529:A->G, CTS11463:G->A

We were not able to determine the haplogroups of the following samples due to low coverages: IV3002.A0101, MK5007.B0101, MK5008.B0101, MK5012.A0101, SA6010.A0101, VEK008.A0101, I1720, I1723, and I6270.

Supplementary Note 5

Dating gene flow from Caucasus mountain groups into Steppe Maykop

In this section, we estimated admixture time for the observed farmer related ancestry in Steppe Maykop outlier individuals using the linkage disequilibrium (LD)-based admixture inference implemented in ALDER[54]. We computed weighted LD curves with Steppe Maykop outlier as the test population and Steppe Maykop and Kura_Araxes as references. The average admixture time for Steppe Maykop outlier is about 20 generations ago (about 580 years ago assuming 29 years per generation[55]). We caution that the date estimates might not reflect the initial farmer related admixture in Steppe Maykop outliers; instead, it is an average date of population mixture. If the admixture did not happen immediately when two populations met, or occurred many times over an extended period, the true start of mixture would be more ancient.

```
---- fit on data from 0.30 to 50.00 cM (using inter-chrom affine term) ----
d>0.30    decay:      23.34 +/- 70.67       z = 0.33
d>0.30    amp_tot:  0.00012067 +/- 0.00016094
d>0.30    amp_exp:  0.00011854 +/- 0.00016048   z = 0.74
d>0.30    amp_aff:  0.00000427 +/- 0.00000419
---- fit on data from 0.40 to 50.00 cM (using inter-chrom affine term) ----
d>0.40    decay:      21.04 +/- 9.72        z = 2.17 *
d>0.40    amp_tot:  0.00010361 +/- 0.00006259
d>0.40    amp_exp:  0.00010147 +/- 0.00006192   z = 1.64
d>0.40    amp_aff:  0.00000427 +/- 0.00000419
---- fit on data from 0.50 to 50.00 cM (using inter-chrom affine term) ----
d>0.50    decay:      19.83 +/- 8.26        z = 2.40 *
d>0.50    amp_tot:  0.00009353 +/- 0.00005701
d>0.50    amp_exp:  0.00009139 +/- 0.00005643   z = 1.62
d>0.50    amp_aff:  0.00000427 +/- 0.00000419
---- fit on data from 0.60 to 50.00 cM (using inter-chrom affine term) ----
d>0.60    decay:      21.22 +/- 9.69        z = 2.19 *
d>0.60    amp_tot:  0.00010532 +/- 0.00006435
d>0.60    amp_exp:  0.00010319 +/- 0.00006376   z = 1.62
d>0.60    amp_aff:  0.00000427 +/- 0.00000419
---- fit on data from 0.70 to 50.00 cM (using inter-chrom affine term) ----
d>0.70    decay:      22.82 +/- 94.81       z = 0.24
d>0.70    amp_tot:  0.00011856 +/- 0.00032577
d>0.70    amp_exp:  0.00011643 +/- 0.00032553   z = 0.36
d>0.70    amp_aff:  0.00000427 +/- 0.00000419
```

Supplementary Note 6
Admixture Graph modelling

In previous analyses, we defined two genetic clusters within the Greater Caucasus region, correlating to the *Caucasus* groups of the northern foothills and *Steppe* groups of the bordering steppe regions. In this section, we use the method *qpGraph*[56], as implemented in ADMIXTOOLS[57] to test models of possible phylogenetic relationships for the two genetic clusters with reported representatives of ancient West Eurasians. *qpGraph* assesses the fit of Admixture Graph models to data by computing f_2-, f_3- and f_4- statistics measuring allele sharing among pairs, triples, and quadruples of populations and evaluating fits based on the maximum |Z|-score comparing predicted and observed values of these statistics. We use the newly generated Maykop and Yamnaya_Caucasus data to represent the above two clusters, respectively. We also add Eneolithic_Steppe into the graph as a potential source for the Steppe groups.
We started with a skeleton phylogenetic tree consisting of Mbuti, Loschbour, and MA1 without admixture (Supplementary Figure 11), which could be fitted in the graph without outliers:

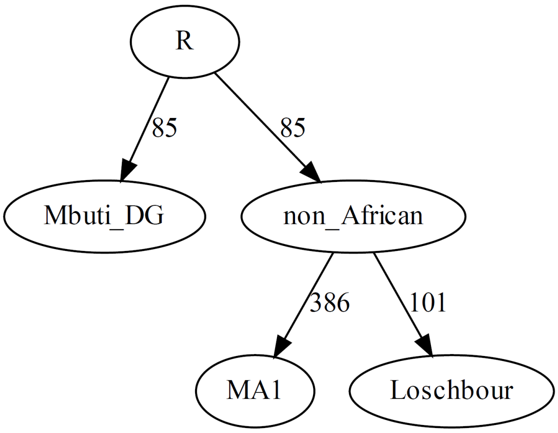

Supplementary Figure 11. Skeleton Admixture Graph without admixture edges.
Fit to genetic data with no *f*-statistics more than |Z|>3 different between model and expectation. The Z-score of the worst *f*-statistic (Mbuti, MA1; MA1, Loschbour) = -0.078. The drift along edges (number next to the arrows) is multiplied by 1000.

We then added Globular_Amphora as a representative of European farming populations to all possible edges in the graph of Supplementary Figure 11. We obtained one tree topology that fitted with no outliers and one additional admixture event implying that Globular_Amphora derived ancestry from Loschbour and a basal lineage branching off earlier than the split of MA1 and Loschbour (Supplementary Figure 12), which is consistent with a previous study that reported that Early European Farmers (EEF) had basal Eurasian ancestry[58].

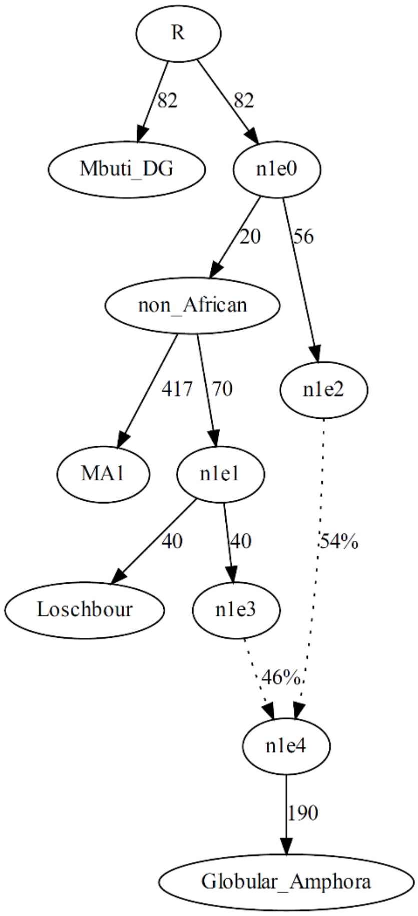

Supplementary Figure 12. Adding Globular_Amphora.
The model in which Globular_Amphora is admixed, which is a fit to the data in the sense that there are no *f*-statistics more than |Z|>3 different between model and expectation. The Z-score of the worst *f*-statistic (MA1, Loschbour; MA1, Globular_Amphora) = 0.075.

We continued to add EHG into the graph in Supplementary Figure 12 but failed to get fitted models with no additional admixture event. We got four fitted models with one additional admixture event:
b0.c1: the Z-score of the worst *f*-statistic = 0.051, likelihood score = 16.138 (Supplementary Figure 13);
b0.c3: the Z-score of the worst *f*-statistic = 0.170, likelihood score = 80.896;
b0.c5: the Z-score of the worst *f*-statistic = 0.128, likelihood score = 48.025;
b0.c6: the Z-score of the worst *f*-statistic = 1.829, likelihood score = 9027.475;

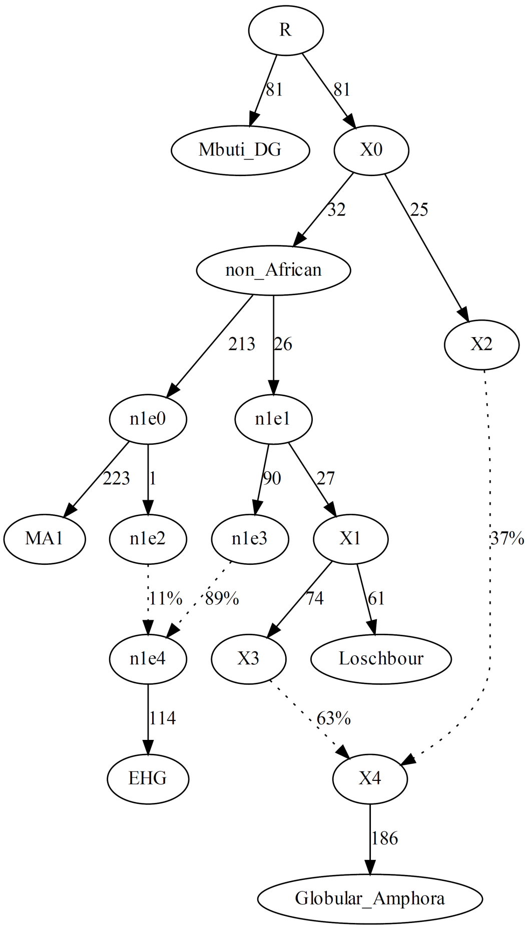

Supplementary Figure 13. Adding EHG.
The model b0.c1 in which EHG are admixed deriving ancestry from MA1 and Loschbour related lineages, which is a fit to the data in the sense that there are no *f*-statistics more than |Z|>3 different between model and expectation. The Z-score of the worst *f*-statistic (Mbuti, Globular_Amphora; Loschbour, Globular_Amphora) = 0.051.

We then added CHG into the above 3 graphs b0.c1, b0.c3, and b0.c5 with low Z-scores and likelihood scores. We were not able to fit CHG into the graphs without invoking an additional admixture event, but got 17 fitted models with one additional admixture event:
b0.c1-c2.c5: the Z-score of the worst *f*-statistic = 2.971, likelihood score = 13592.506;
b0.c1-c2.c6: the Z-score of the worst f-statistic = 2.849, likelihood score = 13505.251;
b0.c1-c4.d1: the Z-score of the worst *f*-statistic = 1.264, likelihood score = 2462.240;
b0.c1-c4.d3: the Z-score of the worst *f*-statistic = -2.290, likelihood score = 11176.961
b0.c1-c4.d5: the Z-score of the worst *f*-statistic = -2.744, likelihood score = 9730.920;
b0.c1-c4.d6: the Z-score of the worst *f*-statistic = 2.600, likelihood score = 12183.066;
b0.c3-c1.c4: the Z-score of the worst *f*-statistic = 2.879, likelihood score = 13619.849;
b0.c3-c2.c5: the Z-score of the worst *f*-statistic = 2.883, likelihood score = 13534.971;
b0.c3-c2.c6: the Z-score of the worst *f*-statistic = 2.866, likelihood score = 13543.098;
b0.c3-c4.d5: the Z-score of the worst *f*-statistic = 2.942, likelihood score = 16173.414;
b0.c3-c4.d6: the Z-score of the worst *f*-statistic = 2.559, likelihood score = 11034.631;
b0.c5-c1.c4: the Z-score of the worst *f*-statistic = 2.852, likelihood score = 13585.957;
b0.c5-c2.c6: the Z-score of the worst *f*-statistic = 2.850, likelihood score = 13525.020;
b0.c5-c2.d3: the Z-score of the worst *f*-statistic = 2.956, likelihood score = 13657.019;
b0.c5-c4.d4: the Z-score of the worst *f*-statistic = 2.890, likelihood score = 39766.289;

b0.c5-c4.d5: the Z-score of the worst *f*-statistic = -2.506, likelihood score = 13022.425;
b0.c5-c4.d6: the Z-score of the worst *f*-statistic = 2.575, likelihood score = 12207.553;

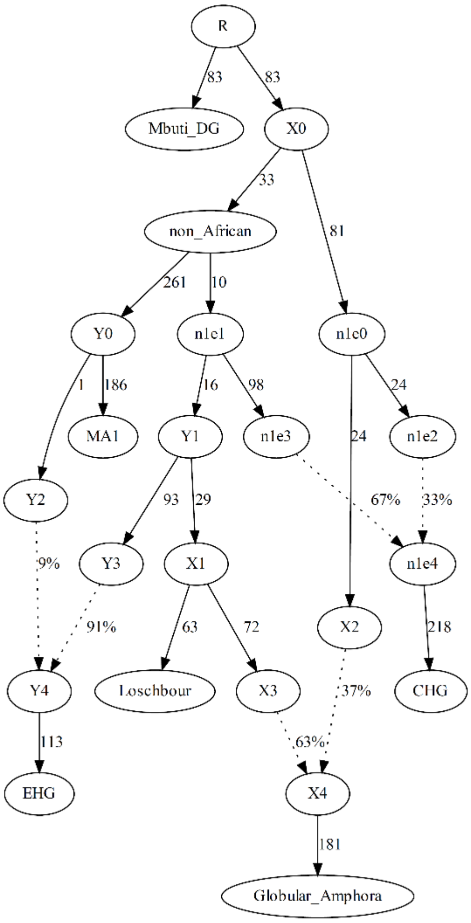

Supplementary Figure 14. Adding CHG.
The model b0.c1-c4.d1, in which CHG are admixed deriving ancestry from basal Eurasian and Loschbour related lineages, which is a fit to the data in the sense that there are no f-statistics more than |Z|>3 different between model and expectation. The Z-score of the worst *f*-statistic (Mbuti, CHG; Loschbour, EHG) = 1.264.

We continued to add Eneolithic_Steppe onto the model b0.c1-c4.d1 (Figure SF3.4) because this model has the lowest Z-score and likelihood score. We obtained only one graph topology fitted with no outliers, which models Eneolithic_Steppe as an admixture with one source of ancestry EHG-related, and the other lineage from the basal Eurasian side that also contributed to CHG (Supplementary Figure 15).

We continued to add Yamnaya_Caucasus into the graph in Supplementary Figure 15 and got two fitted models with one additional admixture event:
d6.e4-c5.f6: the Z-score of the worst f-statistic = -2.823, likelihood score = 13469.860;
d6.e4-c6.f6: the Z-score of the worst f-statistic = -2.044, likelihood score = 7733.921 (Supplementary Figure 16).
We continued to add Maykop into the above two models but failed to get fitted models with no additional admixture event or with only one additional admixture event. The best model is shown in Supplementary Figure 17, suggesting that Maykop derives ancestry from CHG and Globular_Amphora related lineages, but it is not a good fit to the data in the sense that there is

one *f*-statistics outlier: the Z-score of *f* (MA1, Maykop; EHG, Eneolithic_Steppe) = -3.369. The outlier could be interpreted as suggesting unmodeled affinity between the pair of MA1 and Eneolithic_Steppe or Maykop and EHG. We then manually added an admixture event from MA1 to Eneolithic_Steppe in the graph in Supplementary Figure 18 and an admixture event from EHG to Maykop in the graph in Supplementary Figure 19.

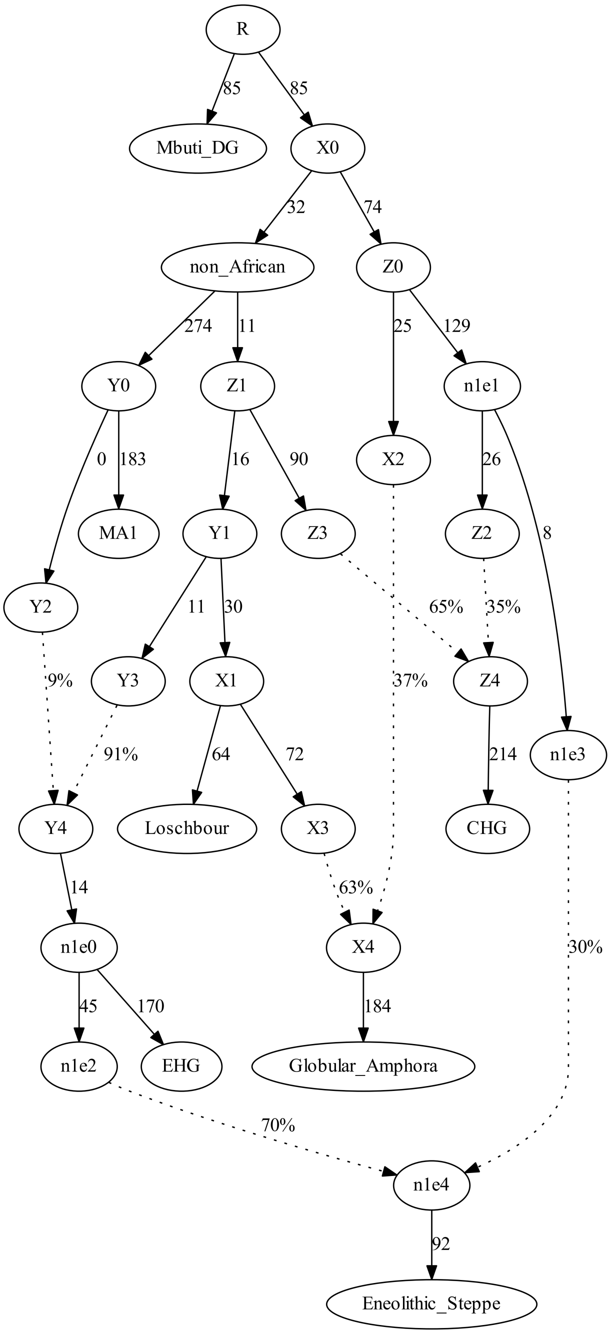

Supplementary Figure 15. Adding Eneolithic_Steppe.
The model in which Eneolithic_Steppe are admixed deriving ancestry from CHG related basal Eurasian and EHG related lineages, which is a fit to the data in the sense that there are no f-statistics more than |Z|>3 different between model and expectation. The Z-score of the worst *f*-statistic (MA1, Loschbour; EHG, Eneolithic_Steppe) = -1.815.

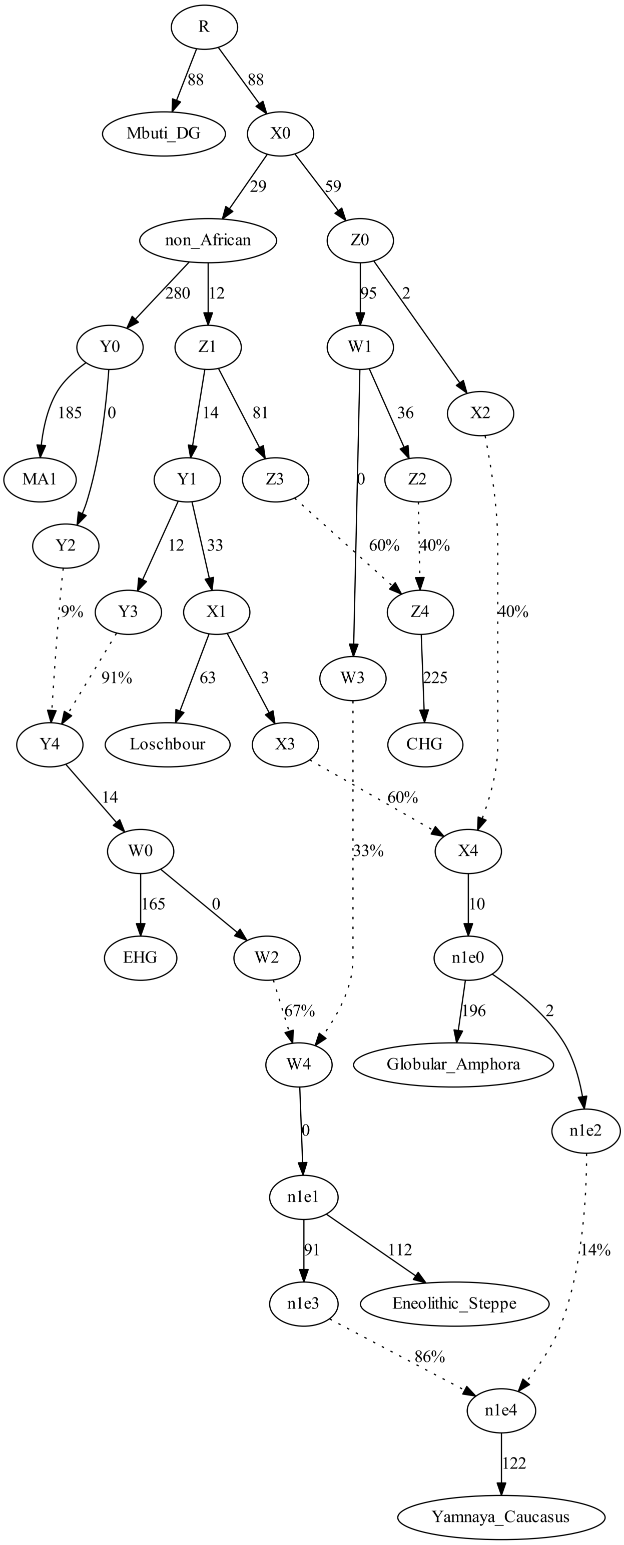

Supplementary Figure 16. Adding Yamnaya_Caucasus.
The model d6.e4-c6.f6, in which Yamnaya_Caucasus are admixed deriving ancestry from Eneolithic_Steppe and Globular_Amphora related lineages, which is a fit to the data in the sense that there are no f-statistics more than |Z|>3 different between model and expectation. The Z-score of the worst *f*-statistic (MA1, Loschbour; EHG, Eneolithic_Steppe) = -2.044.

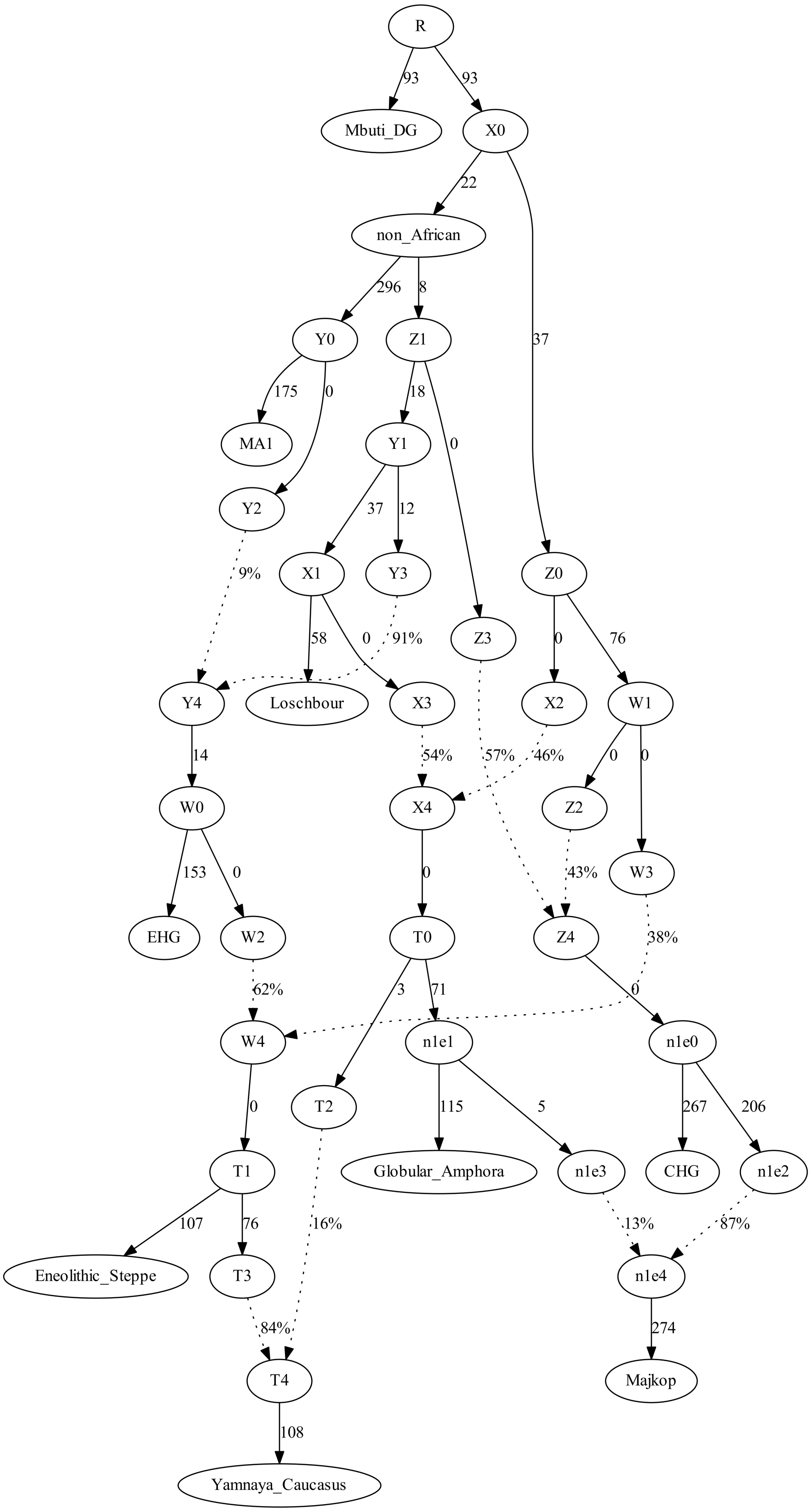

Supplementary Figure 17. Adding Maykop.

The best model by fitting Maykop deriving ancestry from CHG and Globular_Amphora related lineages, which is not a fit to the data in the sense that there is one *f*-statistic outlier: the Z-score of *f*(MA1, Maykop; EHG, Eneolithic_Steppe) = -3.369.

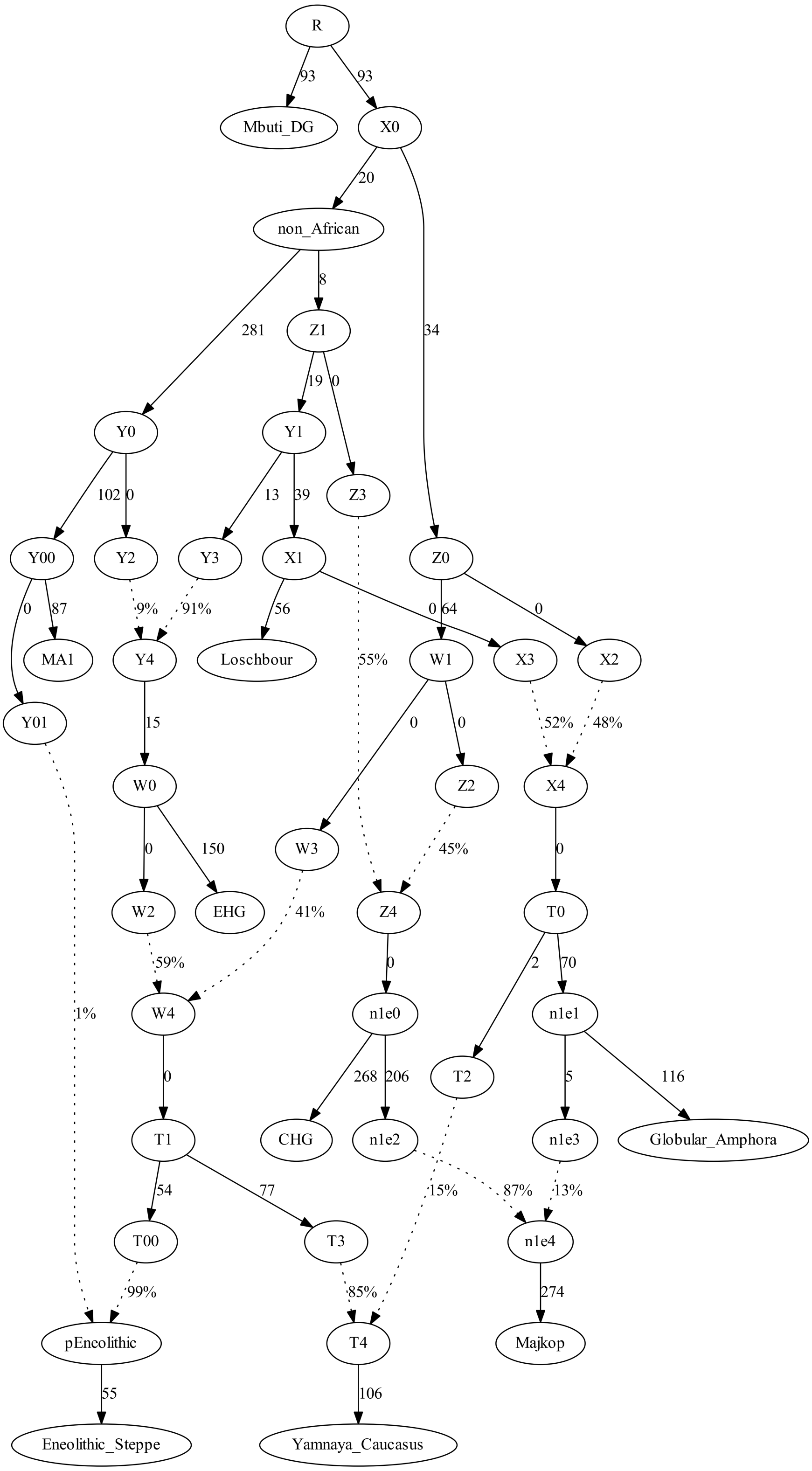

Supplementary Figure 18. MA1-related admixture into Eneolithic_Steppe.
We added an admixture event from MA1 related lineage to Eneolithic_Steppe. The Z-score of the worst *f*-statistic (CHG, Yamnaya_Caucasus; Eneolithic_Steppe, Yamnaya_Caucasus) = 2.945. The likelihood score for this model = 17016.725.

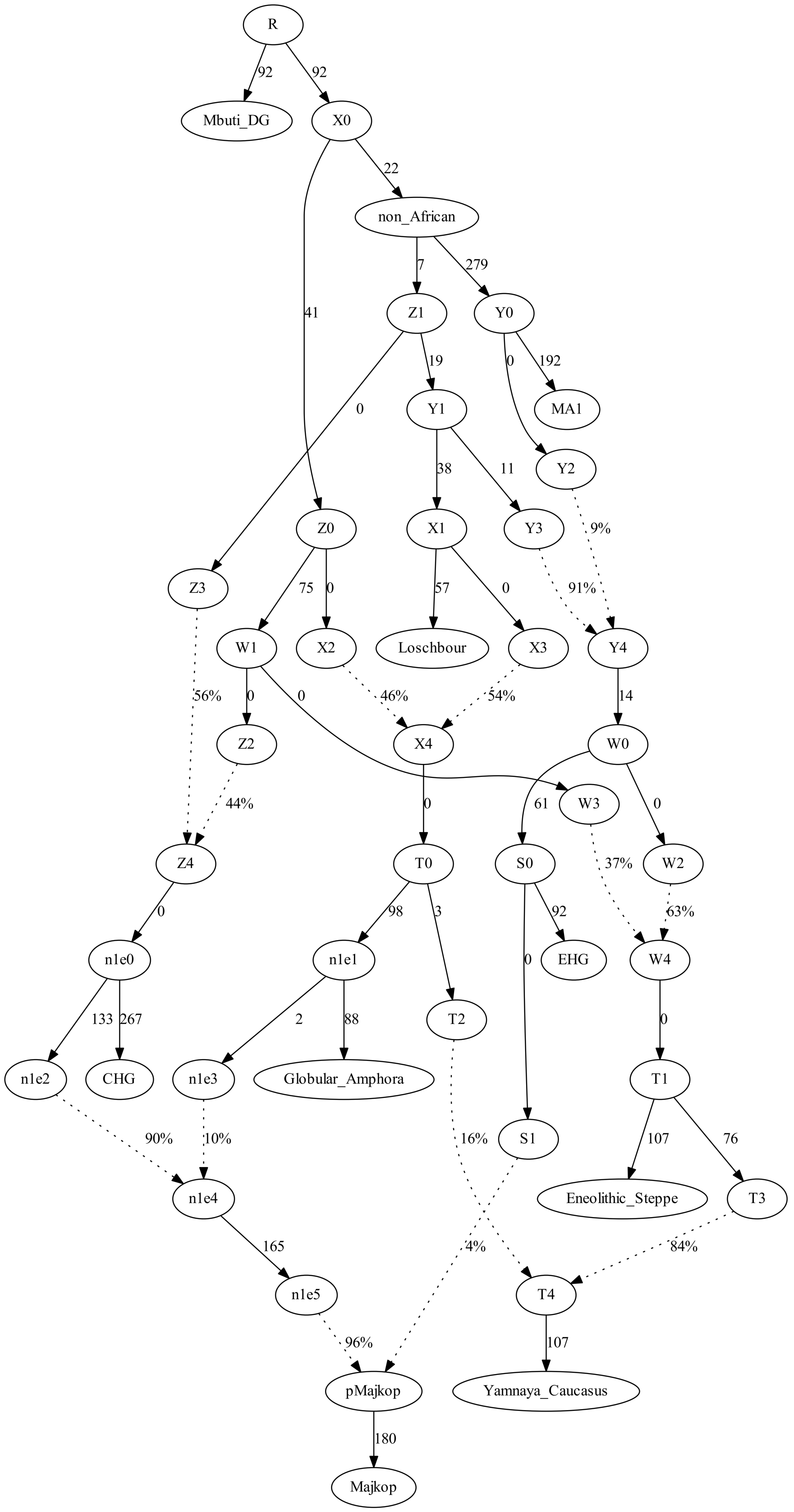

Supplementary Figure 19. EHG-related admixture into Maykop.
We added an admixture event from EHG related lineage to Maykop. The Z-score of the worst *f*-statistic (CHG, Yamnaya_Caucasus; Eneolithic_Steppe, Yamnaya_Caucasus) = 2.824. The likelihood score for this model = 15240.753.

Supplementary Note 7

Phenotypic variants and marker under selection

We also explored a set of SNPs that has been shown to be under selection in West Eurasia over the last 8000 years of human history[59] (Supplementary Table 24). We observe an almost complete lack of the derived allele at the LCT locus, which codes for lactase persistence, i.e. the ability to digest milk sugar in adulthood. Only one 4000-year-old individual from the Late North Caucasus site of Kabardinka is heterozygous for the derived allele. The SNPs at *SLC24A5* and *SLC45A2* are associated with lighter skin pigmentation and HERC2 with lighter eye colour. The great majority of individuals from both main clusters likely had lighter skin colour, while the eye colour varied within each cluster. Interestingly, one of the Steppe Maykop individuals carried the derived allele at the *EDAR* 370A locus, which was shown to be at high frequency in East Asia thus associated with straighter, thicker hair and shovel-shaped incisors. This finding is consistent with the ancestral East Asian/Siberian affinity shown above.

Supplementary Table 24. Allele information on phenotypic SNPs that thought to be affected by selection. Only high-quality (q>30) bases were counted. rs4988235 on the LCT gene is responsible for lactase persistence in Europe. The SNPs at *SLC24A5* and *SLC45A2* are responsible for light skin pigmentation. The SNP at *EDAR* affects tooth morphology and hair thickness. The SNP at *HERC2* is the primary determinant of light eye colour in present-day Europeans. We highlight in light blue sites that are likely to be heterozygous, and in red sites that are likely to be homozygous for the derived allele.

**Population**	**Sample ID**	**Gene**<br>**SNP**	***LCT***<br>**rs4988235**	***SLC45A2***<br>**rs16891982**	***SLC24A5***<br>**rs1426654**	***EDAR***<br>**rs3827760**	***HERC2***<br>**rs12913832**
		**Ancestral**<br>**Derived**	**G**<br>**A**	**C**<br>**G**	**G**<br>**A**	**A**<br>**G**	**A**<br>**G**
Eneolithic Caucasus	I2056	Coverage	4	8	2	8	5
		Derived allele frequency	0%	0%	100%	0%	20%
Eneolithic Caucasus	I2055	Coverage	0	1	0	1	0
		Derived allele frequency	n/a	100%	n/a	0%	n/a
Maykop	OSS001	Coverage	1	1	1	2	2
		Derived allele frequency	0%	100%	100%	0%	0%
Maykop	MK5007	Coverage	0	0	0	0	0
		Derived allele frequency	n/a	n/a	n/a	n/a	n/a
Maykop Novosvobodnaya	I6266	Coverage	5	5	1	0	1
		Derived allele frequency	0%	0%	100%	n/a	0%
Maykop Novosvobodnaya	I6268	Coverage	2	2	1	1	1
		Derived allele frequency	0%	0%	100%	0%	100%
Maykop Novosvobodnaya	I6272	Coverage	1	1	0	1	1
		Derived allele frequency	0%	0%	n/a	0%	0%
Late Maykop	SIJ002	Coverage	17	14	0	10	2
		Derived allele frequency	0%	14.3%	n/a	0%	0%
Late Maykop	SIJ003	Coverage	6	2	2	9	0
		Derived allele frequency	0%	0%	100%	0%	n/a
Late Maykop	MK5001	Coverage	8	11	3	9	6
		Derived allele frequency	0%	0%	100%	0%	0%
Late Maykop	MK5004	Coverage	23	9	1	12	3
		Derived allele frequency	0%	0%	100%	0%	0%
Late Maykop	SA6002	Coverage	20	7	2	15	6
		Derived allele frequency	0%	42.9%	100%	0%	100%
Kura-Araxes	VEK006	Coverage	1	1	0	1	0
		Derived allele frequency	0%	100%	n/a	0%	n/a
Kura-Araxes	VEK007	Coverage	0	0	2	0	0
		Derived allele frequency	n/a	n/a	100%	n/a	n/a
Kura-Araxes	VEK009	Coverage	2	1	1	1	0
		Derived allele frequency	0%	100%	100%	0%	n/a
Kura-Araxes	ARM001	Coverage	4	4	0	4	1
		Derived allele frequency	0%	25%	n/a	0%	0%
Kura-Araxes	ARM002	Coverage	3	6	0	10	0
		Derived allele frequency	0%	0%	n/a	0%	n/a
Kura-Araxes	ARM003	Coverage	7	8	0	6	7
		Derived allele frequency	0%	0%	n/a	0%	0%
Kura-Araxes	SC30_L532	Coverage	1	0	0	0	1
		Derived allele frequency	0%	n/a	n/a	n/a	100%
MBA North Caucasus	KDC001	Coverage	21	13	0	6	0
		Derived allele frequency	0%	0%	n/a	0%	n/a
		Coverage	8	9	2	12	5

MBA North Caucasus	KDC002	Derived allele frequency	0%	0%	100%	0%	0%
Dolmen LBA	I2051	Coverage	1	0	0	1	0
		Derived allele frequency	0%	n/a	n/a	0%	n/a
Eneolithic steppe	VJ1001	Coverage	10	5	1	6	4
		Derived allele frequency	0%	0%	100%	0%	0%
Eneolithic steppe	PG2001	Coverage	20	18	1	8	3
		Derived allele frequency	0%	61.1%	100%	0%	0%
Eneolithic steppe	PG2004	Coverage	19	10	0	9	14
		Derived allele frequency	0%	0%	n/a	0%	0%
Steppe Maykop	SA6004	Coverage	8	11	1	9	6
		Derived allele frequency	0%	0%	100%	0%	0%
Steppe Maykop	SA6001	Coverage	25	14	0	18	2
		Derived allele frequency	0%	0%	n/a	61.1%	0%
Steppe Maykop	AY2001	Coverage	16	14	1	13	5
		Derived allele frequency	0%	0%	100%	0%	40%
Steppe Maykop	AY2003	Coverage	16	11	0	11	4
		Derived allele frequency	0%	54.5%	n/a	0%	50%
Steppe Maykop	MK5005	Coverage	1	1	0	0	0
		Derived allele frequency	0%	0%	n/a	n/a	n/a
Steppe Maykop outlier	SA6013	Coverage	1	1	3	3	1
		Derived allele frequency	0%	0%	100%	0%	0%
Steppe Maykop outlier	IV3002	Coverage	0	2	0	0	0
		Derived allele frequency	n/a	0%	n/a	n/a	n/a
Yamnaya Caucasus	ZO2002	Coverage	2	2	1	2	7
		Derived allele frequency	0%	50%	100%	0%	42.9%
Yamnaya Caucasus	SA6010	Coverage	1	1	0	1	0
		Derived allele frequency	0%	0%	n/a	0%	n/a
Yamnaya Caucasus	RK1001	Coverage	5	6	3	14	0
		Derived allele frequency	0%	100%	100%	0%	n/a
Yamnaya Caucasus	RK1007	Coverage	2	0	0	2	0
		Derived allele frequency	0%	n/a	n/a	0%	n/a
North Caucasus	RK1003	Coverage	1	0	1	3	0
		Derived allele frequency	0%	n/a	100%	0%	n/a
North Caucasus	MK5009	Coverage	1	2	2	1	1
		Derived allele frequency	0%	100%	100%	0%	0%
North Caucasus	PG2002	Coverage	0	0	1	3	0
		Derived allele frequency	n/a	n/a	100%	0%	n/a
North Caucasus	LYG001	Coverage	4	6	1	8	0
		Derived allele frequency	0%	0%	100%	0%	n/a
North Caucasus	BU2001	Coverage	0	2	1	0	1
		Derived allele frequency	n/a	50%	100%	n/a	0%
North Caucasus	GW1001	Coverage	7	4	1	11	1
		Derived allele frequency	0%	25%	100%	0%	0%
Catacomb	MK3003	Coverage	5	1	0	3	0
		Derived allele frequency	0%	0%	n/a	0%	n/a
Catacomb	RK4001	Coverage	1	8	0	2	4
		Derived allele frequency	0%	0%	n/a	0%	0%
Catacomb	RK4002	Coverage	8	6	3	1	1
		Derived allele frequency	0%	100%	100%	0%	0%
Catacomb	SA6003	Coverage	3	4	0	1	0
		Derived allele frequency	0%	0%	n/a	0%	n/a
Late North Caucasus	KBD001	Coverage	29	21	3	22	12
		Derived allele frequency	0%	0%	100%	0%	41.7%
Late North Caucasus	KBD002	Coverage	20	15	3	14	1
		Derived allele frequency	35%	53.3%	100%	0%	0%
Lola	NV3001	Coverage	22	15	2	11	8
		Derived allele frequency	0%	66.7%	100%	0%	0%

Supplementary References

1. Rezepkin AD. *Das frühbronzezeitliche Gräberfeld von Klady und die Majkop-Kultur in Nordwestkaukasien*. (Leidorf, Rahden Westf., 2000).

2. Dorow W. Altes Grab eines Heerführers unter Attila entdeckt am 18. April 1750 bei Merseburg. (eds), Halle, 1832).

3. Bohn U, Neuhäusel R. Karte der natürlichen Vegetation Europas / Map of the Natural Vegetation of Europe: Maßstab / Scale 1 : 2500000. (eds). (Landwirtschaftsverlag, Münster, 2000).

4. Spengler RN, Frachetti MD, Fritz GJ. Ecotopes and Herd Foraging Practices In the Steppe/Mountain Ecotone of Central Asia During the Bronze and Iron Ages. *Journal of Ethnobiology* **33**, 125–147 (2013).

5. Shishlina N. *Reconstruction of the Bronze Age of the Caspian steppes: Life styles and life ways of pastoral nomads*. (Archaeopress [u.a.], Oxford, 2008).

6. Sagona A. *The Archaeology of the Caucasus*. (Cambridge University Press, Cambridge, 2017).

7. Kohl P, Trifonov V. The Prehistory of the Caucasus: Internal Developments and External Interactions. In: *The Cambridge World Prehistory* (eds Renfrew C, Bahn P). (Cambridge University Press, Cambridge, 2014).

8. Reinhold S*, et al.* Contextualising innovation. About cattle owners and wagon drivers in the North Caucasus and beyond. In: *Appropriating Innovation. Entangeled knowledge in Eurasia, 5000-1500 BCE* (eds Maran J, Stockhammer P). (Oxbow Books, Oxford; Philadelphia, 2017).

9. Trifonov V. Sushchestvoval li na Severo-Zapadnom Kavkaze neolit? In: *Adaptaciya kultur paleolita - eneolita k izmeniyam prirodnoy sredy na Severo-Zapadnom Kavkaze* (eds Trifonov VA). (Institut Istorii Materielnoy Kultury RAN, Sankt Petersburg, 2009).

10. Kohl PL, Magomedov RG. Early Bronze developments on the West Caspian Coastal Plain. *Paléorient* **40**, 93–114 (2014).

11. Knipper C, Reinhold S, Gresky J, Belinskiy A, Alt KW. Economic strategies at Bronze Age and Early Iron Age upland sites in the North Caucasus: Archaeological and stable isotope investigations. In: *Isotopic investigations of pastoralism in prehistory* (eds Miller ARV, Makarewicz C). (Routledge, London, 2018).

12. Korenevskiy SN. *Rozhdenie kurgana: pogrebalnye pamjatniki eneoliticheskogo vremeni Predkavkazja i Volgo-Donskogo mezhdurechia*. (TAUS, Moscow, 2012).

13. Mimokhod RA. *Lolinskaja Kultura: severo-zapadny Prikaspiy na rubezhe srednego i pozdnego periodov bronzovogo veka*. (Institute Archeologii RAN, Moscow, 2013).

14. Kohl PL. *The Making of Bronze Age Eurasia*. (Cambridge University Press, Cambridge, 2007).

15. Hansen S. The 4th millennium: a watershed in European prehistory. In: *Western Anatolia Before Troy. Proto Urbanisation in the 4th Millenium BC?* (eds Horejs B, Mehofer M). (Verlag der Österreichischen Akademie der Wissenschaften, Vienna, 2014).

16. Helwing B*, et al.* The Kura projects: New research on the later prehistory of the southern Caucasus. In: *Archäologie in Iran und Turan* (eds). (Dietrich Reimer Verlag, Berlin, 2017).

17. Gorelik A. Zu kaukasischen und vorderasiatischen Einflüssen bei der Neolithisierung im unteren Donbecken. *Eurasia Antiqua* **20**, 143-170 (2014 [2017]).

18. Govedarica B. *Zepterträger, Herrscher der Steppen: Die frühen Ockergräber des älteren Äneolithikums im karpatenbalkanischen Gebiet und im Steppenraum Südost- und Osteuropas*. (von Zabern, Mainz am Rhein, 2004).

19. Lyonnet B. *Les cultures du Caucase: (VIe - IIIe millénaires avant notre ère) : leurs relations avec le Proche-Orient*. (Éd. Recherche sur les Civilisations, Paris, 2007).

20. Shishlina N, Sevastyanov V, Zazovskaya E, van der Plicht J. Reservoir Effect of Archaeological Samples from Steppe Bronze Age Cultures in Southern Russia. *Radiocarbon* **56**, 767–778 (2014).

21. Shishlina NI, Orfinskaya OV, Golikov VP. Bronze Age Textiles from the North Caucasus: New Evidence of Fourth Millennium BC Fibres and Fabrics. *Oxford Journal of Archaeology* **22**, 331–344 (2003).

22. Hansen S. Technische und soziale Innovationen in der zweiten Hälfte des 4. Jahrtausends v. Chr. In: *Sozialarchäologische Perspektiven: gesellschaftlicher Wandel 5000 - 1500 v. Chr. zwischen Atlantik und Kaukasus* (eds Hansen S). (von Zabern, Darmstadt, 2011).

23. Gadzhiev MG, Kohl PL, Magmedov RG, Stonach D. The 1995 Daghestan-American Velikent Expedition. Excavations in Daghestan, Russia. *Eurasia Antiqua* **3**, 181-222 (1997).

24. Reinhold S, Korobov DS, Belinskij AB. *Landschaftsarchäologie im Nordkaukasus: Studien zu einer neu entdeckten bronzezeitlichen Kulturlandschaft im Hochgebirge des Nordkaukasus*. (Habelt-Verlag, Bonn, 2017).

25. Korenevskiy SN, Kalmykov AA. Maykopskie pogrebeniya kurgana 22 mogilnika Aygurskiy 2. *Rossiyskaya Arkheologiya*, 77–94 (2017).

26. Gerasimova MM, Pezhemskiy DV, Jablonskiy LT. Paleoantropologicheskie materialy maykopskoy epokhi iz Zentralnogo Predkavkazya. *Materialy po Izucheniyu Istoriko-Kulturnogo Naslediya Severnom Kavkaza*, 91–121 (2007).

27. Hollund HI, Higham T, Belinskiy AB, Korenevskiy SN. Investigation of palaeodiet in the North Caucasus (South Russia) Bronze Age using stable isotope analysis and AMS dating of human and animal bones. *Journal of Archaeological Science* **37**, 2971–2983 (2010).

28. Korenevskiy SN, Atabiev BC, Batchaev VM. Pogrebeniya maykopsko-novosvobodenskoy obshchnosti mogilnika Goryachevodskiy. In: *Problemy drevney istorii i kultury Severnogo Kavkaza* (eds Munchaev RM, Korenevskiy SN). (Grif i K, Moscow, 2004).

29. Heussner K-U, Belinskiy A, Reinhold S, Kantorovich AR, Maslov VE. Dendrochronology of Bronze Age tombs: Poster at the Humboldt-Kolleg 'At the Northern Frontier of Near Eastern Archaeology, Venice January 9th to January 12, 2013. (eds). (Ca'Foscari University of Venice, Venice, 2013).

30. Korenevskiy SN, Belinskiy AB, Kalmykov AA. *Bolshoy Ipatovskiy kurgan na Stavropole: kak archeologicheskiy istochnik po epokhe bronzovogo veka na stepnoj granice Vostochnoy Evropy i Kavkaza*. (Nauka, Moscow, 2007).

31. Belinskij AB, Kalmykov AA, Korenevskiy SN, Härke H. The Ipatovo kurgan on the North Caucasian Steppe (Russia). *Antiquity* **74**, 773–774 (2000).

32. Belinskij AB, Kalmykov AA. Neue Wagenfunde aus Gräbern der Katakombengrabkultur im Steppengebiet des zentralen Vorkaukasus. In: *Rad und Wagen* (eds Fansa M). (von Zabern, Mainz am Rhein, 2004).

33. Fuchs K. Interdisciplinary analysis of the cemetery Kudachurt 14. Evaluating indicators of social inequality, oral health and diet during the Bronze Age key period between 2200-1600 BCE in the Northern Caucasus. Faculty of Mathematics and Natural Sciences. (eds). (Kiel University, Kiel, 2018 (submitted)).

34. Kantorovich AR, Maslov VE. Eine reiche Bestattung der Majkop-Kultur aus einem Kurgan bei der stanica Mar'inskaja, rajon Kirov, kraj Stavropol, Nordkaukasien: Vorläufiger Grabungsbericht. *Eurasia Antiqua*, 151–165 (2008).

35. Kantorovich AR, Maslov VE, Petrenko VG. Pogrebeniya maykopskoj kultury kurgana No. 1 mogilnika Marinskaja 5. *Materialy po Izucheniyu Istoriko-Kulturnogo Naslediya Severnom Kavkaza* **10**, 71–108 (2013).

36. Gresky J*, et al.* New cases of trepanations from the 5th to 3rd millennia BC in Southern Russia in the context of previous research: Possible evidence for a ritually motivated tradition of cranial surgery? *American journal of physical anthropology* **160**, 665–682 (2016).

37. Rostunov VL. *Epokha eneolita - sredney bronzy Centralnogo Kavkaza III: Opyt rekonstrukcii sakralnogo postranstva rannikh kurganov Evropy i Severnogo Kavkaza*. (Izdatelstvo SOGU, Vladikavkaz, 2007).

38. Rostunov VL. *Opyt rekonstrukcii sakralnogo postranstva rannikh kurganov Evropy i Severnogo Kavkaza*. (Izdatelstvo SOGU, Vladikavkaz, 2007).

39. Korenevskiy SN. Große Kurgane der Majkop-Kultur Arbeitsaufwand und kultische Aspekte bei ihrer Errichtung. In: *Von Majkop bis Trialeti* (eds Hansen S, Hauptmann A, Motzenbäcker I, Pernicka E). (Habelt-Verlag, Bonn, 2010).

40. Andrades Valtueña A*, et al.* The Stone Age Plague and Its Persistence in Eurasia. *Current biology : CB* **27**, 3683-3691.e3688 (2017).

41. Yakovlev AV, Samoylenko VG. Novye pogrebeniya s Maikopskoy keramikoy na severo-vostoke Stavropolya. *Kratkie Soobshchenija Instituta Arkheologii*, 74–83 (2003).

42. Tucker K, Berezina N, Reinhold S, Kalmykov A, Belinskiy A, Gresky J. An accident at work? Traumatic lesions in the skeleton of a 4th millennium BCE "wagon driver" from Sharakhalsun, Russia. *Homo : internationale Zeitschrift fur die vergleichende Forschung am Menschen* **68**, 256–273 (2017).

43. Erlikh VR, Kovalev DS, Maslov VE. Pogrebeniya epokhi bronzy kurgannogo mogilnika 'Sinyukha' v Adygee (predvaritelnye dannye). In: *Shestaya Kubanskaya Arkheologicheskaya Konferenciya* (eds). (Ekoinvest, Krasnodar, 2013).

44. Trifonov VA. Zapadne predeli rasprostraneniya maykopskoy kultury. *Izvestiya Samarskogo Nauchnogo Centra Rossiyskoy Akademii Nauk* **16**, 276–284 (2014).

45. Trifonov VA. Adaptaciya kultur paleolita - eneolita k izmeniyam prirodnoy sredy na Severo-Zapadnom Kavkaze. (eds). (Institut Istorii Materielnoy Kultury RAN, Sankt Petersburg, 2009).

46. Lovpache NG. Unakozovskie peshchery - pamyatnik protomaiotskoy kultury.: XVII Krupnovskie Chteniya, Maykop 1992. *Materialy po Izucheniyu Istoriko-Kulturnogo Naslediya Severnom Kavkaza* **8**, 468 (2008).

47. Potekhina ID. Antropologicheskie materialy iz pogrebeniy rannego mednogo veka v Unakozovskoy peshchere. In: *Arkheologiya Adygeya* (eds). (Meoty, Maykop, 1997).

48. Rasmussen M*, et al.* An Aboriginal Australian genome reveals separate human dispersals into Asia. *Science* **334**, 94-98 (2011).

49. Korneliussen TS, Albrechtsen A, Nielsen R. ANGSD: Analysis of Next Generation Sequencing Data. *BMC Bioinformatics* **15**, 1-13 (2014).

50. Lipatov M, Sanjeev K, Patro R, Veeramah K. Maximum Likelihood Estimation of Biological Relatedness from Low Coverage Sequencing Data. *bioRxiv*, (2015).

51. Monroy Kuhn JM, Jakobsson M, Gunther T. Estimating genetic kin relationships in prehistoric populations. *PLoS One* **13**, e0195491 (2018).

52. Fu Q*, et al.* The genetic history of Ice Age Europe. *Nature* **534**, 200-205 (2016).

53. Li H*, et al.* The Sequence Alignment/Map format and SAMtools. *Bioinformatics* **25**, 2078-2079 (2009).

54. Loh P-R, *et al.* Inferring admixture histories of human populations using linkage disequilibrium. *Genetics* **193**, 1233-1254 (2013).

55. Fenner JN. Cross-cultural estimation of the human generation interval for use in genetics-based population divergence studies. *Am J Phys Anthropol* **128**, 415-423 (2005).

56. Reich D, Thangaraj K, Patterson N, Price AL, Singh L. Reconstructing Indian population history. *Nature* **461**, 489-494 (2009).

57. Patterson N, *et al.* Ancient admixture in human history. *Genetics* **192**, 1065-1093 (2012).

58. Lazaridis I, *et al.* Ancient human genomes suggest three ancestral populations for present-day Europeans. *Nature* **513**, 409-413 (2014).

59. Mathieson I, *et al.* Genome-wide patterns of selection in 230 ancient Eurasians. *Nature* **528**, 499-503 (2015).
