# Supplementary material for: Ancient human genome-wide data from a 3000-year interval in the Caucasus corresponds with eco-geographic regions
Source: Nat Commun. 2019 Feb 4;10:590. doi: 10.1038/s41467-018-08220-8 (PMC6360191; doi:10.1038/s41467-018-08220-8)
Supplement: Supplementary file 3 — Description of Additional Supplementary Files [file 41467_2018_8220_MOESM3_ESM.pdf]

Supplementary Data 1. Summary of contextual archaeological and laboratory information for all newly analyzed prehistoric individuals in this study.

Supplementary Data 2. Summary of comparative ancient and modern datasets used in this study.

Supplementary Data 3. Full ADMIXTURE results from all ancient and relevant modern populations (k=2 to k=18).
