# Supplementary material for: Ancient human genome-wide data from a 3000-year interval in the Caucasus corresponds with eco-geographic regions
Source: Nat Commun. 2019 Feb 4;10:590. doi: 10.1038/s41467-018-08220-8 (PMC6360191; doi:10.1038/s41467-018-08220-8)
Supplement: Supplementary file 6 — Supplementary Data 3 [file 41467_2018_8220_MOESM6_ESM.pdf]

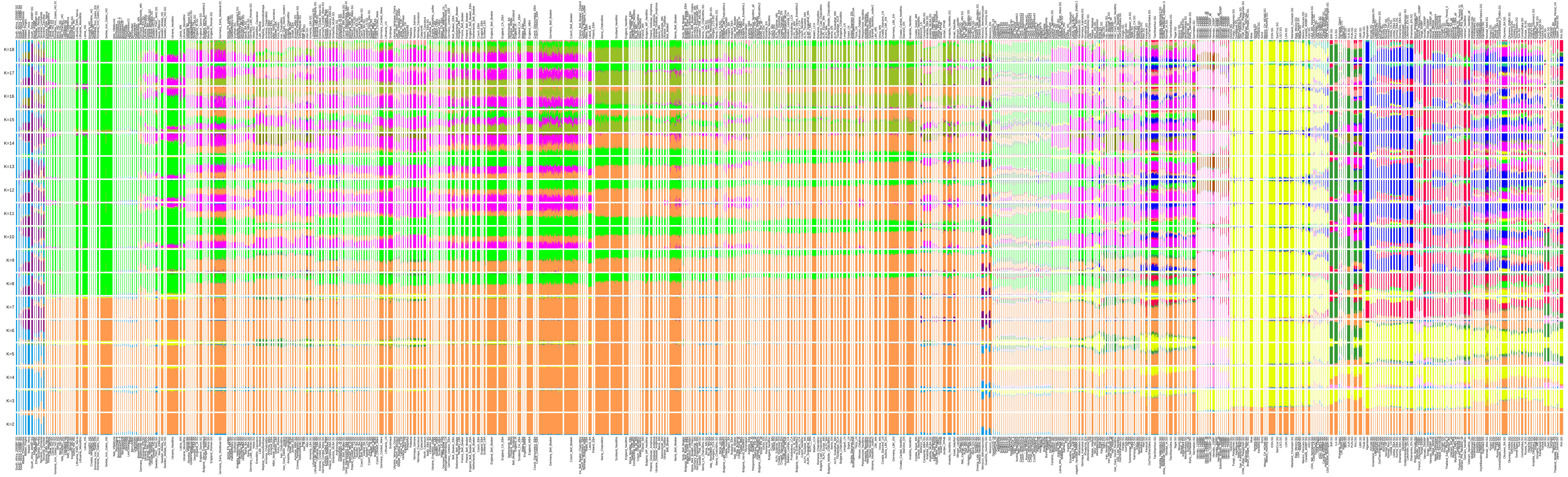
K=18
K=17
K=16
K=15
K=14
K=13
K=12
K=11
K=10
K=9
K=8
K=7
K=6
K=5
K=4
K=3
K=2
